# Analysis of Genetic Diversity and Population Structure of Rice Germplasm from North-Eastern Region of India and Development of a Core Germplasm Set

**DOI:** 10.1371/journal.pone.0113094

**Published:** 2014-11-20

**Authors:** Debjani Roy Choudhury, Nivedita Singh, Amit Kumar Singh, Sundeep Kumar, Kalyani Srinivasan, R. K. Tyagi, Altaf Ahmad, N. K. Singh, Rakesh Singh

**Affiliations:** 1 Division of Genomic Resources, National Bureau of Plant Genetic Resources, New Delhi, 110 012, India; 2 Division of Germplasm Conservation, National Bureau of Plant Genetic Resources, New Delhi, 110 012, India; 3 Department of Botany, Faculty of Science, Jamia Hamdard (Hamdard University), New Delhi, 110062, India; 4 National Research Centre on Plant Biotechnology, Indian Agricultural Research Institute, New Delhi, 110 012, India; National Institute of Plant Genome Research (NIPGR), India

## Abstract

The North-Eastern region (NER) of India, comprising of Arunachal Pradesh, Assam, Manipur, Meghalaya, Mizoram, Nagaland and Tripura, is a hot spot for genetic diversity and the most probable origin of rice. North-east rice collections are known to possess various agronomically important traits like biotic and abiotic stress tolerance, unique grain and cooking quality. The genetic diversity and associated population structure of 6,984 rice accessions, originating from NER, were assessed using 36 genome wide unlinked single nucleotide polymorphism (SNP) markers distributed across the 12 rice chromosomes. All of the 36 SNP loci were polymorphic and bi-allelic, contained five types of base substitutions and together produced nine types of alleles. The polymorphic information content (PIC) ranged from 0.004 for Tripura to 0.375 for Manipur and major allele frequency ranged from 0.50 for Assam to 0.99 for Tripura. Heterozygosity ranged from 0.002 in Nagaland to 0.42 in Mizoram and gene diversity ranged from 0.006 in Arunachal Pradesh to 0.50 in Manipur. The genetic relatedness among the rice accessions was evaluated using an unrooted phylogenetic tree analysis, which grouped all accessions into three major clusters. For determining population structure, populations K = 1 to K = 20 were tested and population K = 3 was present in all the states, with the exception of Meghalaya and Manipur where, K = 5 and K = 4 populations were present, respectively. Principal Coordinate Analysis (PCoA) showed that accessions were distributed according to their population structure. AMOVA analysis showed that, maximum diversity was partitioned at the individual accession level (73% for Nagaland, 58% for Arunachal Pradesh and 57% for Tripura). Using POWERCORE software, a core set of 701 accessions was obtained, which accounted for approximately 10% of the total NE India collections, representing 99.9% of the allelic diversity. The rice core set developed will be a valuable resource for future genomic studies and crop improvement strategies.

## Introduction

Plant genetic resources are of paramount importance for the future and to ensure the food and nutritional security of an increasing population. A large number of genetic materials have been conserved in Genebank, but their use is being limited due to an unmanageable number of accessions and the continuous expansion of accessions numbers. Core germplasm development has been proposed for better management and use of collections available in Genebank [1]. This requires the development of a core set of accession to more precisely characterize, explore, and conserve Genebank resources, monitor the genetic drift during preservation, and identify gaps in genetic diversity [2], [3]. The idea of the core set was proposed by Frankel and Brown in 1984 [2]. As proposed, a core set is a small set of accessions (usually 10 % of the population) chosen to represent the genetic spectrum of an entire collection [4].

The sampling percentage of a core set has long been under debate [5]; 20%–30% of the sampling percentage was suggested by Yonezawa *et al.* (1995) to adequately sample a given collection [6]. Mini core sets representing ∼1% of total collections and have also been used to characterize very large collections [7]–[10]. In fact, a mini core subset from USDA rice gene bank has been developed using 26 phenotypic trait and 70 molecular markers [11]. Still the perfect ratio and fixed size for all core set does not exist, since different crops or different constructing goal needs different sampling percentages [5]. Several methods have been used to develop core set. The stratified random sampling method, in contrast to the simple random sampling method, has been successfully applied to the development of numerous core set, e.g., fodder crops, potatoes, etc. [12]–[16]. One common approach for constructing a core set is grouping whole collections into major ecotypes and then selecting representatives from each ecotype [5], [17]–[18]. However, a core set formed for the purpose of capturing accessions with rare or extreme values of the desired trait(s) (e.g. high resistance to pest or high yield and yield contributing traits) should be evaluated separately with the intention of representing the (pattern of) genetic diversity in the collection. Indeed, the pattern of genetic diversity will have genetic variations among all accessions that have been accumulated as a result of natural processes, species' characteristics and historical events [19]. Until now, there is no universally accepted method for constructing a core set as many factors affect representativeness of core set, such as sampling percentage, data type, number of traits observed, genetic diversity of germplasm, grouping method and sampling method [18], [20]–[21].

The rice genetic resource is an important source for rice breeding and makes a valuable contribution to global wealth and food security [22]. India is considered to be the origin of rice. Based on the phylo-geographical and archeological evidence it is suggested that rice was domesticated about 10,000 years ago from its wild ancestor *O. rufipogon* in the region South of Himalayan mountain range, likely in the present day eastern and North-Eastern (NE) India, extending eastward to Nepal, Myanmar and Thailand to Southern China [23]–[25]. The NE India is a large geographical area comprising of seven states namely Arunachal Pradesh, Assam, Manipur, Meghalaya, Mizoram, Nagaland and Tripura states, is home to a large number of indigenous rice varieties [26]. The geography of the NE states of India is unique, having snow capped peaks of the Himalayas, the ecological hot spots of the NE foothills and the Brahmaputra valley. High rainfall, humidity, varied topography and altitude, heavy natural selection pressures, environmental stresses have made the region rich both in floristic and crop diversities [27].

A large collection of the rice genetic resource from NE India is currently conserved at National Genebank (NGB), National Bureau of Plant Genetic Resources (NBPGR), New Delhi. Since these germplasm contains unique traits which can be exploited for future crop improvement programme but has not been properly characterized at molecular level. Therefore, in the present study this collection was analyzed (i) to study the genetic diversity and population structure of all 6,984 rice germplasm accessions using 36 SNP markers, which were developed and used earlier for diversity and population structure analysis of rice varieties [28] and (ii) to develop a core set representing the maximum diversity of unique NE India rice genetic resources. Taken together, the described core set can be used to effectively for genomic studies, in rice crop improvement and conservation programs in genebank.

## Materials and Methods

### Plant materials

A total of 6,984 accessions of NE India (Arunachal Pradesh, Assam, Manipur, Meghalaya, Mizoram, Nagaland and Tripura,) were drawn from NGB, NBPGR, New Delhi. The NE India germplasm collection also included 24 accessions with EC ID from IRRI, Philippines and two accessions from Ghana which were growing in farmer's field since their introduction in 1991. The purpose of their inclusion was to analyse their clustering in relation to Indian germplasm collection. The details of each accession along with passport information were obtained from the online database of NGB, (www.nbpgr.ernet.in) and presented in Table S1.

### DNA extraction from rice seed

Seeds of each genotype (10–12 seeds) were dehusked and used for DNA isolation using QIAGEN DNeasy plant mini kit. Kernels were ground into fine powder using tissue lyser (Tissue lyser II Retsch, Germany) with a tissue lyser adapter set (QIAGENq). DNA was extracted following the procedures described by manufacturers.

### Genotyping of rice accessions using SNP markers

Genomic DNA of all the 6,984 accessions was diluted to prepare working stocks of 10 ng/µl. Sequenom Mass ARRAY multiplex assays were designed for 36 SNPs (iPLEX gold chemistry), representing conserved single-copy rice genes [29], taking three genes per rice chromosome [28]. These SNP markers are located on the short arm, centromeric region and long arm of the twelve rice chromosome. The 36-plex assay(s) were designed and validated by Sequenom Corporation (San Diego). The 30-mer pre-amplification primers and variable length genotyping primers generated by the Assay Design 3.1 software were procured and used for the validation of SNPs according to the Sequenom user manual. Mass ARRAY Typer 3.4 Software was used for the visualization of SNPs and allele calling. Chromosome number, primer ID and physical position of 36 SNPs used in this study are given in Table S2.

### Statistical analyses

The major allele frequency, gene diversity, heterozygosity and Polymorphic Information Content (PIC) for each locus were calculated for SNP markers using Power Marker 3.5 [30]. In addition, Principal Component Analyses (PCA), genetic distances [31] across the genotypes and neighbor-joining (NJ) tree were calculated using Power Marker 3.5 [30]. Phylogenetic trees for all states were constructed using MEGA software version 6.0 [32]. Principle Coordinate Analysis (PCoA) and Analysis of Molecular Variance (AMOVA) were performed using software GenAlEx V6.5 [33]. SNP data were numerically coded as follows: A = 1, C = 2, G = 3, T = 4 and missing data was coded as 0 as suggested in GenAlEx V6.5 user manual [33]. The model-based program, STRUCTURE 2.3.3 [34] was used to infer the population structure. For each K, three replications were run. Each run was implemented with a burn-in period of 100,000 steps followed by 100,000 Monte Carlo Markov Chain replicates [34] derived for each K and then plotted to find the plateau of the ΔK values [35]. The “Structure harvester” program was used (http://taylor0.biology.ucla.edu) to determine the final population. POWERCORE software [36] was used for the core development. Since numbers of rice accessions from each NE states were not equal, ranging from 107 accessions from Mizoram to 2,635 accessions from Assam. Therefore, to avoid the dominance of collections from individual states within the core set, we first developed core sub-sets using the rice collections from each state. Next, all accessions of the core sub-sets were pooled together to create the final core set of NE India rice germplasm.

## Results

### Genetic diversity

Genetic diversity of NE India rice collections (6,984 accessions), available at NGB, was estimated using 36 SNP markers. Alleles generated with all 36 SNP markers were scored to study the genetic diversity. Since these accessions cover seven states of NE India, they were analyzed state-wise for precise estimation of the level of their genetic diversity. The accessions collected from Arunachal Pradesh (663), Assam (2635), Manipur (549), Meghalaya (2427), Mizoram (107), Nagaland (377) and Tripura (226) were analyzed for major allele frequency, heterozygosity, gene diversity and PIC (Table 1). The highest mean major allele frequency was present in Manipur accessions (0.80) and lowest was found in Meghalaya accessions (0.74). The comparison of mean heterozygosity of alleles across the seven states revealed that Mizoram accessions were highly heterozygous (0.16); whereas heterozygosity in the accessions belonging to Arunachal Pradesh (0.07) and Nagaland (0.07) were recorded minimum (Table 1). The mean gene diversity across the seven states varied from 0.34 (in the collections from Meghalaya) to 0.26 in those from (Manipur) and PIC varied from 0.21 (in the collections from Manipur) to 0.27 (from Meghalaya). Out of 36 primers used for characterization of total rice collections, five SNP primers (01-608-4_C_375, 02-3029-1_C_474, 03-3478-1_C_206, 05-48-1_C_279 and 08-4218-5_C_129) exhibited the maximum PIC and gene diversity value 0.37 and 0.49, respectively (Table S2). The primer 04-19-4_C_240 was found to be the least informative with PIC and gene diversity values equaling 0.02 and 0.02, respectively, and major allele frequency of 0.99 (Table S2), SNP markers which showed low PIC values in the present study may not be considered for future studies on genetic diversity and population structure in rice. The maximum heterozygosity was observed with primer 04-1801-20_C_428 was 0.28, while the minimum heterozygosity was observed with primer 04-19-4_C_240 (0.01).

**Table 1:** Estimation of gene diversity, heterozygosity, PIC and major allele frequency in NE Indian rice collection (6984)

S. No.	States	No. of accessions	Gene diversity	Heterozygosity	PIC	Major allele frequency
1	Arunachal Pradesh	663	0.006–0.49 (0.29)	0.003–0.16 (0.07)	0.006–0.37 (0.23)	0.51–0.99 (0.78)
2	Assam	2635	0.01–0.5 (0.31)	0.01–0.32 (0.10)	0.01–0.37 (0.25)	0.50–0.99 (0.76)
3	Manipur	549	0.02–0.50 (0.26)	0.004–0.26 (0.09)	0.02–0.37 (0.21)	0.50–0.98 (0.80)
4	Meghalaya	2427	0.03–0.49 (0.34)	0.01–0.29 (0.12)	0.03–0.37 (0.27)	0.50–0.98 (0.74)
5	Mizoram	107	0.01– 0.49 (0.28)	0.01–0.42 (0.16)	0.01–0.37 (0.23)	0.52–0.99 (0.79)
6	Nagaland	377	0.008–0.49 (0.31)	0.002–0.16 (0.07)	0.008–0.37 (0.25)	0.52–0.99 (0.76)
7	Tripura	226	0.004–0.49 (0.28)	0.004–0.38 (0.08)	0.004–0.37 (0.22)	0.51–0.99 (0.79)
	Total	6984				

Mean values are given in parentheses.

Cluster analysis was performed using the NJ method for all the collections from seven states and unrooted phylogenetic trees were constructed (Fig. S1). Rice collections of each state were grouped in to three major clusters. Genetic distance for collections of Arunachal Pradesh varied from 0.00 to 0.75, Assam from 0.00 to 0.66, Manipur from 0.00 to 0.74, Meghalaya from 0.00 to 0.77, Mizoram from 0.00 to 0.68, Nagaland from 0.00 to 0.66, and Tripura from 0.00 to 0.67.

### Population structure

A model-based program, STRUCTURE, was used to determine the genetic relationship among individual rice accessions. The membership of each accession was run from K = 1 to K = 20 for all the collections from all seven states to estimate the number of populations. Structure Harvester (http://taylor0. biology.ucla.edu) was used to determine final number of populations. The number of populations in Meghalaya collections was estimated as five, in Manipur collections as four and three for collections belonging to remaining states, i.e. Arunachal Pradesh, Assam, Mizoram, Nagaland and Tripura (Fig. S2, Table S3). The collection from Arunachal Pradesh contained 265 pure and 114 admix individuals in population1, 65 pure and 16 admix individuals in population2 and 131 pure and 72 admix individuals were present in population3. The collection from Assam showed 300 pure and 329 admix in population1, 1073 pure and 568 admix in population2, 288 pure and 77 admix in population3. The Nagaland collections 61 pure and 10 admix were present in population1, 164 pure and 91 admix in population2 and 27 pure and 24 admix were present in population3. Tripura collections showed 14 pure and 1 admix in population1, 107 pure and 21 admix in population2, 54 pure and 29 admix in population3. The collections from Mizoram had 39 pure and 19 admix in population1, 4 pure and no admix in population2 and 31 pure and 14 admix in population3. Similarly, Manipur collections showed 100 pure and 63 admix in population1, 53 pure and 55 admix in population2, 22 pure and 29 admix in population3 and 100 pure and 128 admix in population4. The accessions included in the core are marked with boxes in the bar plot (Fig. S3).

### Analysis of molecular variance (AMOVA)

An AMOVA study of all rice accessions, belonging to seven different NE states, was performed to analyze the distribution of genetic diversity between and within the populations. The number of populations for each state was considered the same, as revealed by the population structure. AMOVA analyses showed that 21% diversity exists among populations in the collection from Arunachal Pradesh, while the collections from Assam, Manipur, Meghalaya, Mizoram, Nagaland and Tripura showed 24, 19, 29, 9, 8 and 22% diversity, respectively (Table 2). Maximum diversity of 29% among population was observed in Meghalaya collections, while minimum diversity of 8% was observed in Nagaland collections (Fig. S4). The highest level of diversity among individuals was present in Nagaland collections (75%) and the least diversity was observed in Meghalaya collections (46%).

**Table 2:** State-wise Analysis of Molecular Variance (AMOVA) of NE Indian rice collections.

States	Source	df	SS	MS	Est. Var.	%
Arunachal Pradesh	Among Pops	2	1160.573	580.286	1.523	21
	Among Indiv	660	6728.744	10.195	4.305	58
	Within Indiv	663	1051.000	1.585	1.585	21
	Total	1325	8940.317		7.413	100
Assam	Among Pops	2	4963.974	2481.987	1.751	24
	Among Indiv	2632	25094.849	9.535	3.836	51
	Within Indiv	2635	4908.500	1.863	1.863	25
	Total	5269	34967.323		7.449	100
Manipur	Among Pops	2	905.987	452.994	1.422	21
	Among Indiv	546	5785.991	10.597	4.509	58
	Within Indiv	549	866.500	1.578	1.578	21
	Total	1097	7558.478		7.510	100
Meghalaya	Among Pops	4	9032.582	2258.145	2.422	30
	Among Indiv	2422	23045.557	9.515	3.745	46
	Within Indiv	2427	4914.000	2.025	2.025	35
	Total	4853	36992.138		8.192	100
Mizoram	Among Pops	2	108.378	54.189	0.747	9
	Among Indiv	104	1247.813	11.998	4.569	56
	Within Indiv	107	306.000	2.860	2.860	35
	Total	213	1662.192		8.176	100
Nagaland	Among Pops	2	40.936	20.468	0.709	8
	Among Indiv	12	168.464	14.039	6.319	75
	Within Indiv	15	21.000	1.400	1.400	17
	Total	29	230.400		8.428	100
Tripura	Among Pops	2	400.316	200.158	1.564	22
	Among Indiv	223	2084.863	9.349	3.946	57
	Within Indiv	226	329.500	1.458	1.458	21
	Total	451	2814.679		6.967	100

### Principal coordinate analyses (PCoA)

PCoA revealed significant diversity in the NE India rice germplasm collections. Meghalaya collections, which exhibited the maximum number of population (k = 5) showed a very distinct PCoA plot, population1 in Meghalaya collections concentrated only in quadrant 3 and 4, whereas population4 concentrated in 1 and 2 quadrant and unlike population3, population4 and population5 did not show intermixing with each other (Fig. S5d). PCoA of collections from Arunachal Pradesh showed that population2 was very distinct, forming a separate group (Fig. S5a). PCoA plot of Assam, Manipur and Nagaland collections showed that all three populations were intermixed (Fig. S5b, Fig. S5c & Fig. S5f). In Mizoram collection population2 and Tripura collection population1 were highly distinct and formed a tight group in quadrant 1 and 4, respectively (Fig. S5e, Fig. S5g). The maximum cumulative variation (%) was observed for Nagaland rice collections with a value of 43.31% (Table 3); whereas, the minimum variation was found in Tripura accessions (32.53%). Overall, <44% variation were observed by first three components of principal coordinates, which indicates that accessions from all seven states were very diverse from one another.

**Table 3:** State-wise Principal Coordinate Analyses of NE Indian rice collections, with percentage of variation explained by the first 3 axes.

States	Per cent variation	1-axis	2-axis	3-axis
Arunachal Pradesh	%	26.74	9.23	7.30
	Cum %	26.74	35.97	43.27
Assam	%	20.79	8.30	6.50
	Cum %	20.79	29.09	35.58
Manipur	%	21.54	7.23	5.73
	Cum %	21.54	28.77	34.50
Meghalaya	%	22.45	11.45	5.55
	Cum %	22.23	33.54	39.05
Mizoram	%	19.10	7.96	6.62
	Cum %	19.10	27.06	33.68
Nagaland	%	24.95	11.05	7.30
	Cum %	24.95	36.01	43.31
Tripura	%	14.96	10.15	7.57
	Cum %	14.96	25.12	32.69

Cum- Cumulative.

### Development of Core Set

Of the 6,984 rice accessions studied, a core set of 701 accessions (i.e., 60 from Arunachal Pradesh, 259 from Assam, 60 from Manipur, 242 from Meghalaya, 14 from Mizoram, 35 from Nagaland, and 31 from Tripura) were selected using POWERCORE (Table S4). Nine allele types were produced by 36 SNP markers, of which four alleles were homozygous and five were heterozygous. Out of six types of substitutions possible (2 transitions and 4 transversion), five types of substitutions were observed and C/G or G/C type substitutions were not observed in our study. Allele frequency was calculated for all collections from seven states and core set (Table 4). A comparison of allele frequency showed that there was no loss of alleles in the resulting core set and they remained 99.9% identical.

**Table 4:** A comparison of percent of alleles generated in total collection vs core set.

	Arunachal Pradesh		Assam		Manipur		Meghalaya		Mizoram		Nagaland		Tripura	
Alleles	Total collection (663 acc.)	Core set (60 acc.)	Total collection (2635 acc.)	Core set (259 acc.)	Total collection (549 acc.)	Core set (60 acc.)	Total collection (2427 acc.)	Core set (242 acc.)	Total collection (107 acc.)	Core set (14 acc.)	Total collection (377 acc.)	Core set (35 acc.)	Total collection (226 acc.)	Core set (31 acc.)
AA	20.08	21.36	19.43	20.63	17.73	18.02	20.66	20.93	18.27	19.07	21.23	20.98	19.33	20.31
CC	21.16	17.68	18.23	18.44	13.51	15.91	16.98	17.22	15.82	16.92	16.83	17.44	18.43	16.96
GG	16.72	18.44	21.10	18.93	24.67	21.07	20.07	18.90	19.55	19.07	20.53	19.86	22.19	20.01
TT	37.83	32.12	34.94	30.20	38.62	34.43	35.17	30.64	36.55	31.70	37.11	31.33	35.32	33.82
CT/TC	1.57	3.70	2.31	3.87	2.14	3.71	2.67	4.45	3.66	4.29	1.59	3.78	2.21	3.81
GA/AG	1.53	3.65	2.32	4.36	2.00	3.94	2.42	4.25	3.76	5.48	1.49	3.17	1.46	3.05
TA/AT	0.33	0.78	0.46	0.96	0.49	0.86	0.56	1.08	0.83	1.31	0.26	0.75	0.37	0.61
GT/TG	0.70	2.00	1.07	2.20	0.80	1.83	1.29	2.10	1.46	1.91	0.87	2.24	0.67	1.22
CA/AC	0.07	0.27	0.14	0.41	0.04	0.22	0.17	0.43	0.10	0.24	0.09	0.47	0.03	0.20

### Genetic diversity of Core Set

**Figure 1:**
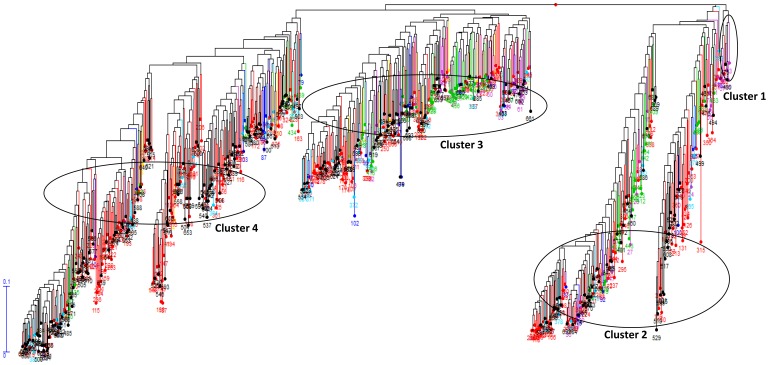
Phylogenetic tree of NE India rice core set (701 accessions) constructed based on SNP data. Germplasm accessions collected from the NE states are depicted with different colours Arunachal Pradesh - green, Assam - red, Manipur - purple, Meghalaya - black, Mizoram -yellow, Nagaland - light blue, Tripura - dark blue.

The genetic diversity of the core set was estimated to know the extent of diversity captured from total collection. Major allele frequency; gene diversity, heterozygosity and PIC were separately estimated for core-sets of individual states (Table 4). Comparisons of all genetic parameters (major allele frequency, gene diversity, heterozygosity and PIC) revealed that values for core set (Table 5) were greater than total collections (Table 1). For example, the Arunachal Pradesh gene diversity was 0.29, which increased to 0.39 in the core set. Similarly, the PIC for the Arunachal Pradesh collection was 0.23, while its core set PIC was 0.31. Cluster analysis showed that, core set accessions were getting distributed in to four clusters. The nine accessions with EC ID which were also part of core set were represented in three out of four clusters. The genetic diversity for the core set varied from 00.00 to 0.68 (Fig. 1).

**Table 5:** Estimation of gene diversity, heterozygosity, PIC and major allele frequency in NE India rice core set (701 accessions).

S. No.	States	core sample	Gene diversity	Heterozygosity	PIC	Major allele frequency
1	Arunachal Pradesh	60	0.0726–0.5000 (0.3962)	0.0377–0.2778 (0.1713)	0.0700–0.3750 (0.3124)	0.5000–0.9623 (0.6940)
2	Assam	259	0.1670–0.5000 (0.4018)	0.0843–0.3618 (0.1908)	0.1531–0.3740 (0.3172)	0.5000–0.9080 (0.69271)
3	Manipur	60	0.1372–0.5000 (0.3749)	0.0455–0.3455 (0.1745)	0.1278–0.3750 (0.2982)	0.5000–0.9259 (0.7142)
4	Meghalaya	242	0.2390–0.4995 (0.4100)	0.1013–0.3493 (0.2001)	0.2105–0.3747 (0.3231)	0.5161–0.8612 (0.6873)
5	Mizoram	14	0.0689–0.4970 (0.3506)	0.0714–0.4615 (0.2213)	0.0665–0.3735 (0.2813)	0.5385–0.9643 (0.7416)
6	Nagaland	35	0.1087–0.5000 (0.3854)	0.0385–0.3846 (0.1682)	0.1028–0.3846 (0.3049)	0.5000–0.9423 (0.7033)
7	Tripura	31	0.0351–0.5000 (0.3596)	0.0323–0.3667 (0.1524)	0.0345–0.3750 (0.2875)	0.5000–0.9821 (0.7344)

Mean values are given in parentheses.

### Population structure of Core Sets

**Figure 2:**
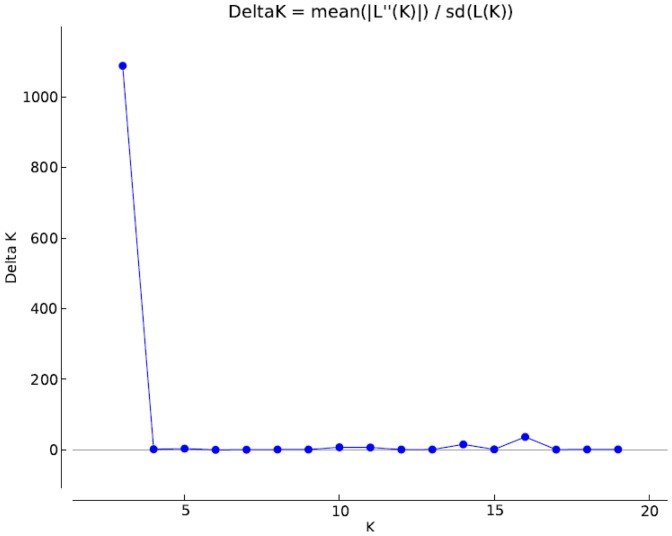
Estimation of populations in NE India rice core set using LnP(D) derived Δk for k from 1 to 20.

**Figure 3:**
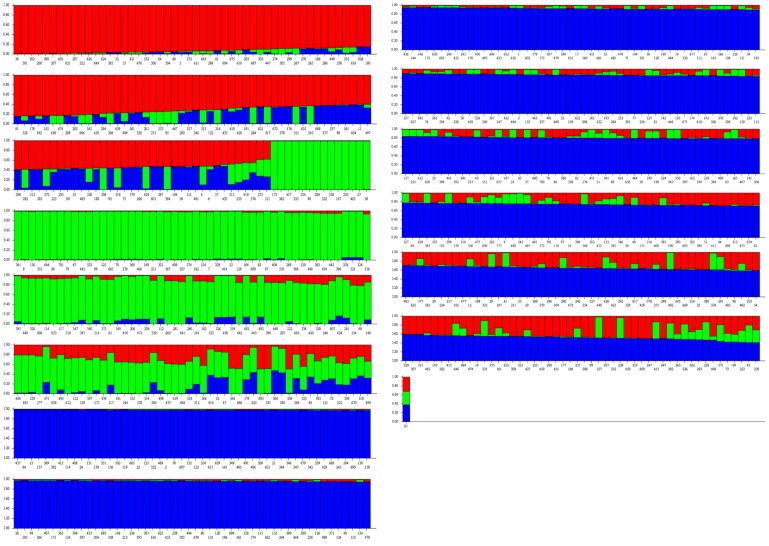
Model based clustering of the NE India rice core set (701 accessions).

STRUCTURE analysis grouped core set of 701 rice accessions into three populations (Fig. 2). Populations were studied for the number of pure and admix individuals. Population1 had 62 pure and 74 admix, population2 had 111 pure and 53 admix and population3 had 250 pure and 151 admix (Fig. 3).

### AMOVA and PCoA of Core Set

**Figure 4:**
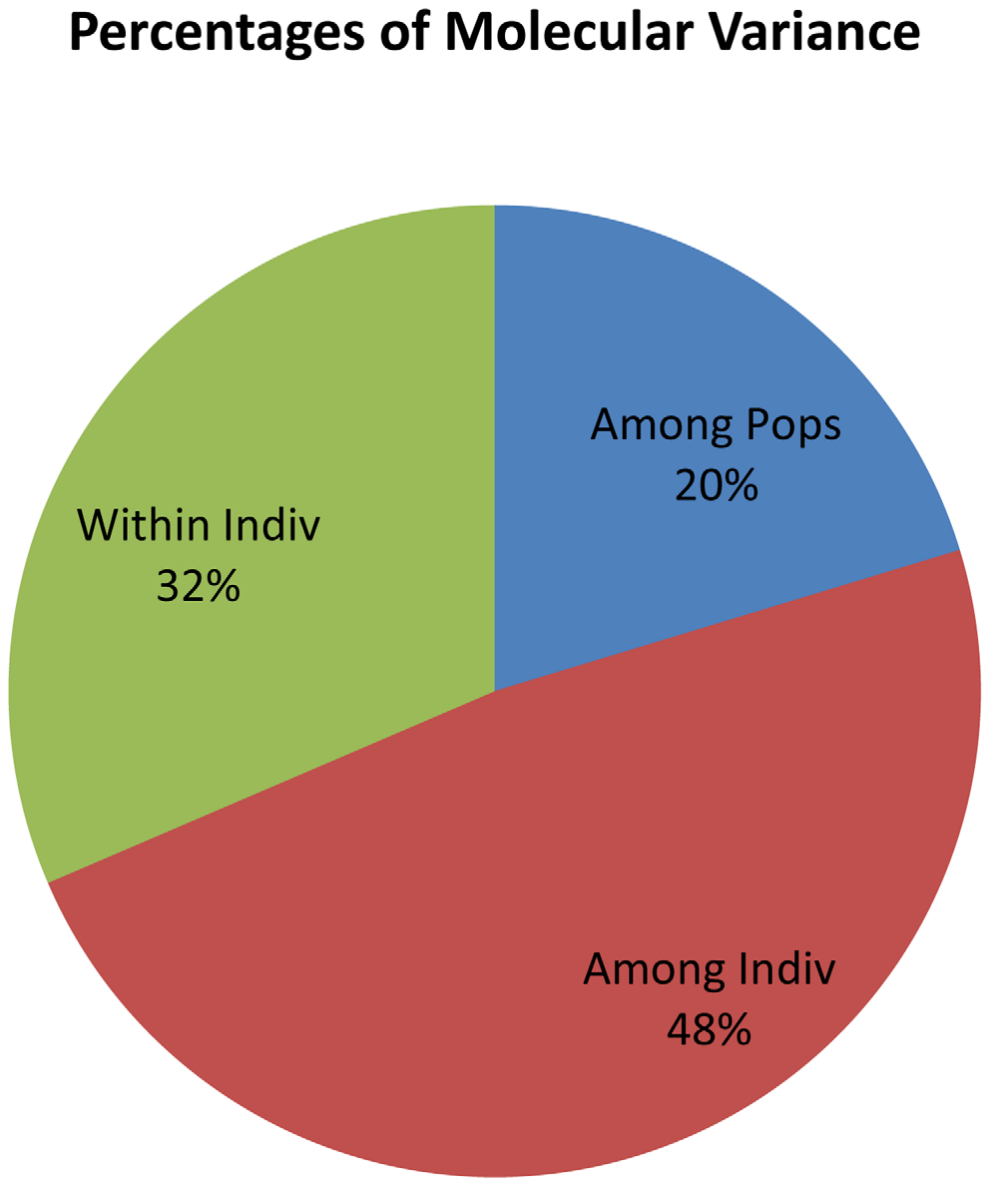
Analysis of Molecular Variance (AMOVA) analyses of NE India rice core set.

**Figure 5:**
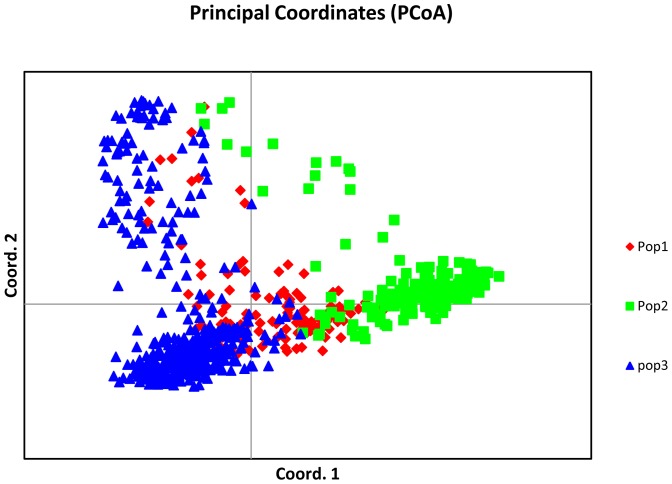
Principal Coordinate Analysis (PCoA) analyses of NE India rice core set.

**Figure 6:**
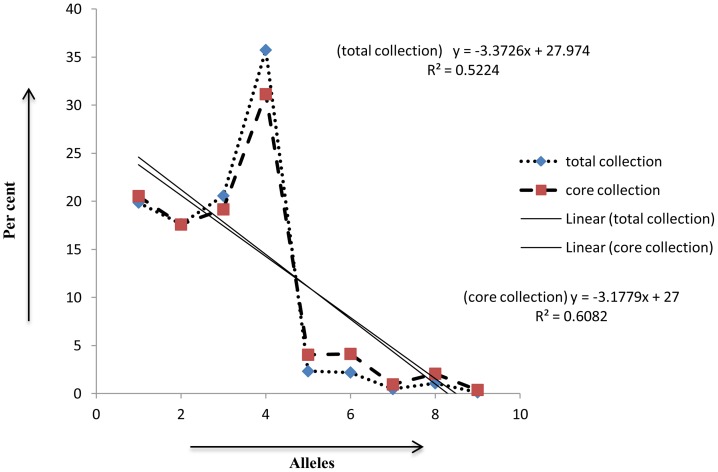
Comparison of allele frequency occurrence in total NE rice collections vs core set identified in the present study.

AMOVA analyses for core set showed 20% variance among populations (Table 6, Fig. 4); whereas, 48% variance was present among individuals. The partitioning of molecular variance was similar to the total collection and maximum diversity among individuals. PCoA could not be useful to identify significant isolation in populations except, for population2, which was tightly clustered in quadrant 3 and 4 (Fig. 5). Maximum cumulative percent variation was explained by the first three coordinates with 43.08% variance (Table 7). A plot of percent allele frequency of total collection *versus* core set showed that nearly all alleles with similar frequency were represented in the core collection (Fig. 6) and 99.9% alleles have been retained in the collections designated as core set.

**Table 6:** AMOVA of core set of NE Indian rice (701 accessions).

Source	df	SS	MS	Est. Var.	%
Among Pops	2	1649.152	824.576	1.996	20
Among Indiv.	698	8774.397	12.571	4.740	48
Within Indiv.	701	2166.500	3.091	3.091	32
**Total**	**1401**	**12590.049**		**9.826**	**100**

**Table 7:** Percentage of variation explained by the first 3 axes in Principal Coordinate Analyses of NE Indian rice core set (701 accessions).

Per cent variation	1-axis	2-axis	3-axis
%	18.41	18.04	6.63
Cum %	18.41	36.45	43.08

## Discussion

Genome level profiling of huge germplasm collections in crop species is essential to identify small and diverse sets of accessions for their efficient use in crop improvement programmes. Despite the advancement in genomics, the Indian rice collection remained uncharacterized at the molecular level, with respect to parameters such as genetic diversity and population structure. This has been the major limiting factor in their utilization and development of improved cultivars. The present study is the first major effort to characterize the NE India rice collections. This study shows that highly efficient and selective SNP markers can be used to enhance genome based analysis, e.g., genome wide association mapping.

State wise analysis of the genetic diversity of the NE India collection showed interesting results. The average PIC values for these SNP markers did not vary much across the states and ranged from 0.23 (Manipur) to 0.27 (Meghalaya). The average PIC value recorded in the present study was in the range reported by researchers in previous studies. Singh *et al*., (2013) [28] reported average PIC value 0.25 for the SNP markers in a set of 375 Indian rice varieties. The highest values for the parameters like PIC and gene diversity were found in collections from Meghalaya, which indicates the presence of a large rice genetic diversity in Meghalaya collections as compared to those from other six states. The same was also confirmed from PCoA, which displayed cluttered plot for the Meghalaya collection. The highest heterozygosity was observed in the collections from Mizoram, while Manipur had the highest major allele frequency.

Analysis of statistical genetic parameters across the collections from different states indicated a high level of variability in the NE India collection. There is relatively a high level of genetic diversity among the NE India rice collections, as reported by others using morphological [37] and molecular markers such as RAPD [38], ISSR [39] and SSR [26], [40]. Comparison of our results with previous findings revealed that PIC and genetic diversity were nearly half of the SSR-based studies which were on the expected line [26], [28], [40]. Similar results have been reported in other species that have used SSR and SNP markers [41], [42]. In the past, analysis of rice accessions from all seven states revealed three major clusters. Vairavan *et al*., (1973) [37] reported three clusters among a set 400 accessions of NE India rice collection based on morphological analysis. Das *et al*., (2013) [40] showed four groups were present in NE India rice varieties, while Choudhury *et al*., (2013) [26] reported only two groups, based on SSR marker analysis.

Population structure study has been reported for the first time in NE rice collections and the number of populations estimated five for Meghalaya, four for Manipur and three for the remaining states. Admixture has been reported in all the states besides pure lines and this may be due to germplasm taken in this study which is more heterogeneous in comparison to varieties.

There have been no other studies for characterization of a large number of NE India rice accessions at the molecular level that could be used for comparison of our results. AMOVA analysis showed maximum variation among individuals for accessions belonging to Nagaland (75%) and minimum for Meghalaya collection (37%). Choudhury *et al*., (2013) [26] conducted AMOVA analysis on NE rice, which revealed 25% variation among individuals, 66% among varieties and 9% among cultivated types. The variation among individuals found in our study was higher than that reported previously, which may be attributed to the large sample size used in present study. Also partitioning of variability at population and individual levels in AMOVA among states were different because sample size taken from different states varied from 107 accessions from Mizoram to 2,635 accessions from Assam.

The genomic characterization revealed huge variability among the NE India rice collection; therefore, we decided to identify a core set for the NE India accessions to facilitate the rice breeders to effectively use the same in their crop improvement programmes and efficient conservation and management of germplasm in the genebank. In recent past, various types of molecular markers have been used to develop core sets for different annual crops. These markers include SNPs for Arabidopsis [43], SSRs for rice, wheat and common bean [44]–[46], AFLPs for barley [47], RFLPs for wild wheat [48] and RAPD for common bean [49]. The present study used advanced SNP markers due to their biallelic nature, co-dominance inheritance and amenability to high throughput analysis. Another crucial step for development of core set is to formulate a suitable strategy that can identify either major and minor variances or diversity, using a fully representative accession from the large collections. One of the most commonly followed approach for constructing a core set from a collection is to rely on grouping an entire collection based on growing regions and then selecting core accessions from each group followed by combining accessions from each group to form final core set [5].

In the current study, we divided the NE India rice collection into seven (on the basis of source states) subsets and later all the accessions of subsets were pooled to form a core set of NE India rice germplasm. Statistical parameters, genetic distance and cluster analysis were taken into consideration to develop this core set; these methods were followed in accordance with other studies [4], [50], [51], [52], [53], [54]. We developed the NE India rice core set collection using SNP markers employing POWERCORE software (using advanced M strategy implemented through a modified heuristic algorithm). We relied on molecular data more than the passport and phenotypic data, because molecular markers can accurately represent the genetic diversity of the original collection and free of problems related to missing data or environmental interactions that are typically found in passport and phenotypic data. POWERCORE was used to develop a core set of 701 rice accessions (10% of total NE India rice collection). Nearly all alleles were represented in the core set; hence, there was no loss of alleles during the core set development.

In present study, we developed a core set of 10% of total collection; while there is no perfect core set constructing theory or universal methods to fit all crops, which might be due to large number of germplasm resources, absence of data for accessions, or complication and diversification of data type, and so on [50]. The core set was statistically supported with various other statistical parameters including genetic distance and cluster analysis that were taken into consideration in the final development of core set which is in accordance with previous studies [50]. The gene diversity value 0.54 and PIC value 0.48 estimated by SSR markers on rice core [22], is a higher range than the values found in our study (gene diversity value 0.39 and PIC value 0.31). This variation is obvious due to nature of marker used for development of core set. In present study, we have used SNP marker which is biallelic in nature whereas, Zhang et al., 2011, [22] have used SSR marker which is multi-allelic. A study conducted with SNP markers in tropical maize [51] reported a gene diversity value 0.29 and heterozygosity value 0.25 on core sets, and similar gene diversity was observed in present study. The gene diversity, heterozygosity, PIC values of core set were higher than those of total collection which was expected because the diversity increases with elimination of genetically similar accessions during core set development [11].

In conclusion, SNP marker based molecular characterization of NE India rice collection revealed that large variation exists among the accessions and the pattern of the genetic diversity and population structure varied across the seven states. Collections from Meghalaya were found to be the most diverse and possessed maximum number of populations. Core sets have been developed state wise as well as for the entire NE collection that can be potentially exploited by rice breeders in conventional breeding programmes and molecular breeders/researchers for genomic studies such as association mapping for gene discovery for various desirable traits and identification of suitable parents for their use in rice improvement. Further development of core set and sub-sets for NE India rice is likely to significantly reduce the task of the genebank curators as they will need to multiply and maintain small number accessions in the medium term storage as active collections to meet the requirement of the rice breeders.

## Supporting Information

Figure S1 **Circular phylogenetic trees of NE rice collection constructed based on SNP data using MEGA software (a) Arunachal Pradesh, (b) Assam, (c) Manipur, (d) Meghalaya, (e) Mizoram, (f) Nagaland and (g) Tripura. (Red marked accessions was selected for core by Power Core).**
(DOCX)

Figure S2 **Estimation of populations in NE rice collections using LnP(D) derived Δk for k from 1 to 20 in (a) Arunachal Pradesh, (b) Assam, (c) Manipur, (d) Meghalaya, (e) Mizoram, (f) Nagaland and (g) Tripura.**
(DOCX)

Figure S3 **Population structure of NE rice collection based on SNP data (a) Arunachal Pradesh, (b) Assam, (c) Manipur, (d) Meghalaya, (e) Mizoram, (f) Nagaland and (g) Tripura.**
(DOCX)

Figure S4 **Analysis of Molecular Variance in NE rice collection based on SNP data (a) Arunachal Pradesh, (b) Assam, (c) Manipur, (d) Meghalaya, (e) Mizoram, (f) Nagaland and (g) Tripura.**
(DOCX)

Figure S5 **Principal Coordinate Analysis in NE rice collection based on SNP data (a) Arunachal Pradesh, (b) Assam, (c) Manipur, (d) Meghalaya, (e) Mizoram, (f) Nagaland and (g) Tripura.**
(DOCX)

Table S1 **List of NE rice collections studied using SNP markers.**
(XLSX)

Table S2 **List of SNP primers used for genotyping of NE rice collection (6984 rice accessions) alongwith gene diversity, PIC and Major allele frequency.**
(DOCX)

Table S3 **Output Table generated from Structure Harvester software for Evanno method. Yellow highlight shows the largest value in the Delta K column.**
(DOCX)

Table S4 **List of rice accessions identified as NE core set based on SNP markers.**
(XLSX)
