# Supplementary figures and images for: Analysis of Genetic Diversity and Population Structure of Rice Germplasm from North-Eastern Region of India and Development of a Core Germplasm Set
Source: PLoS One. 2014 Nov 20;9(11):e113094. doi: 10.1371/journal.pone.0113094 (PMC4239046; doi:10.1371/journal.pone.0113094)

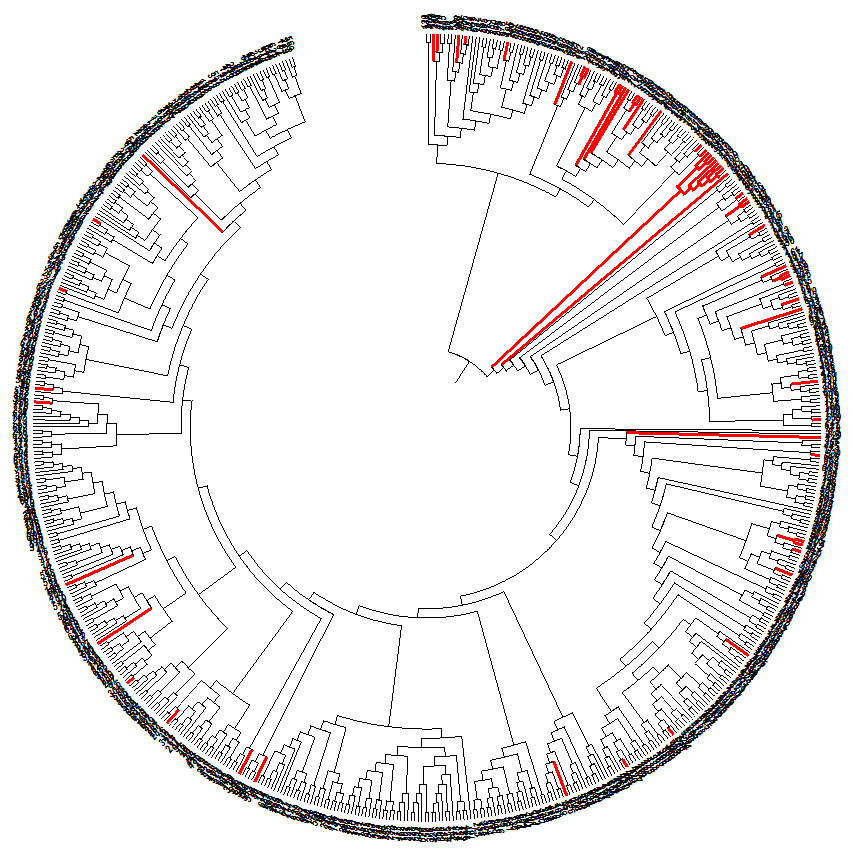


**Fig S1a**


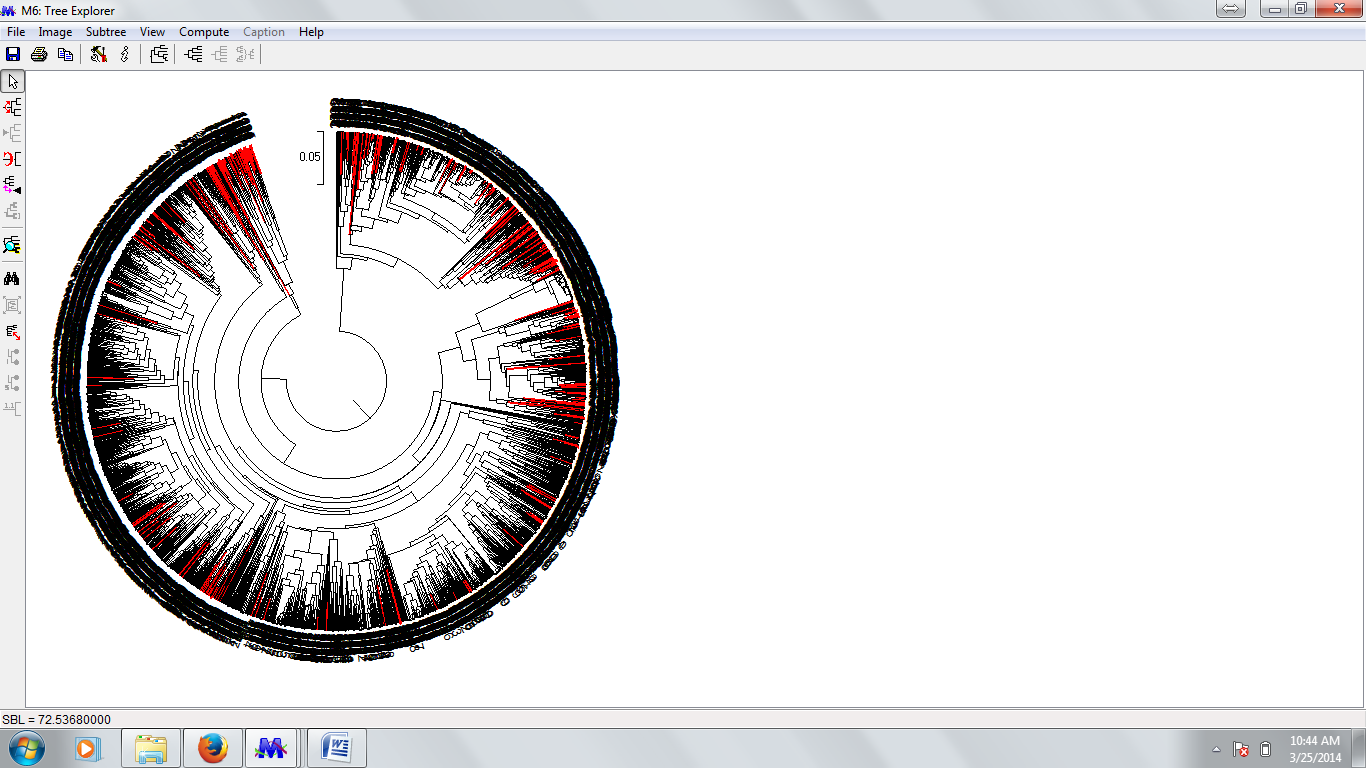


**Fig S1b**


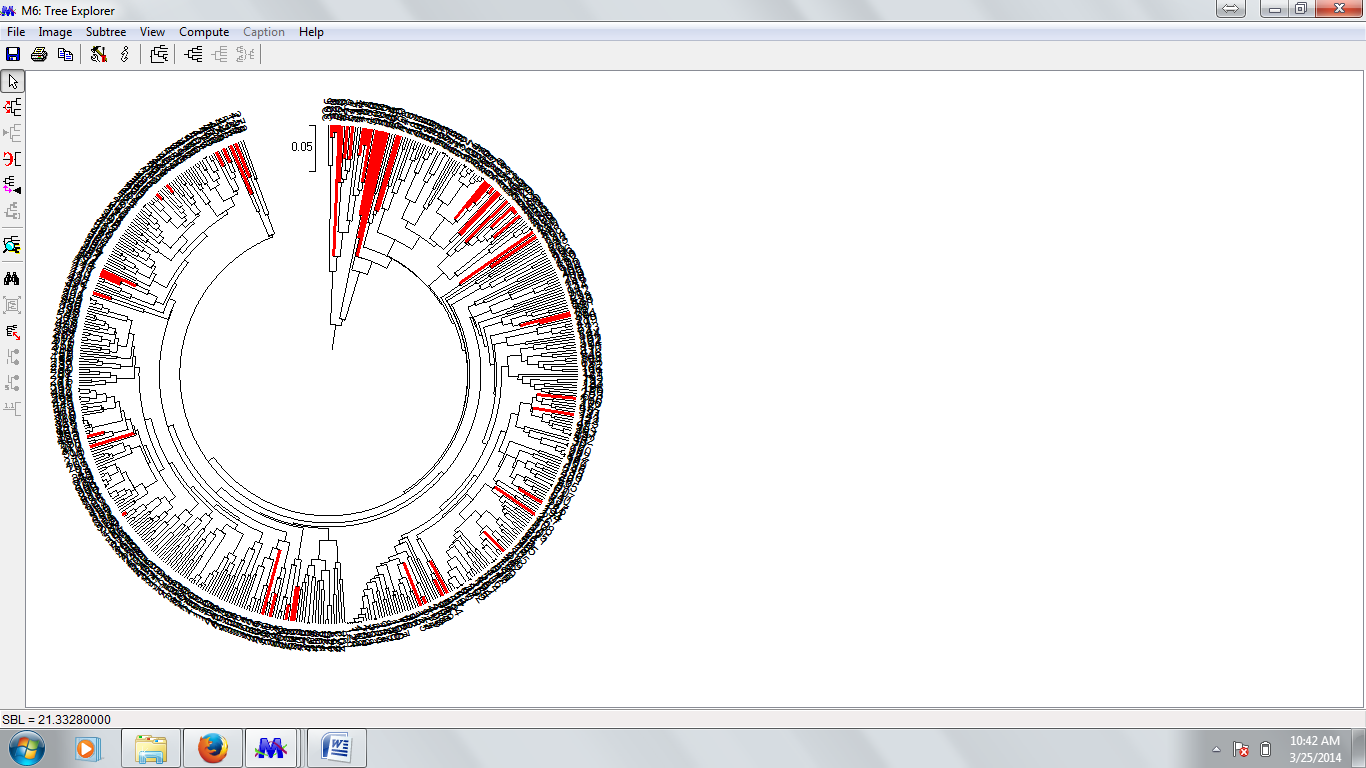
**Fig S1c**


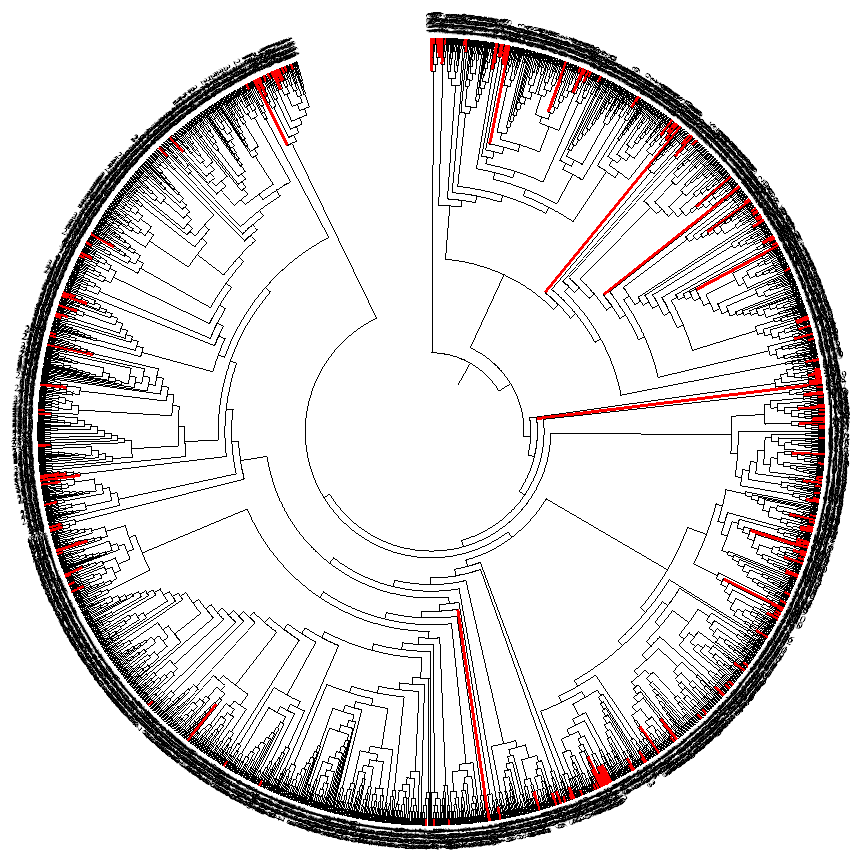


**Fig S1d**


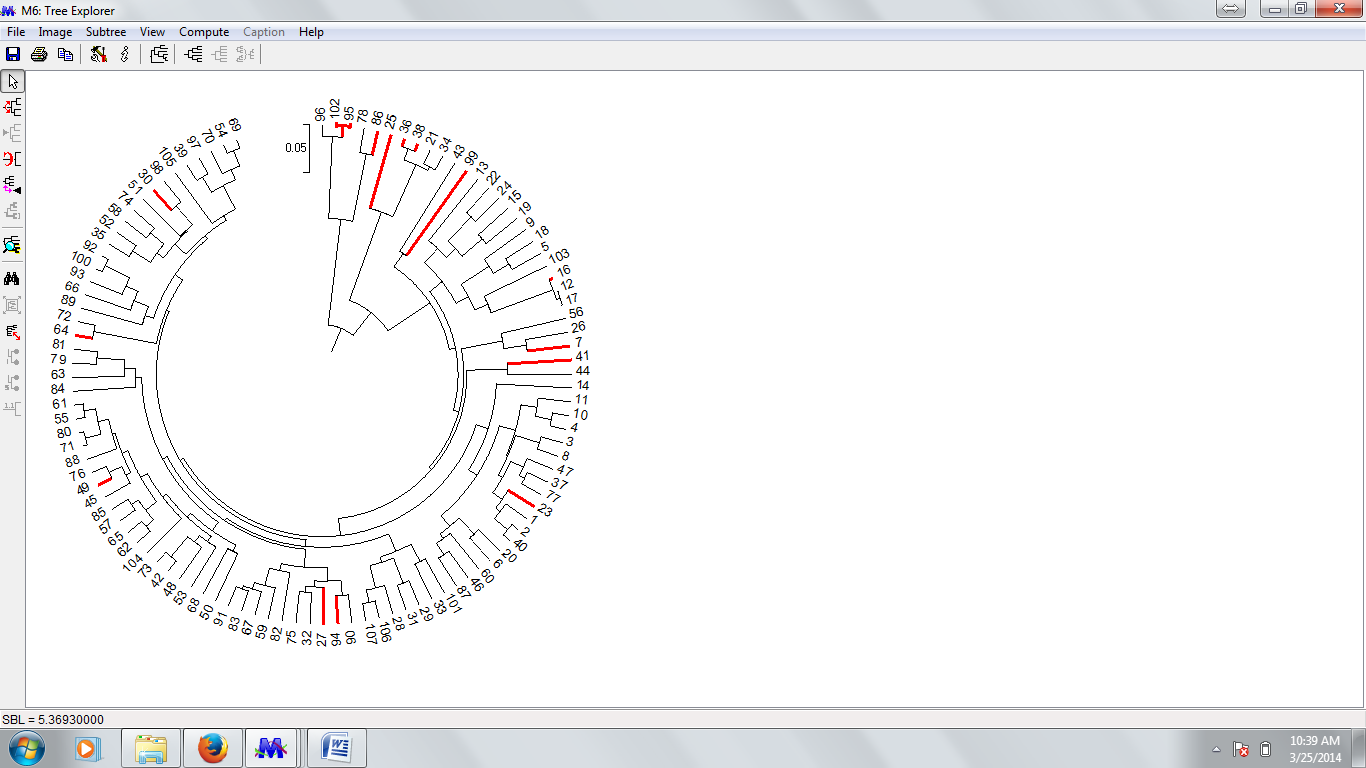


**Fig S1e**


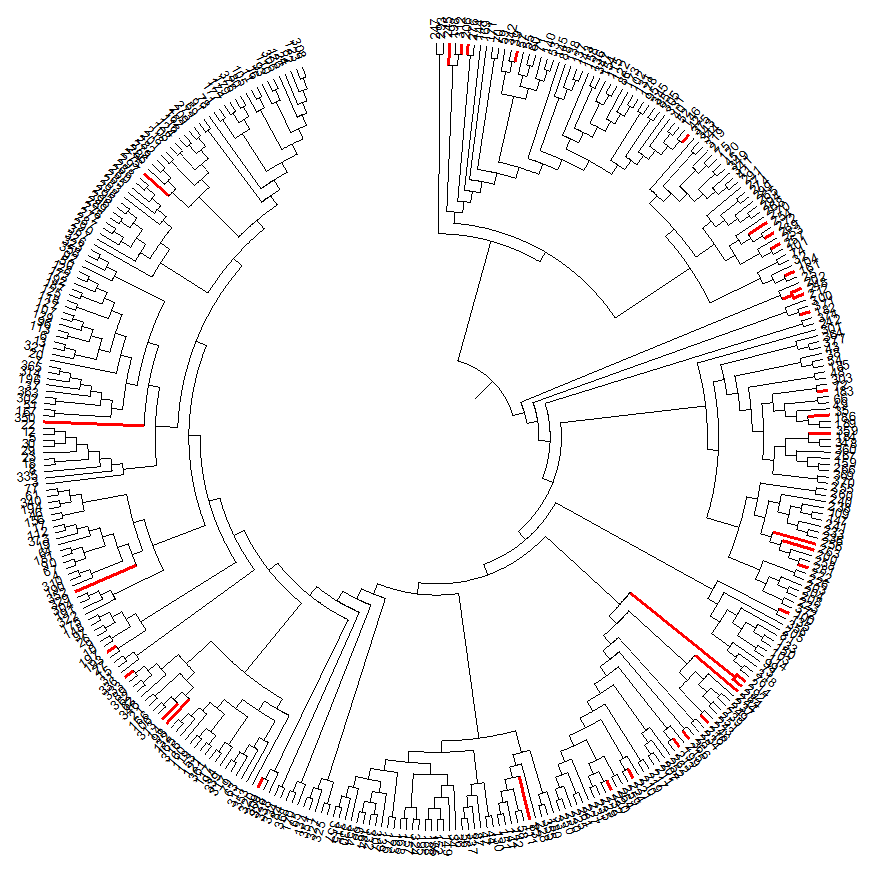


**Fig S1f**


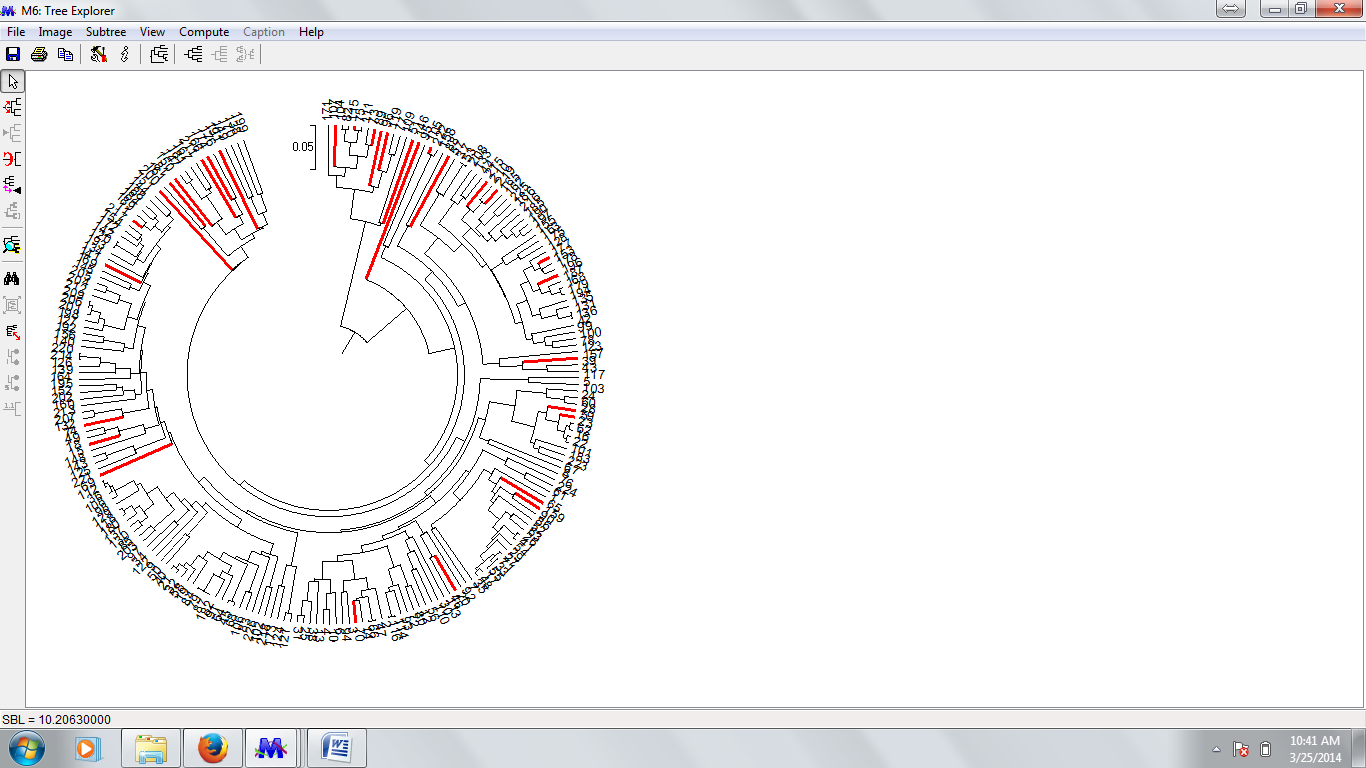


**Fig S1g**

Supplement: Figure S1 — Circular phylogenetic trees of NE rice collection constructed based on SNP data using MEGA software (a) Arunachal Pradesh, (b) Assam, (c) Manipur, (d) Meghalaya, (e) Mizoram, (f) Nagaland and (g) Tripura. (Red marked accessions was selected for core by Power Core). (DOCX) [file pone.0113094.s001.docx]

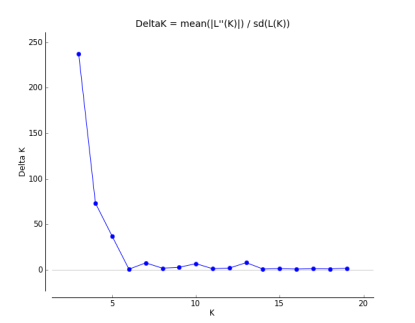

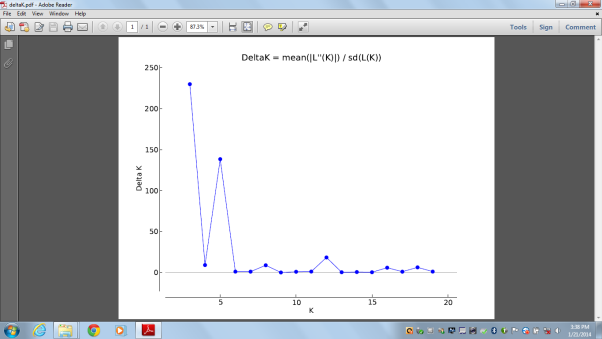


**Assam**

**Arunachal Pradesh**

1. **(b)**


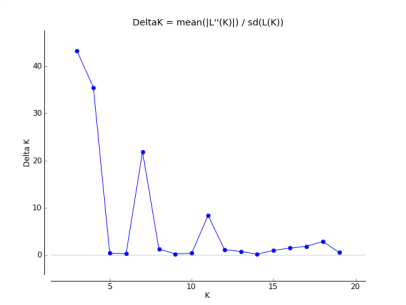


**Meghalaya**

**Manipur**

**(c) (d)**


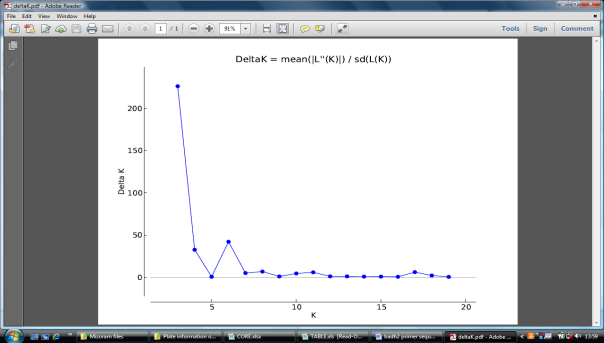

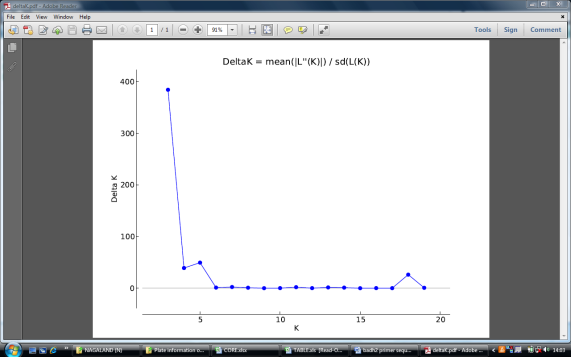


**Nagaland**

**Mizoram**

**(e) (f)**


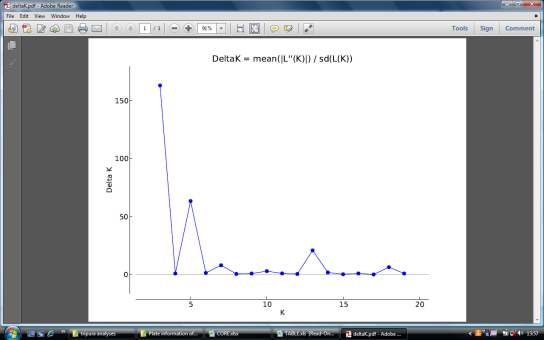


**Tripura**

**(g)**

**Fig S2**

Supplement: Figure S2 — Estimation of populations in NE rice collections using LnP(D) derived Δk for k from 1 to 20 in (a) Arunachal Pradesh, (b) Assam, (c) Manipur, (d) Meghalaya, (e) Mizoram, (f) Nagaland and (g) Tripura. (DOCX) [file pone.0113094.s002.docx]
