# Supplementary material for: Analysis of Genetic Diversity and Population Structure of Rice Germplasm from North-Eastern Region of India and Development of a Core Germplasm Set
Source: PLoS One. 2014 Nov 20;9(11):e113094. doi: 10.1371/journal.pone.0113094 (PMC4239046; doi:10.1371/journal.pone.0113094)

**Fig S3a Model based clustering of Arunachal Pradesh**


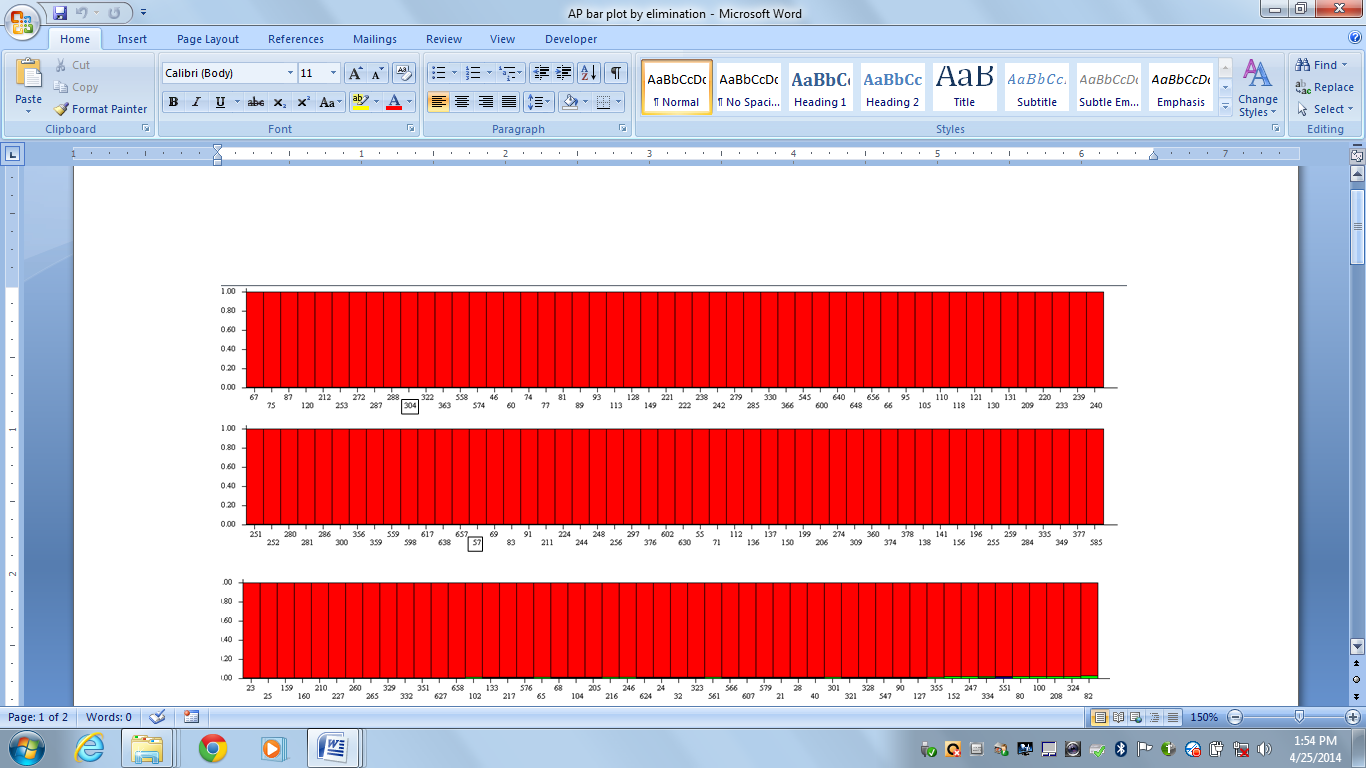


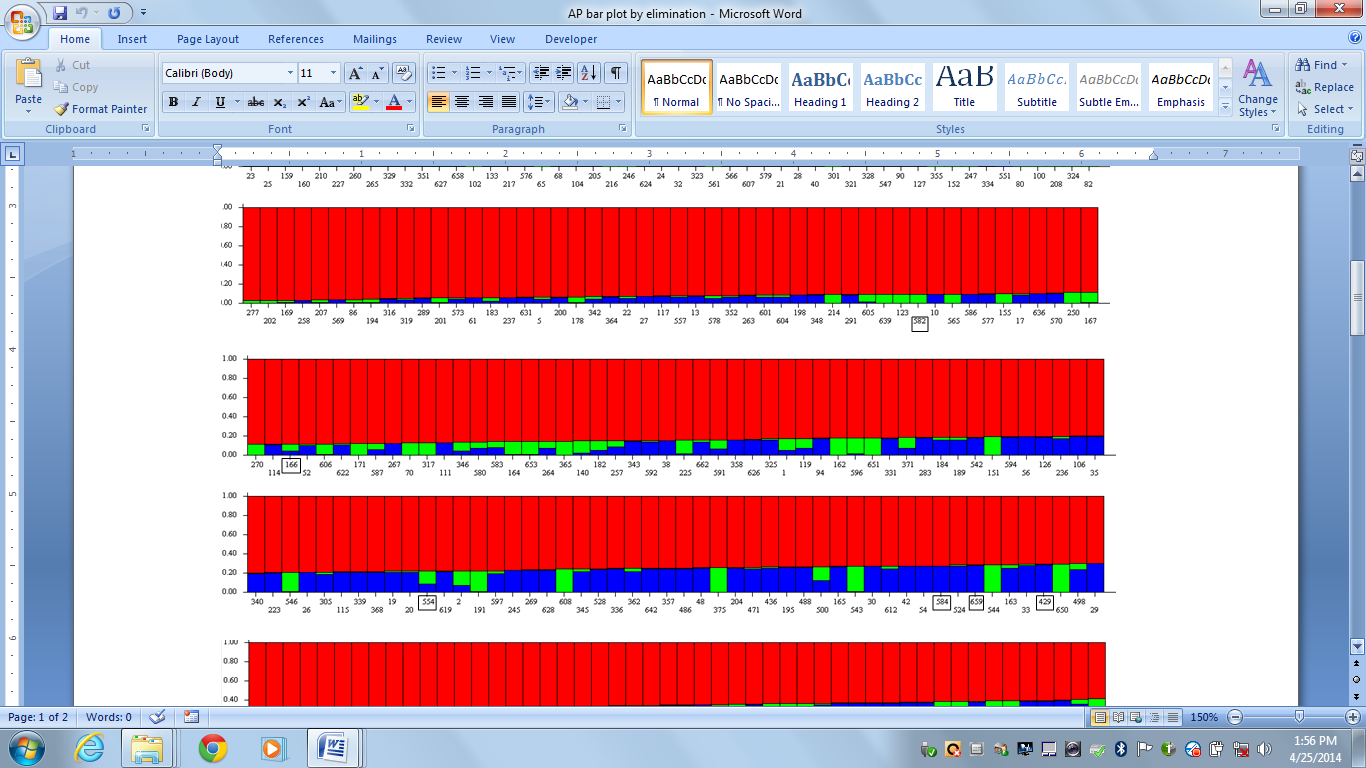


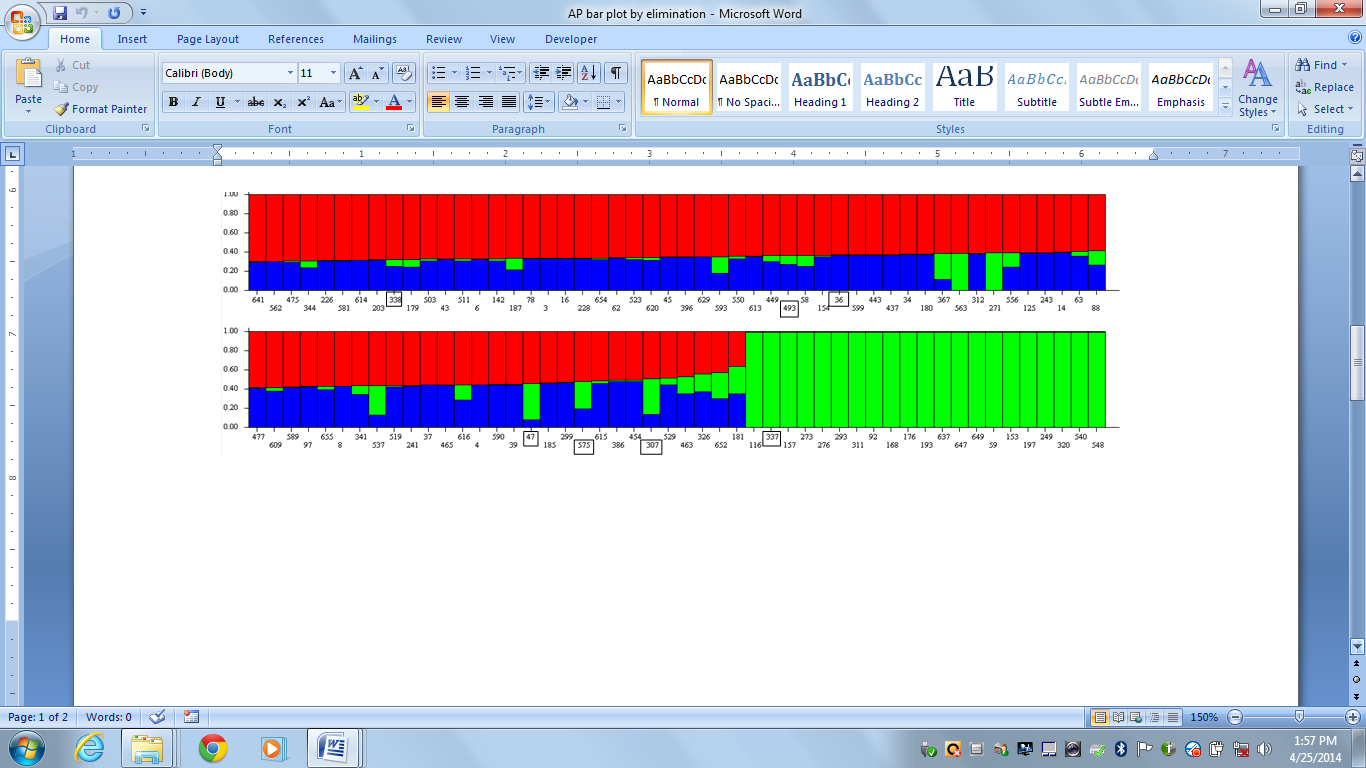

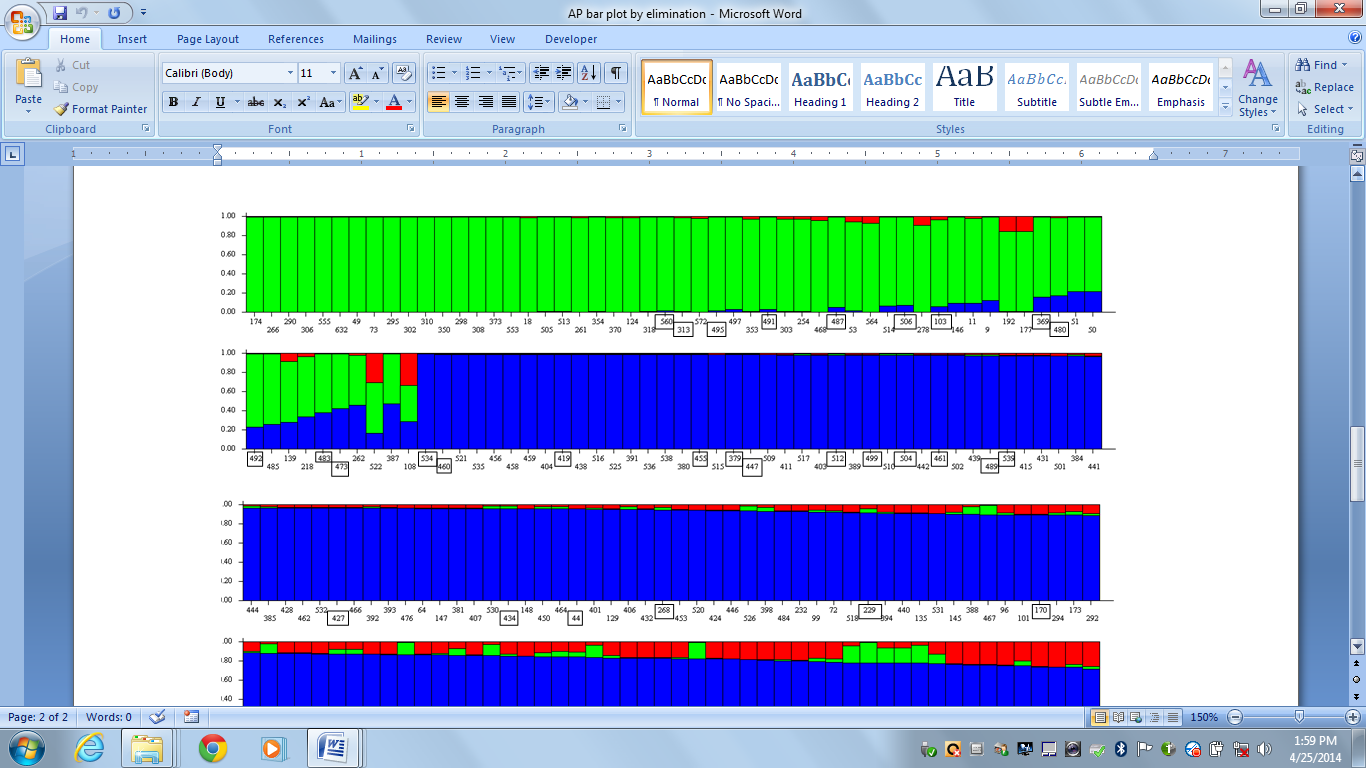


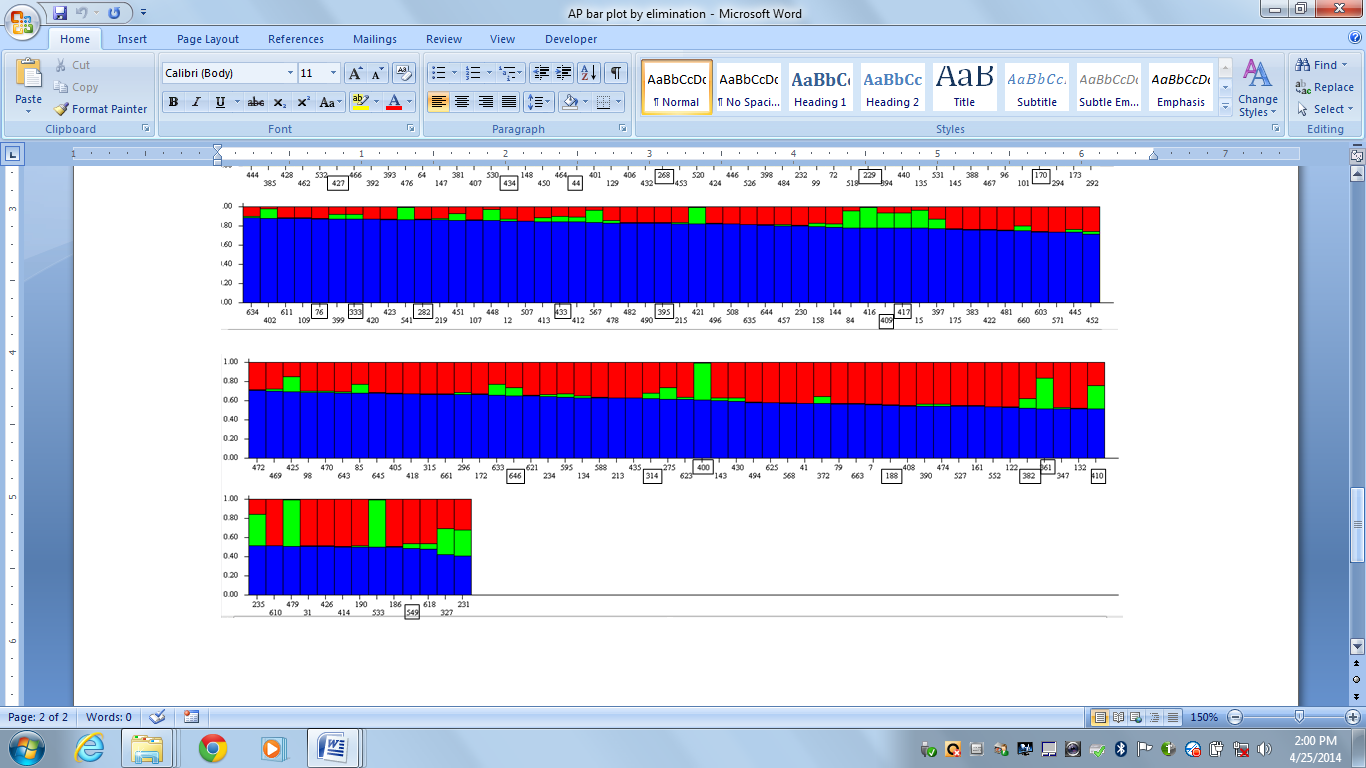


**Fig S3b Model based clustering of Assam samples**


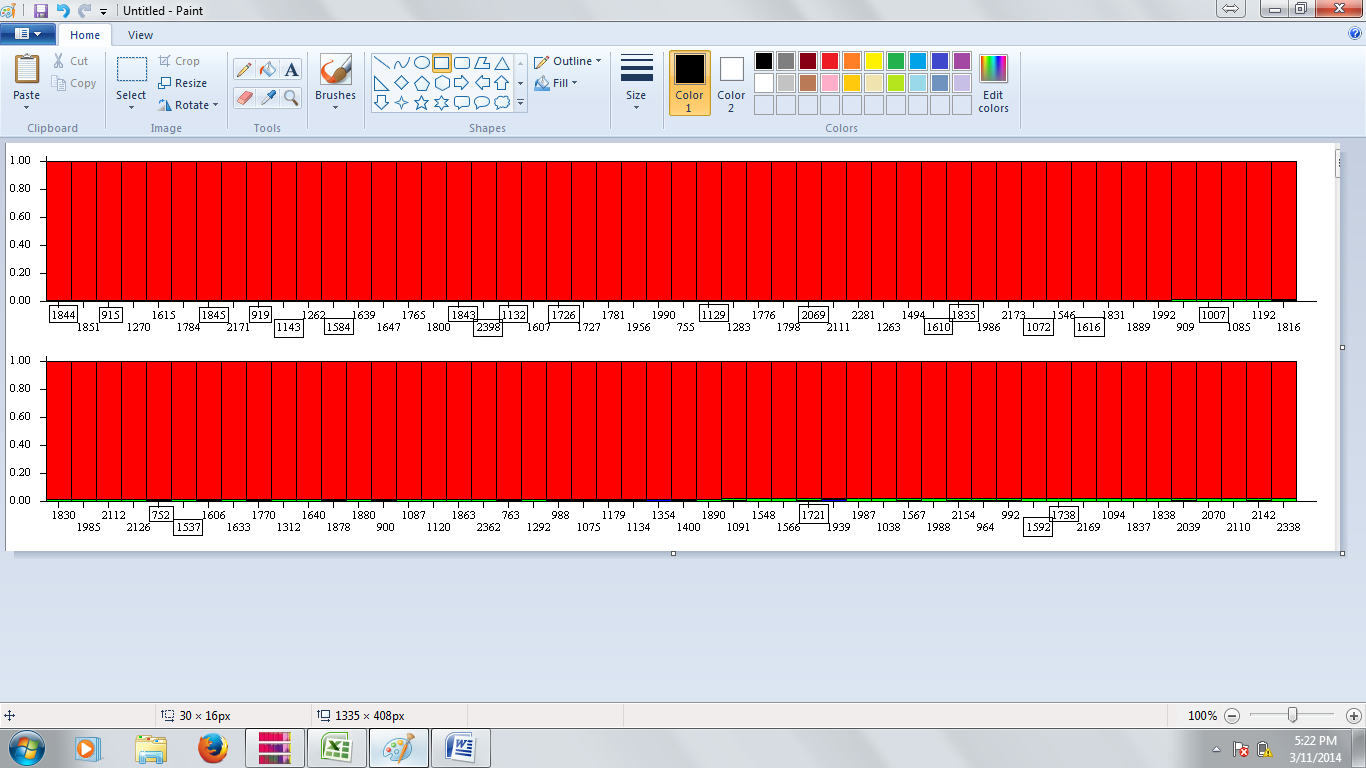


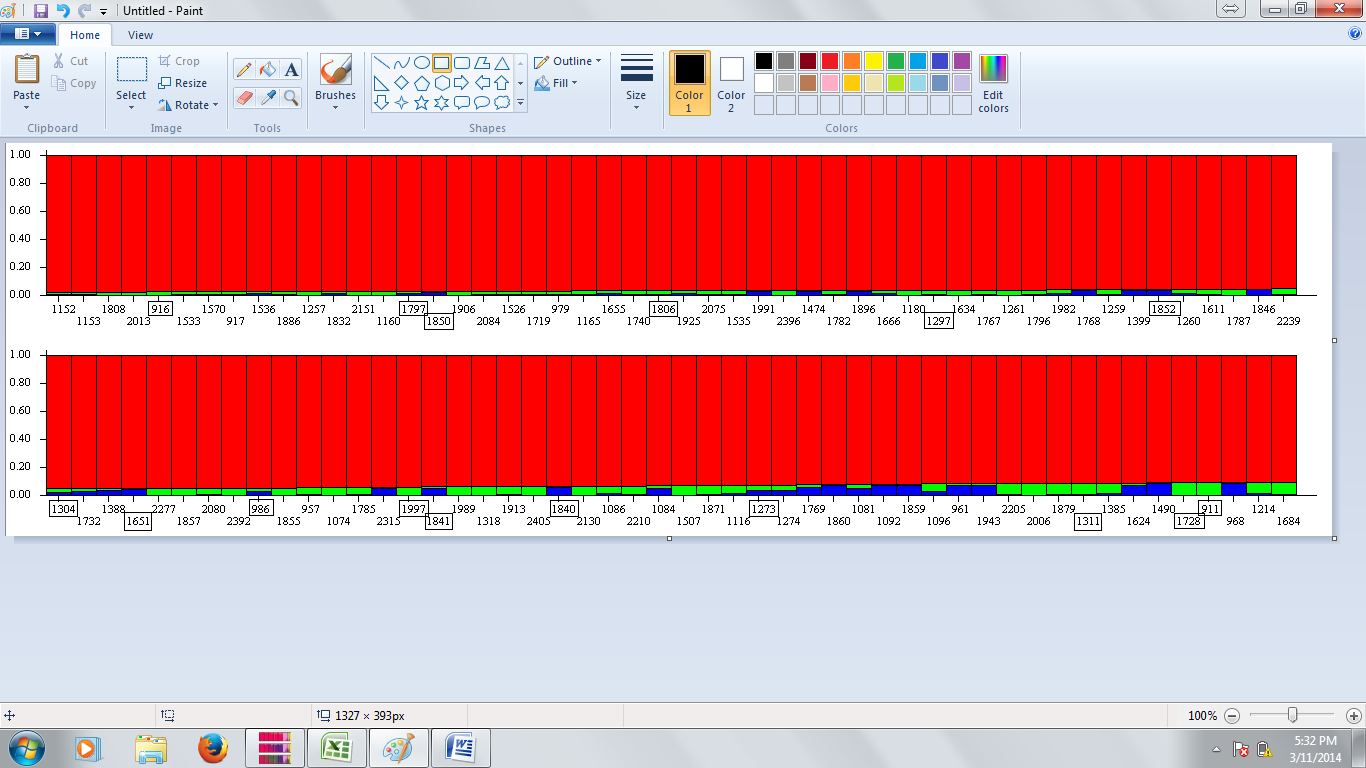


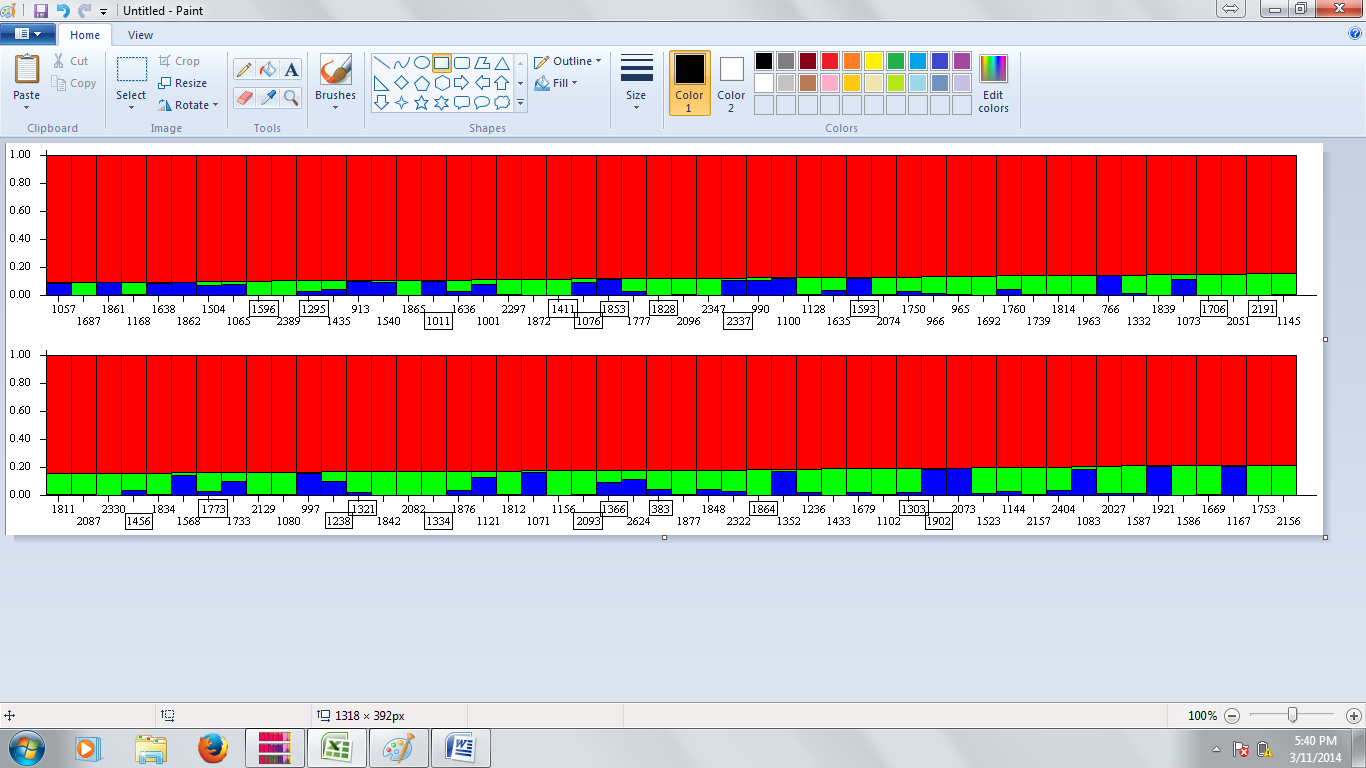


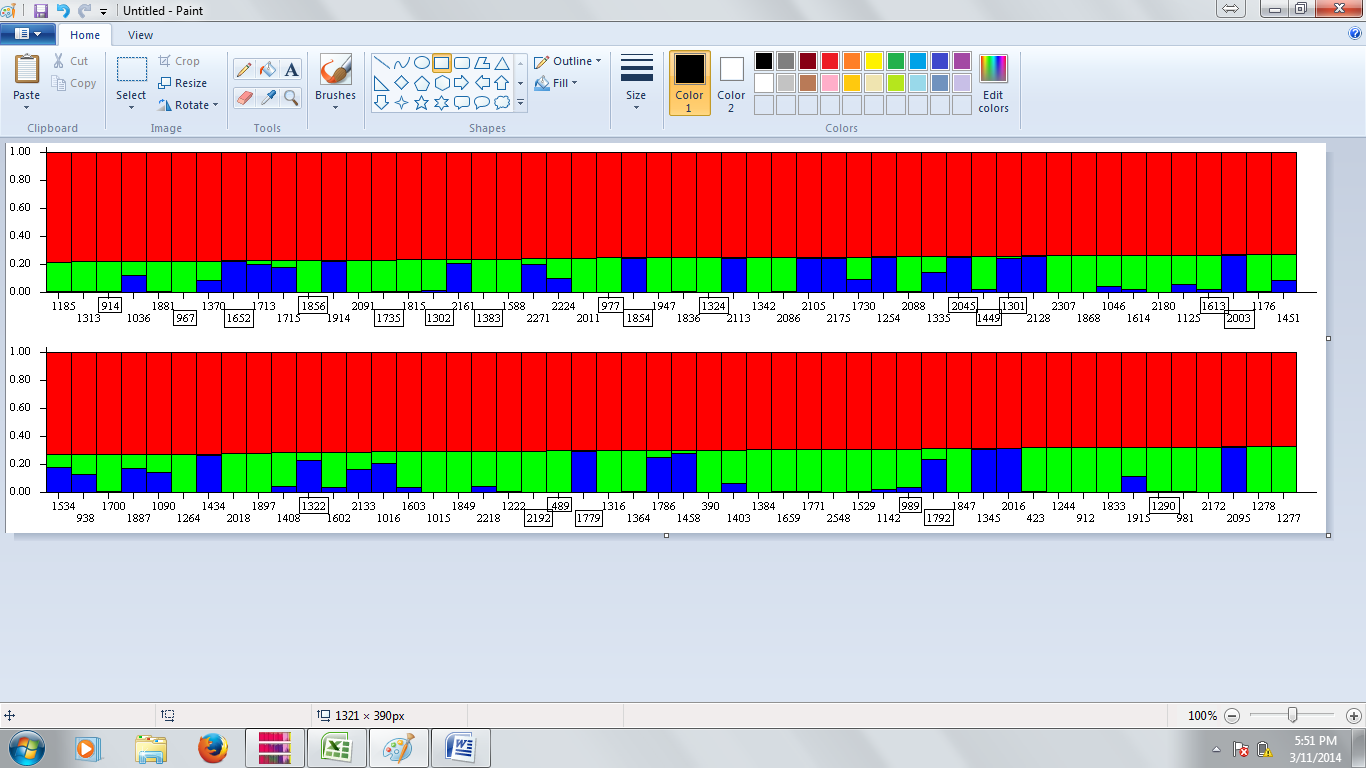


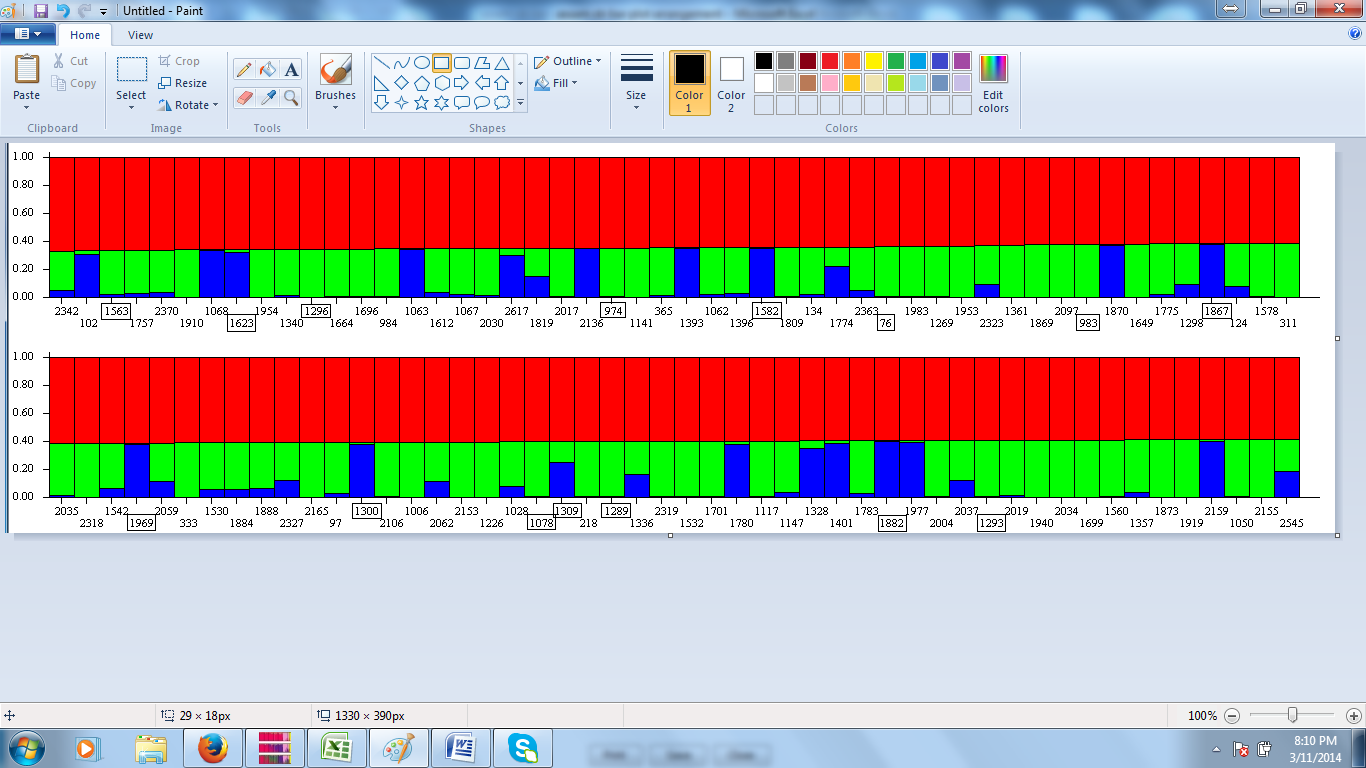


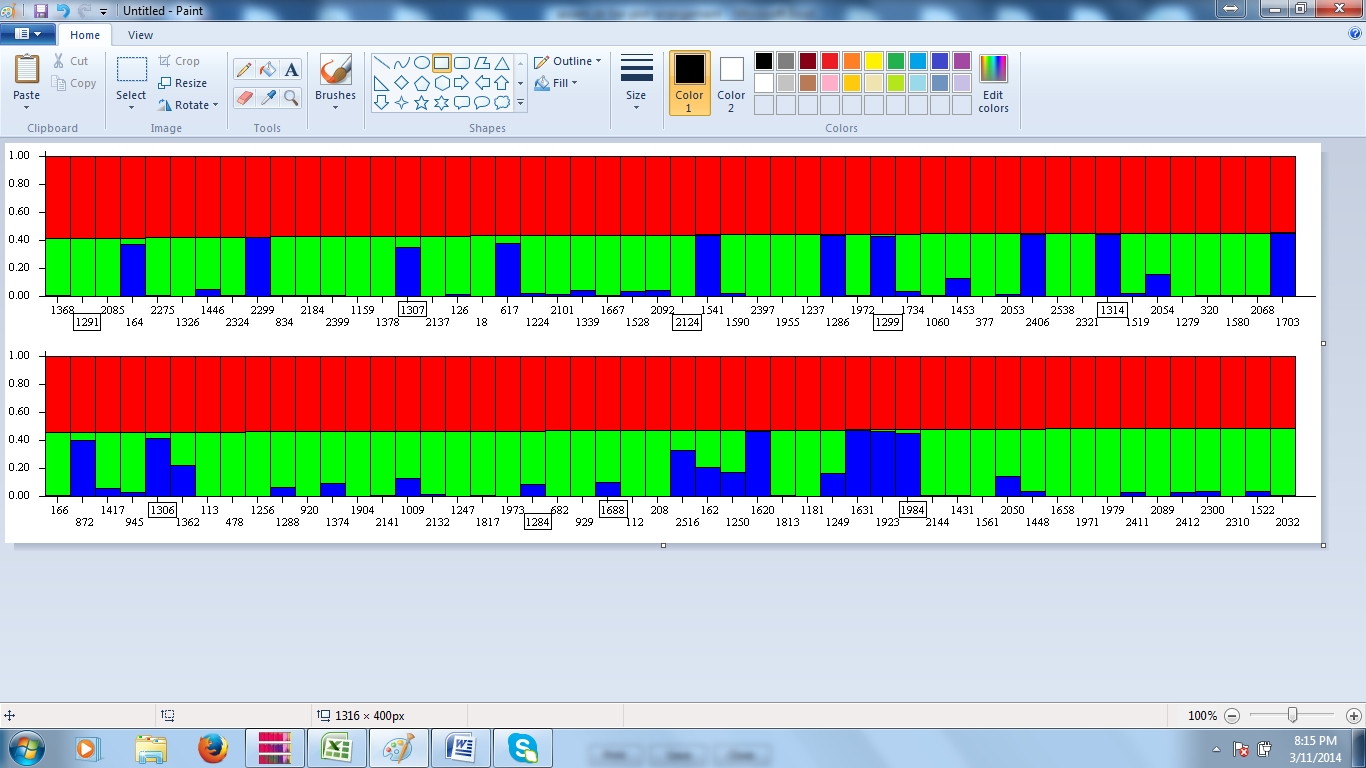


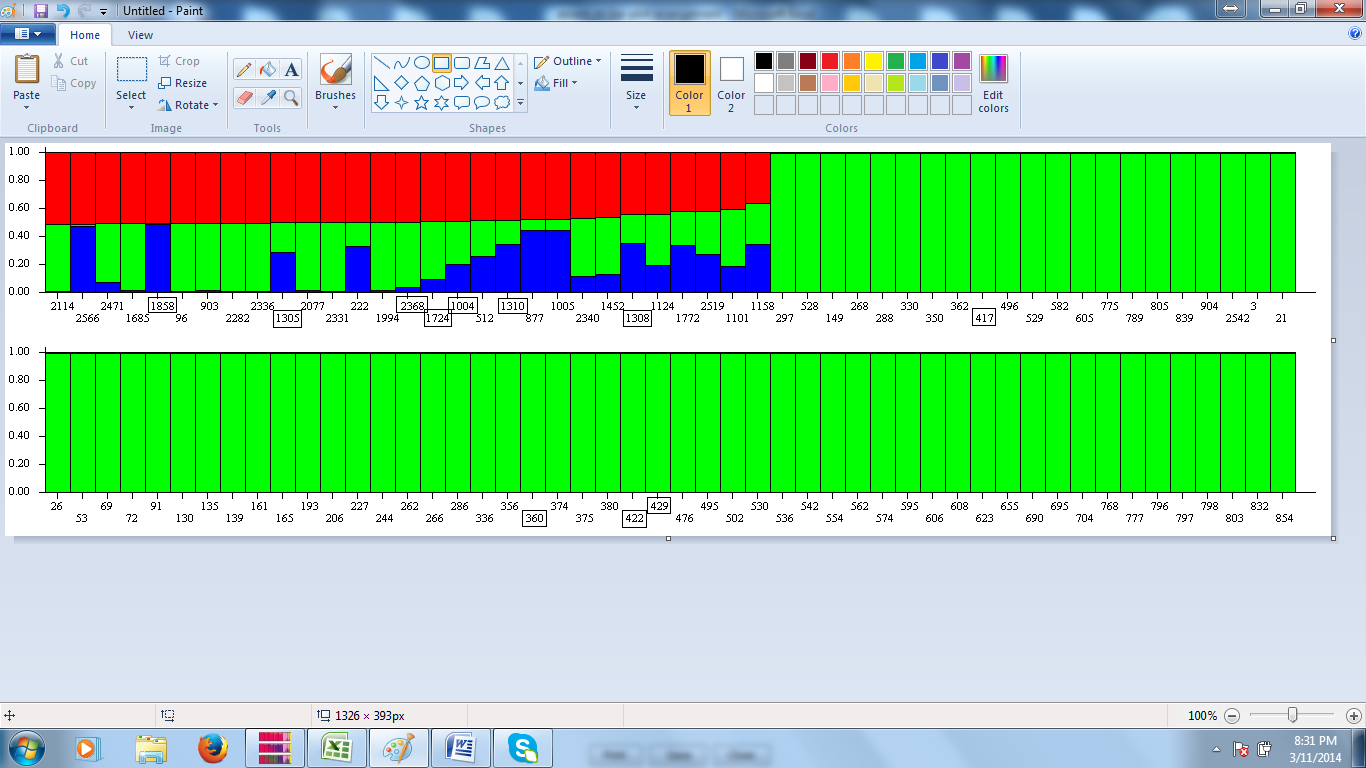


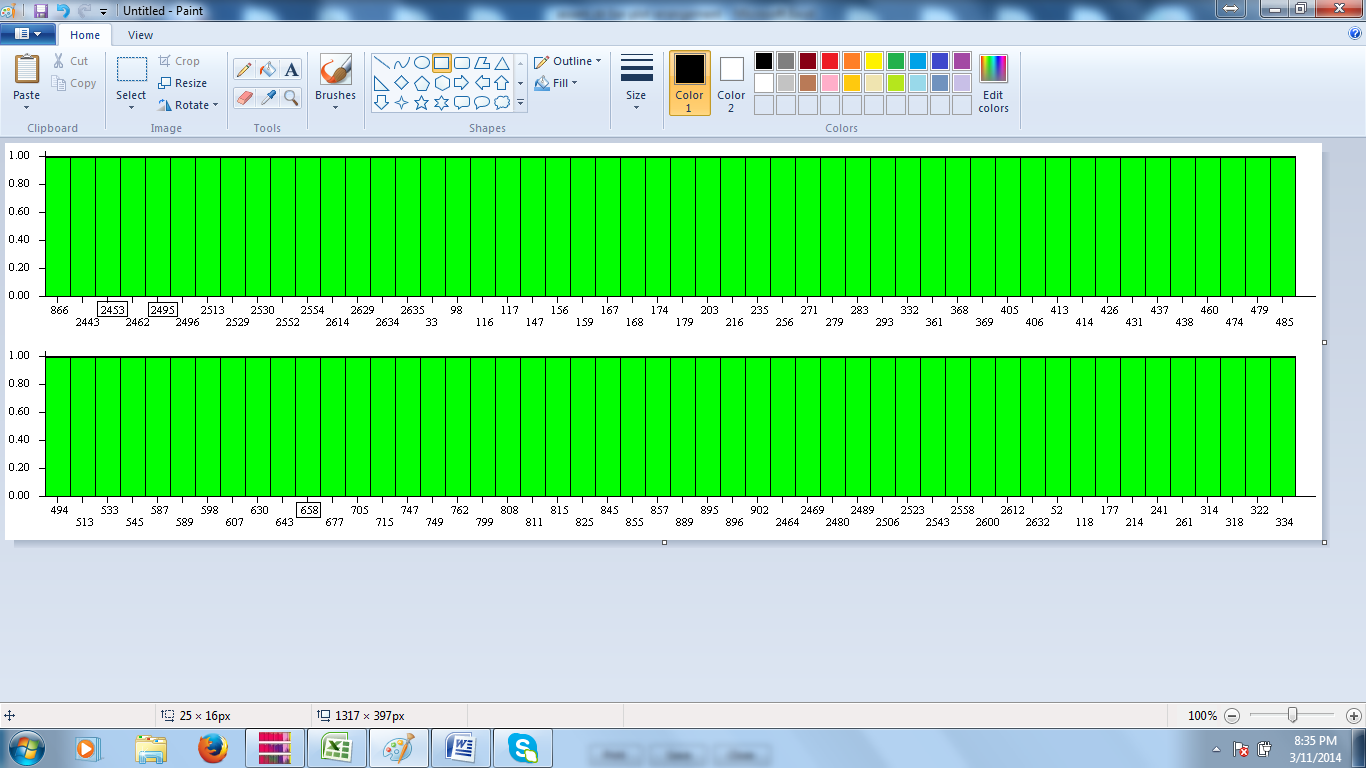


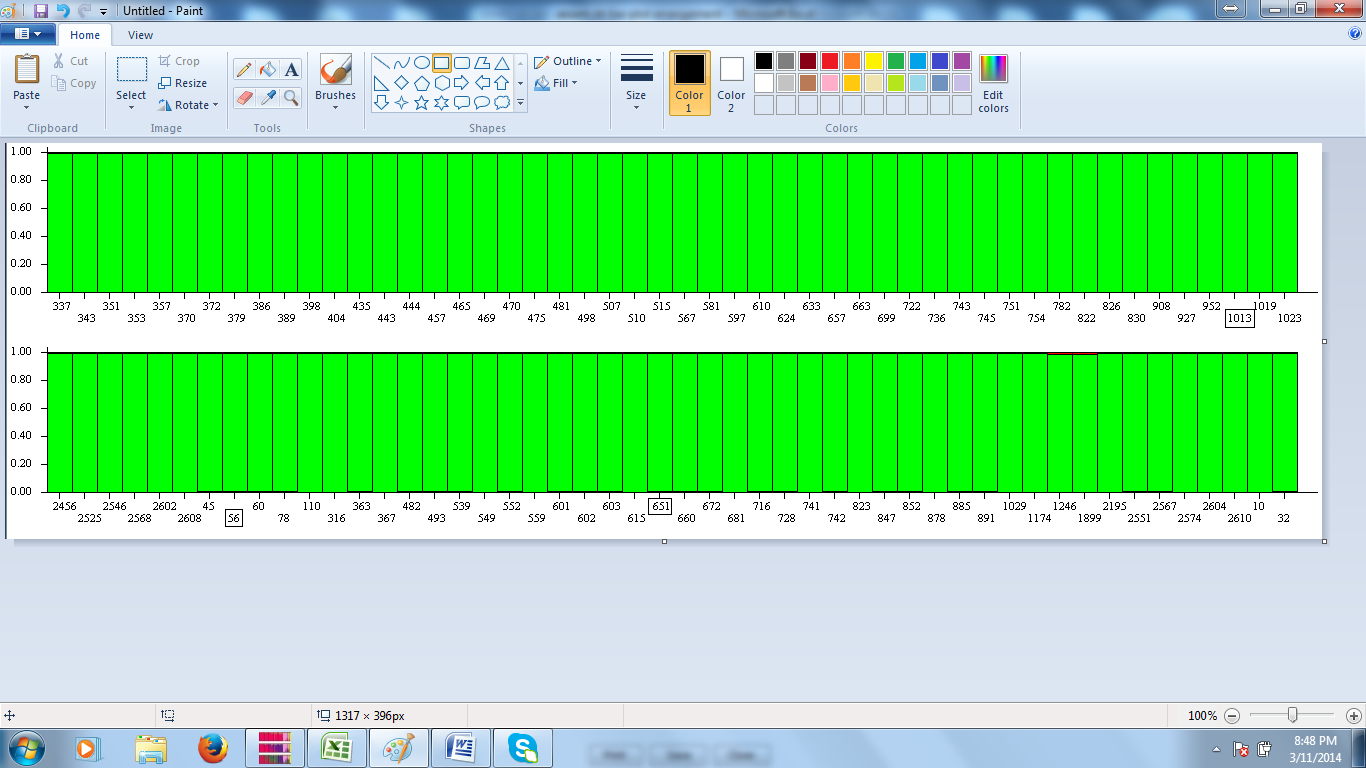


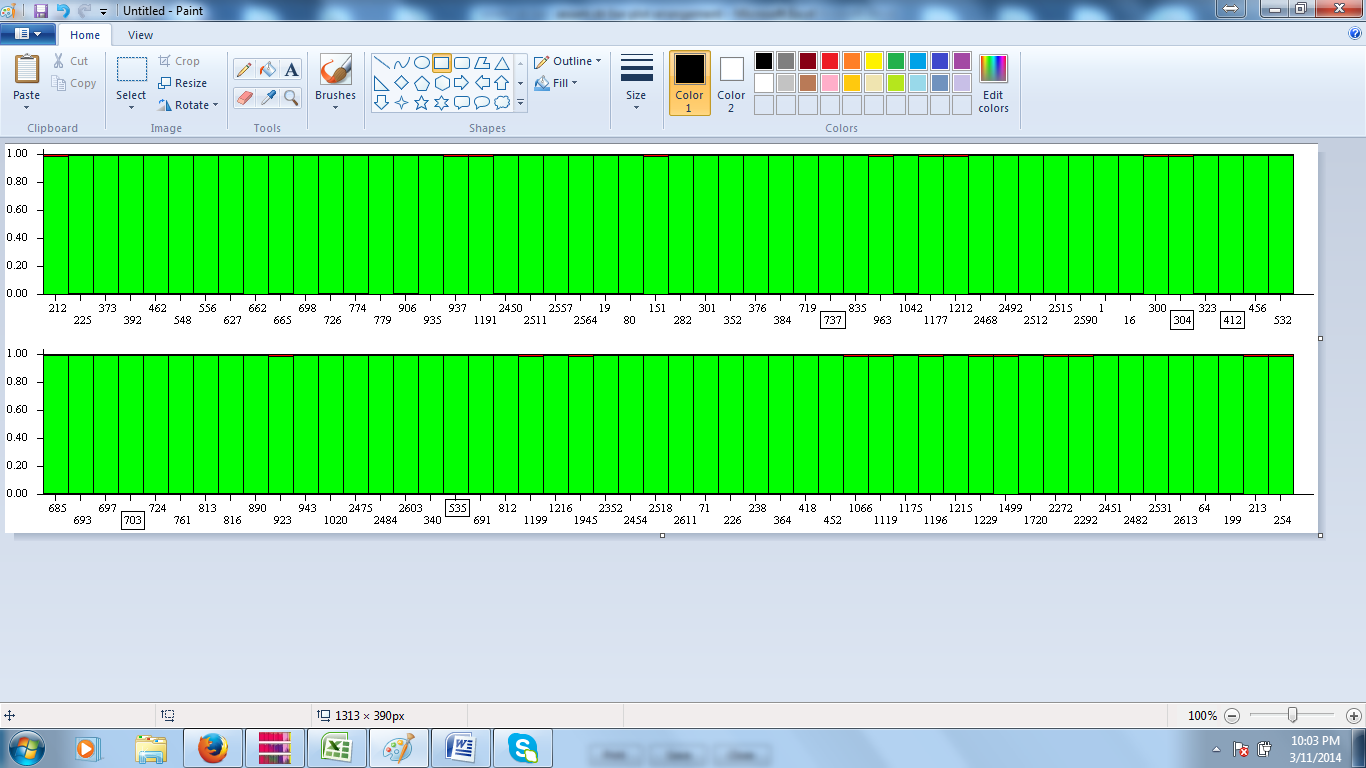


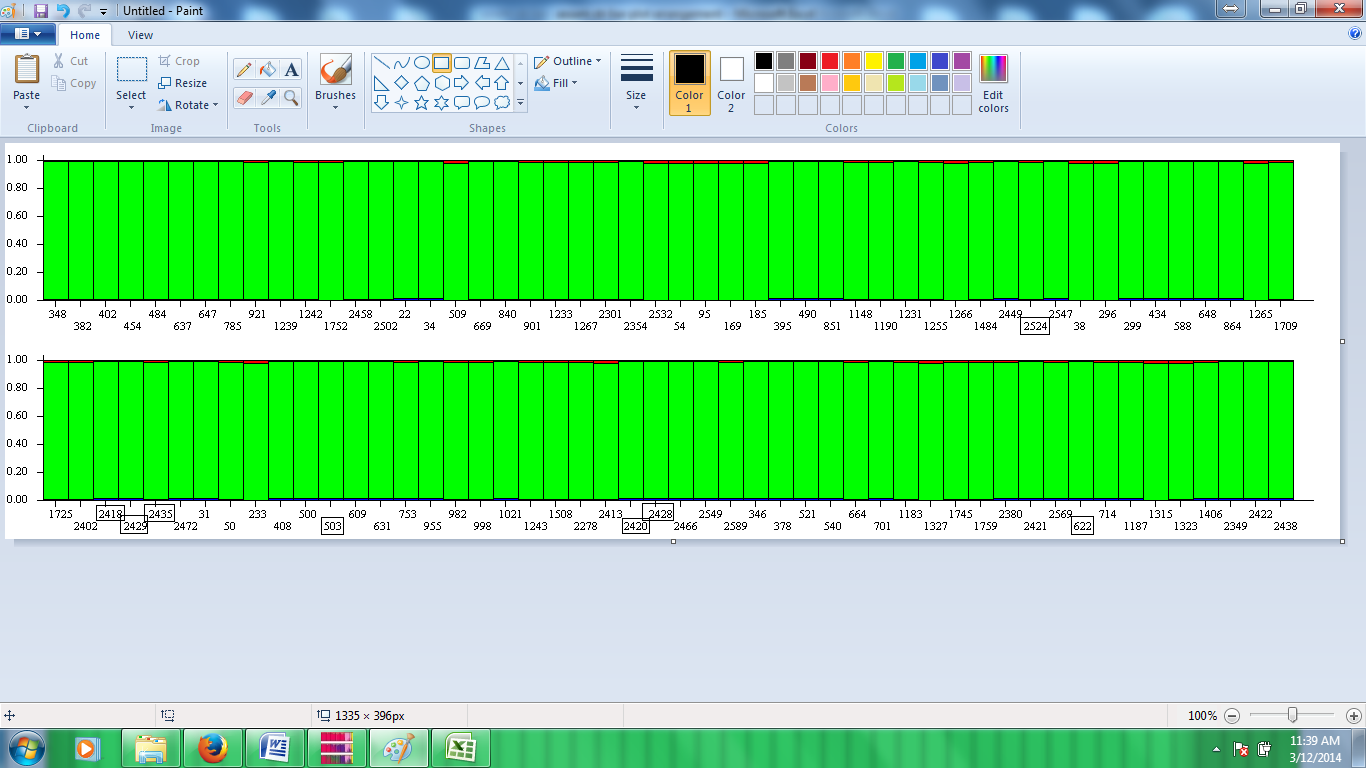


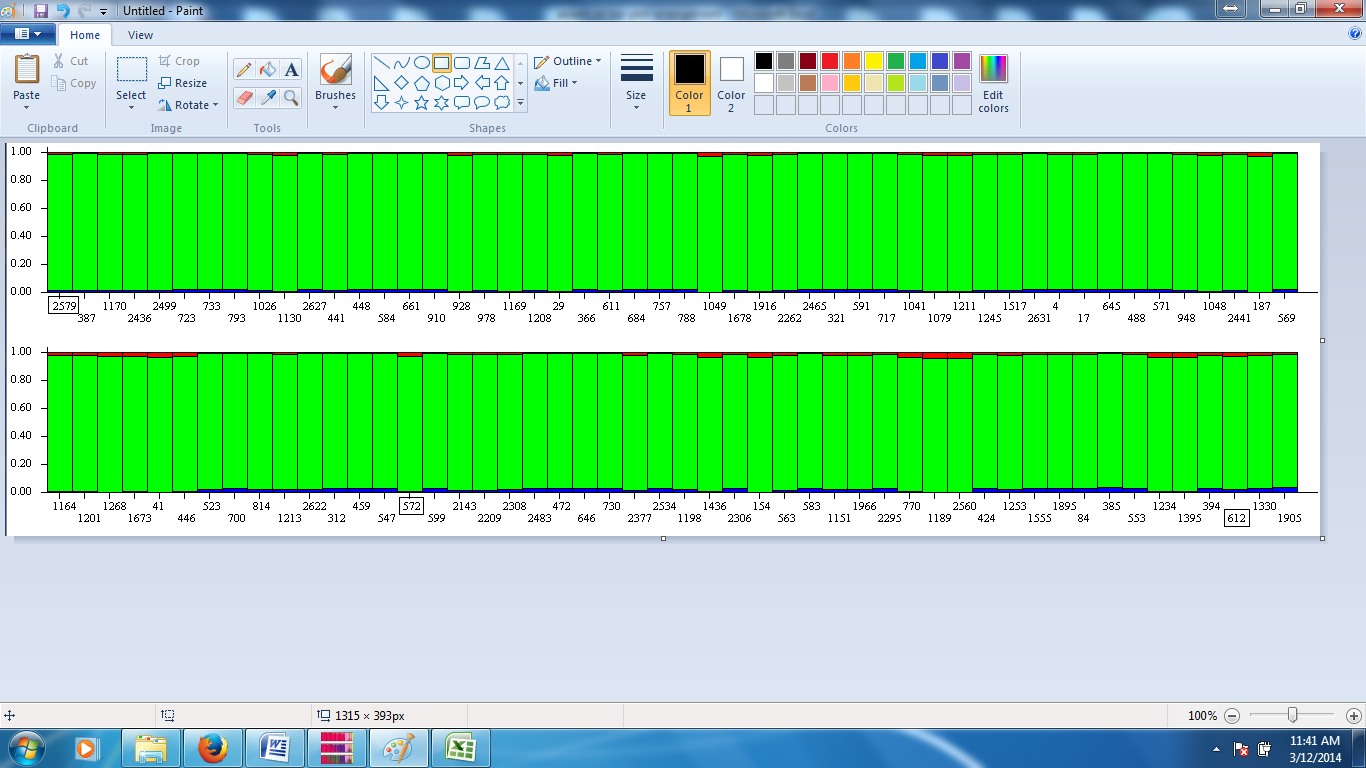


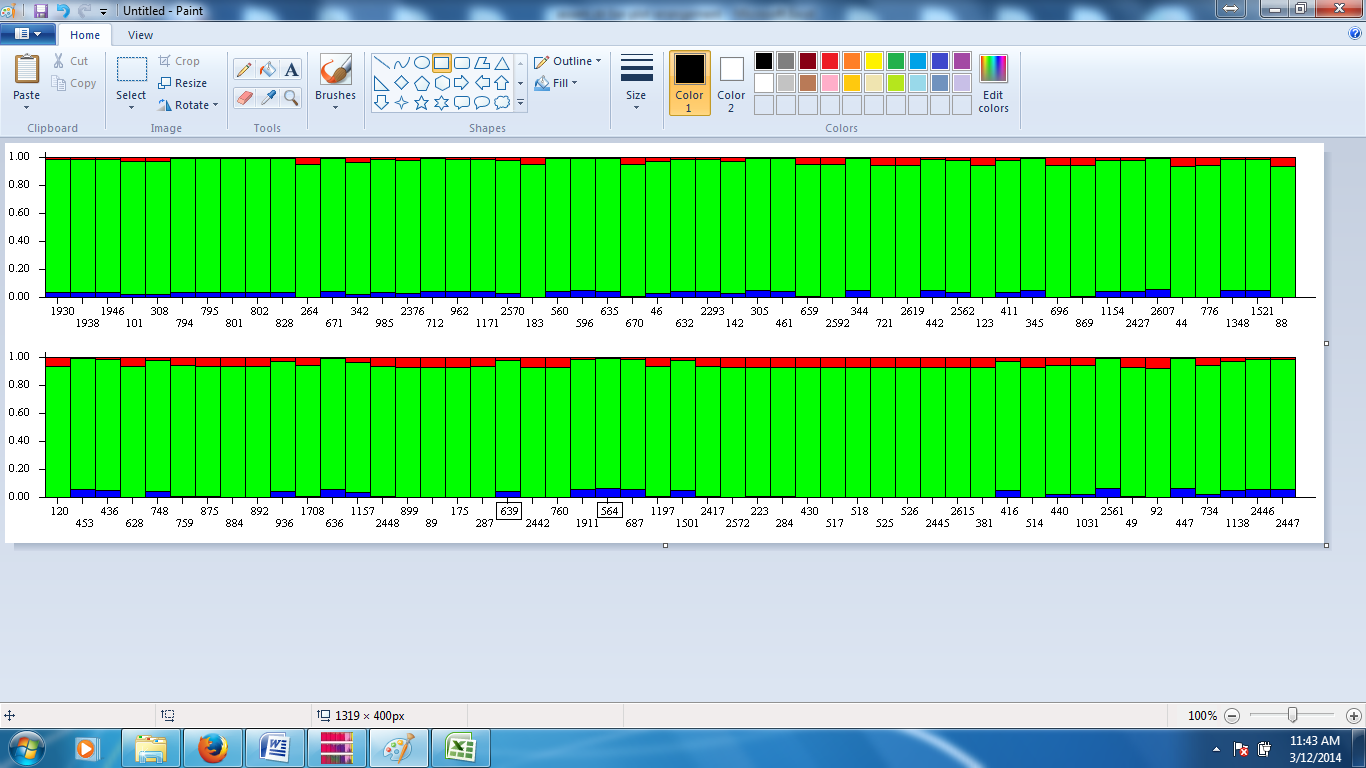


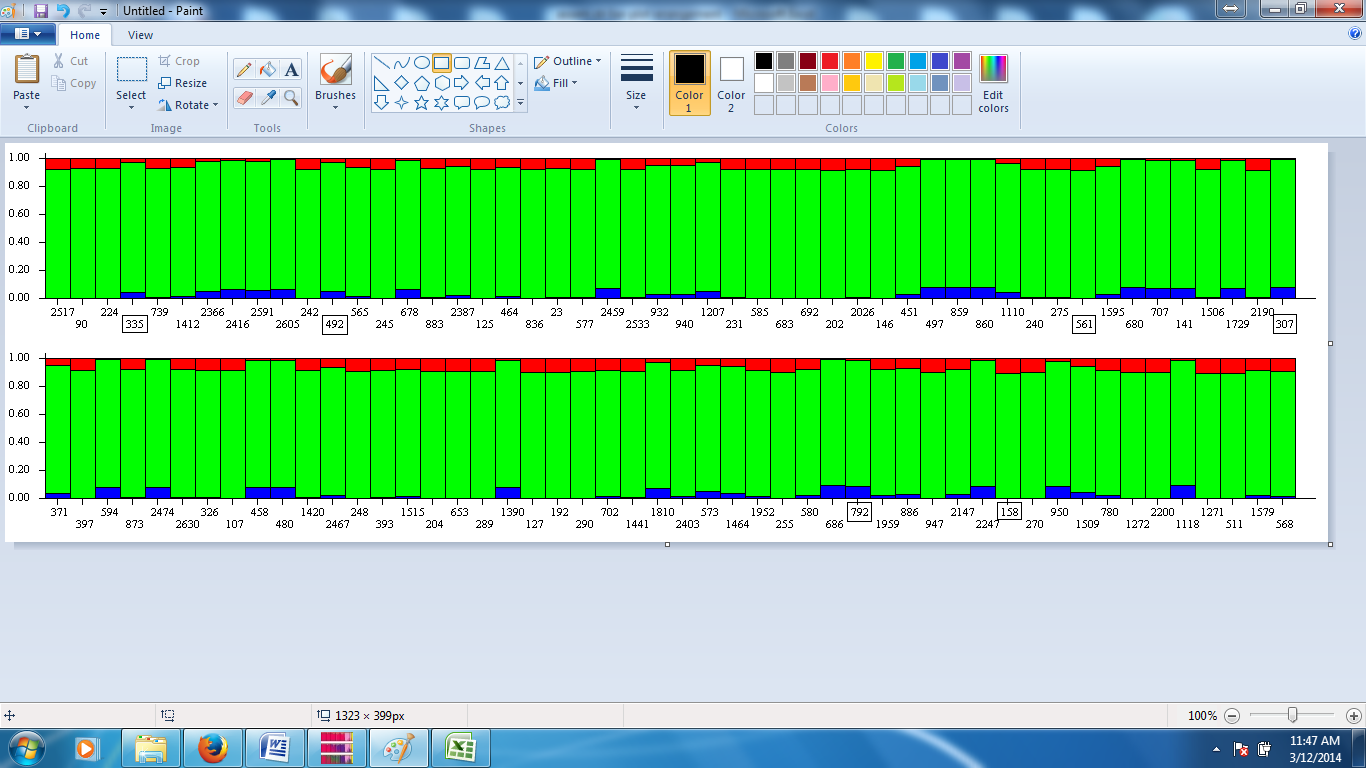


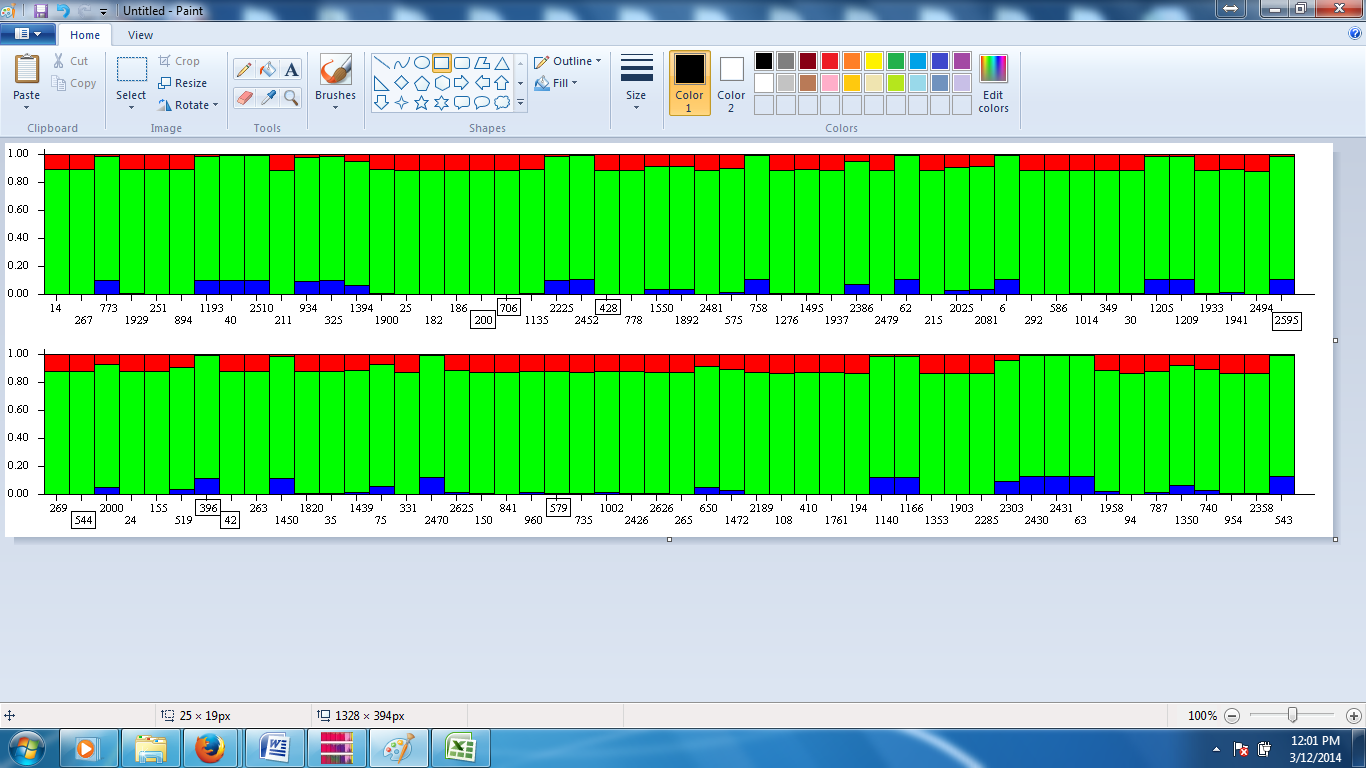


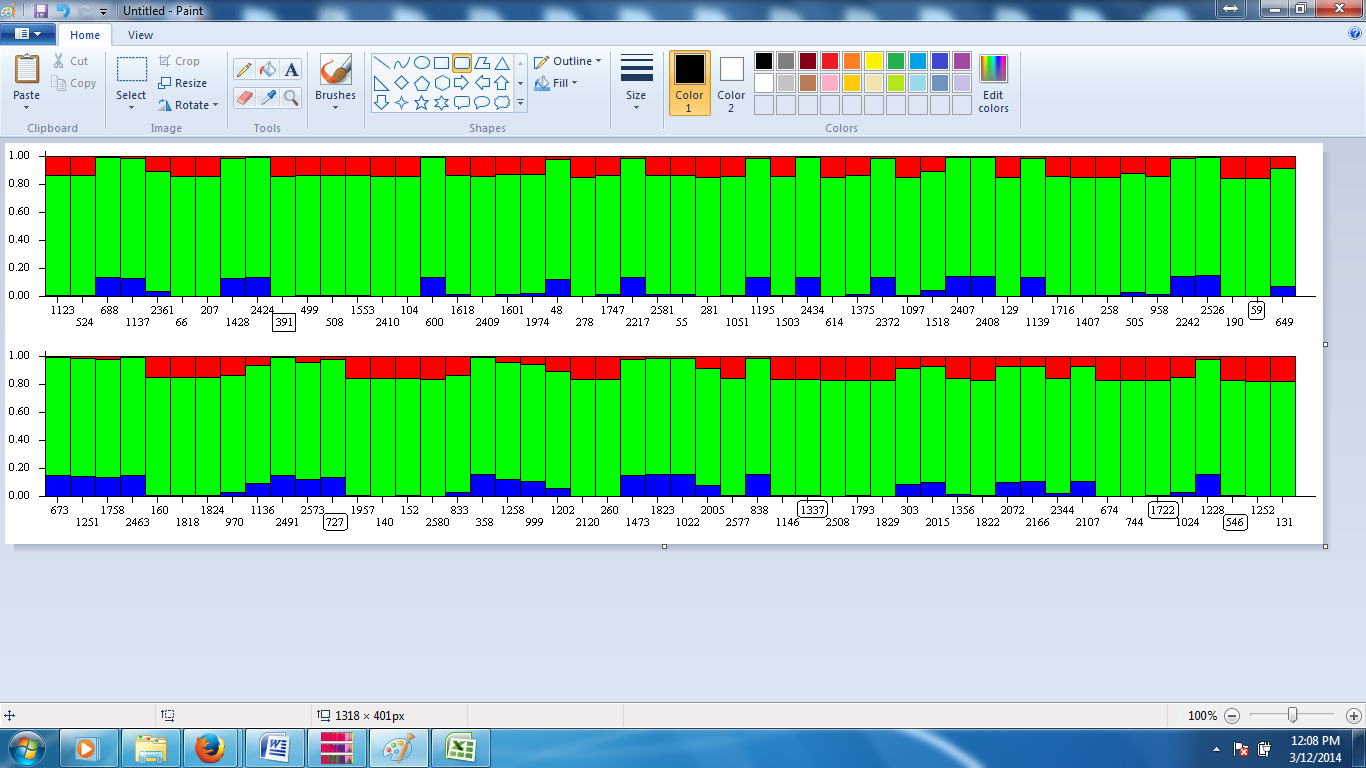


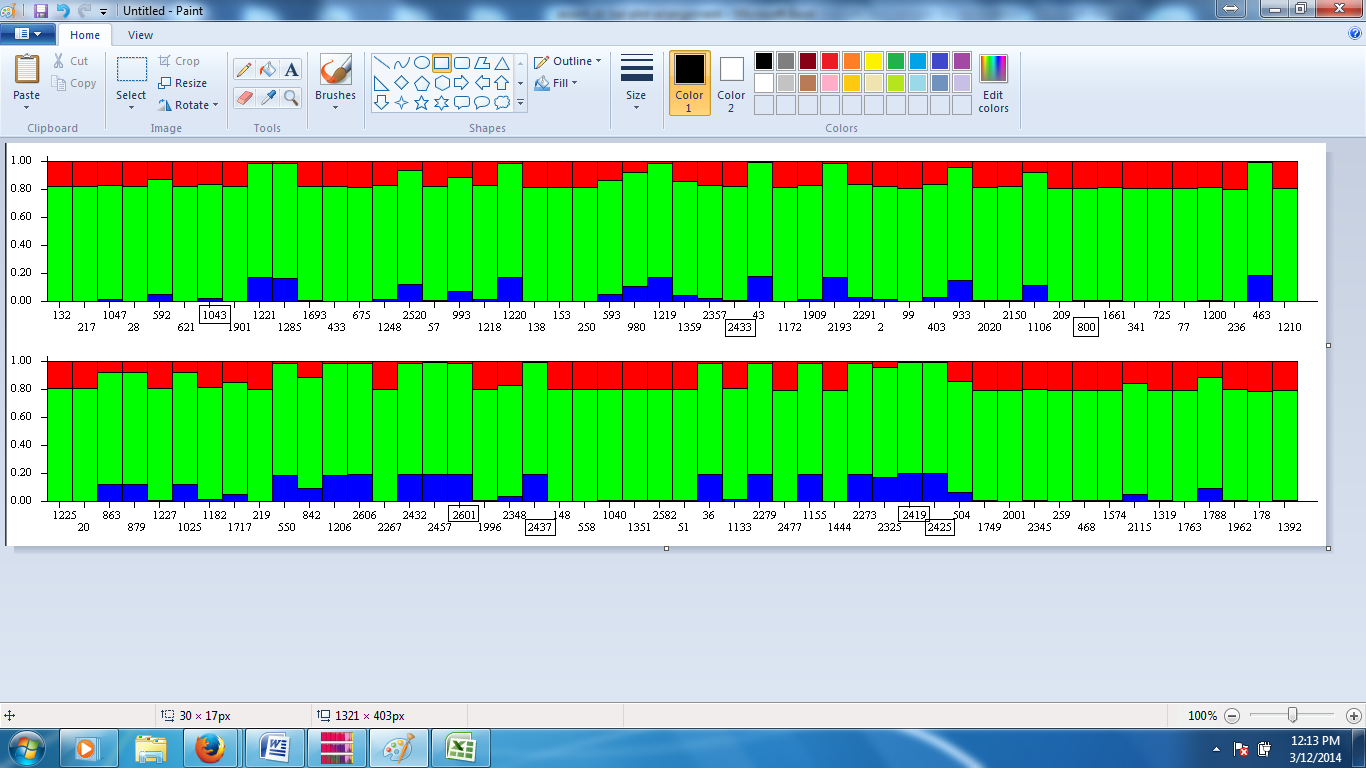


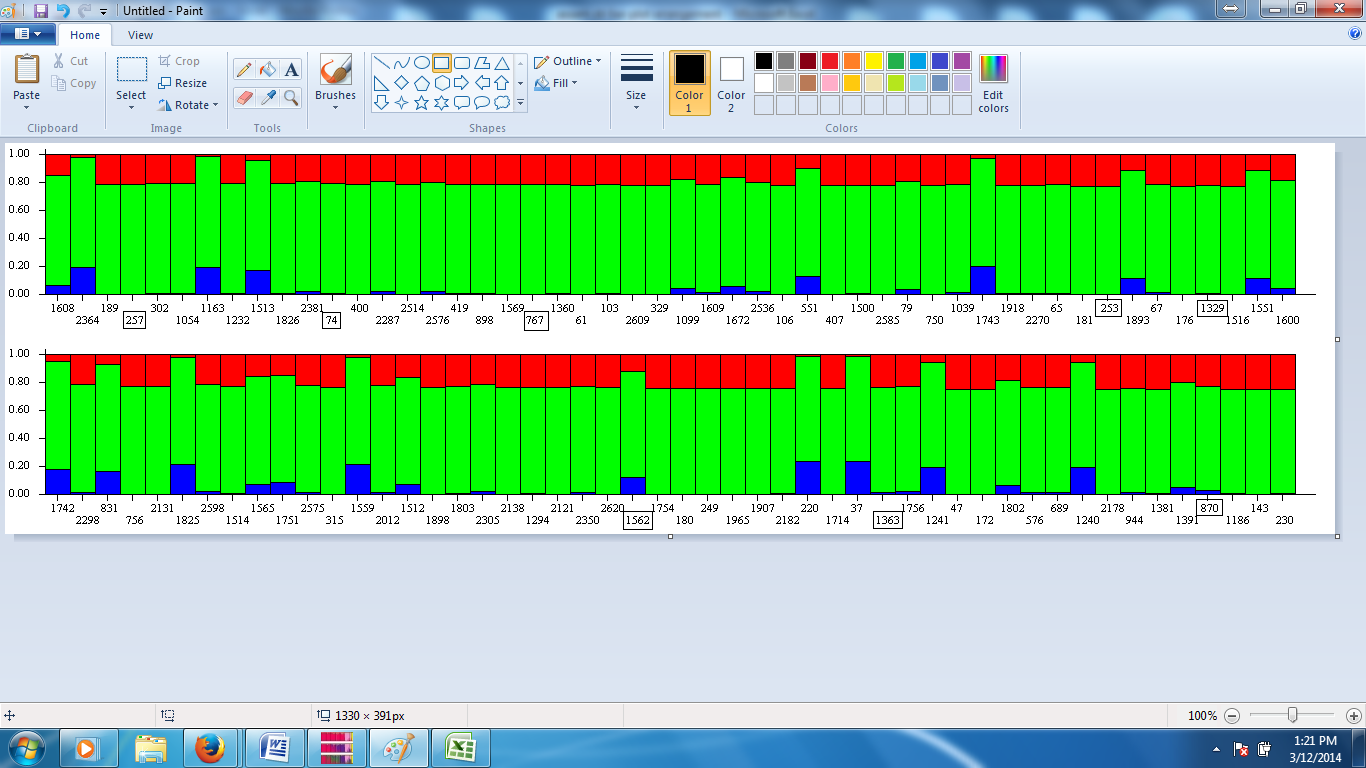


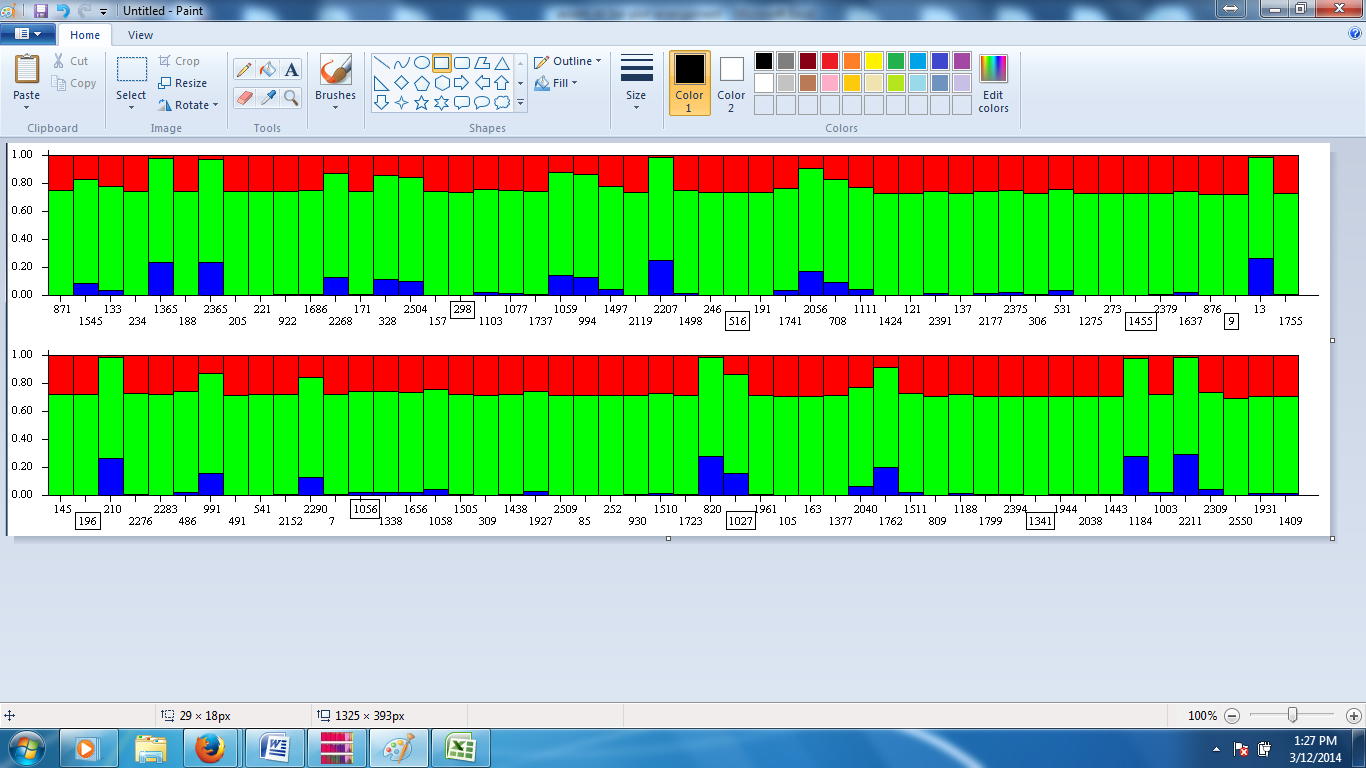


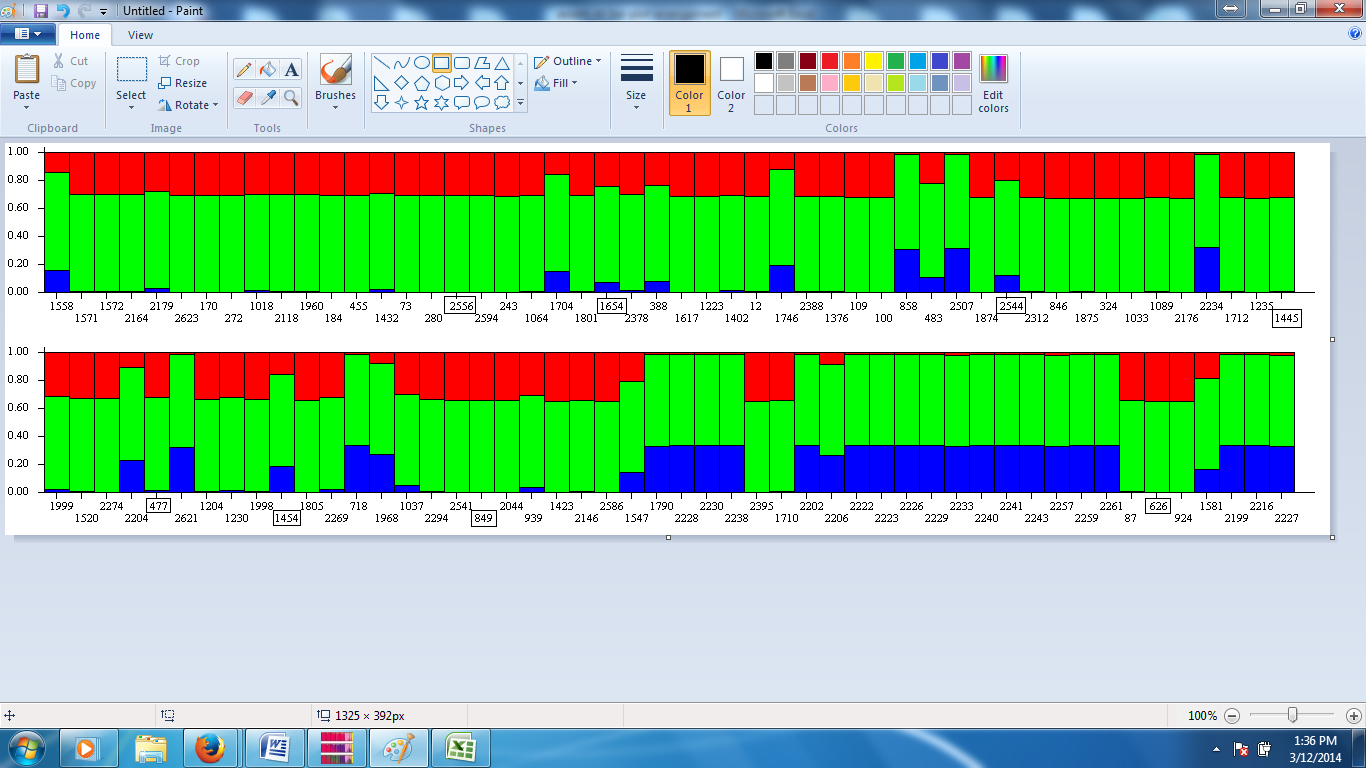


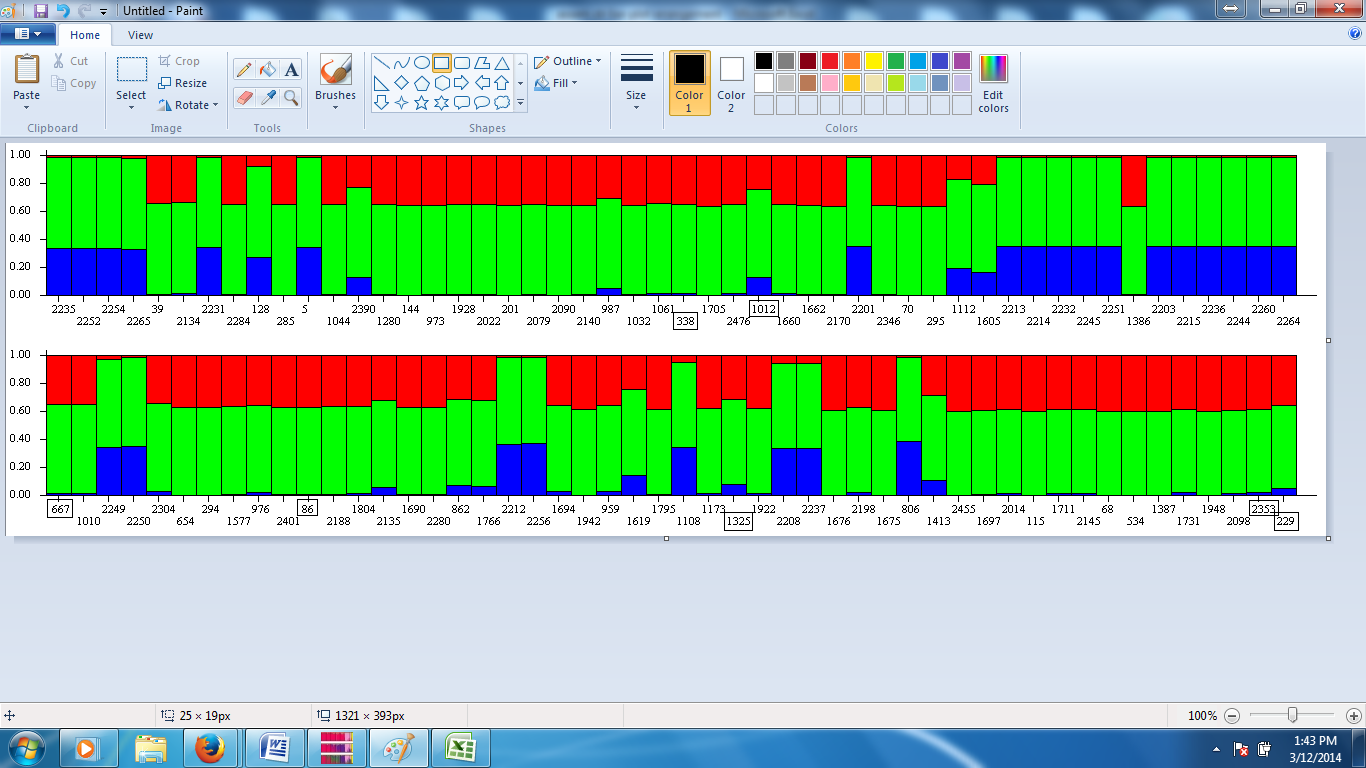


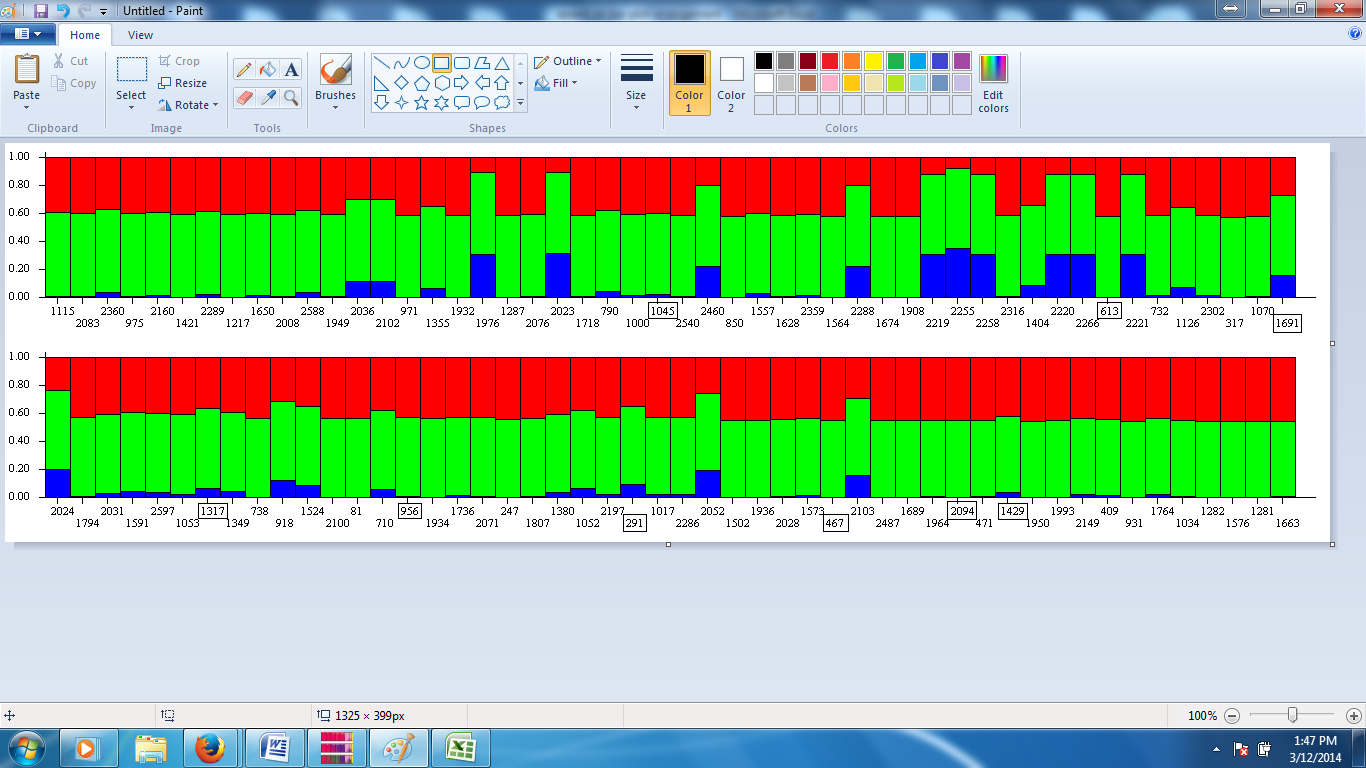


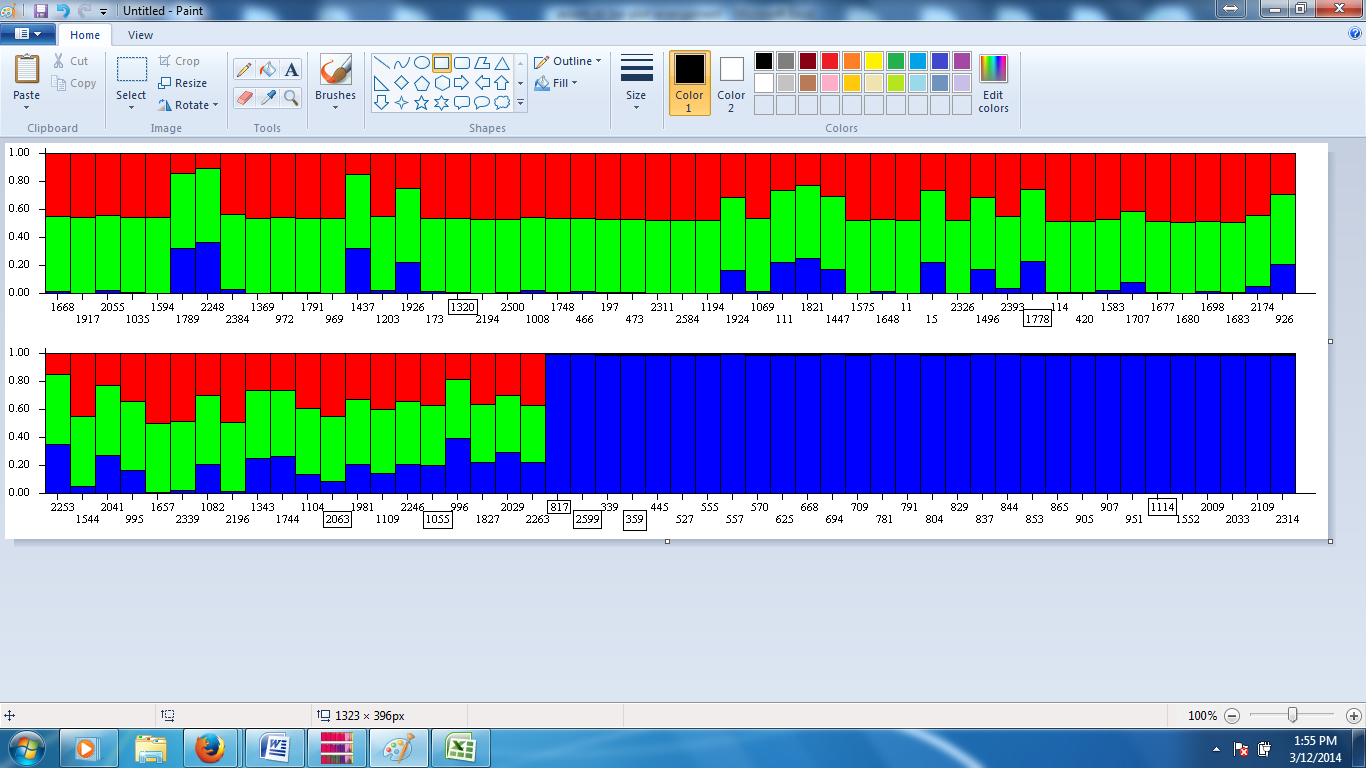


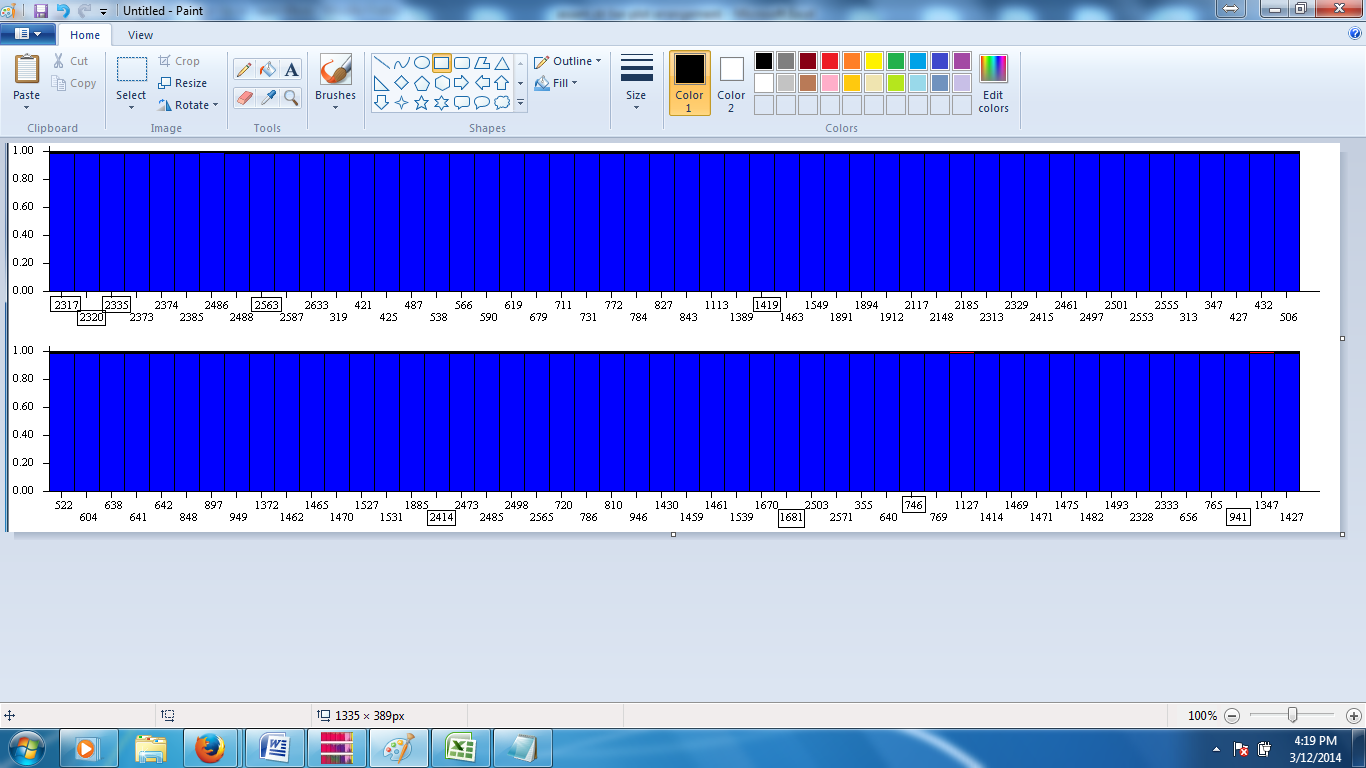


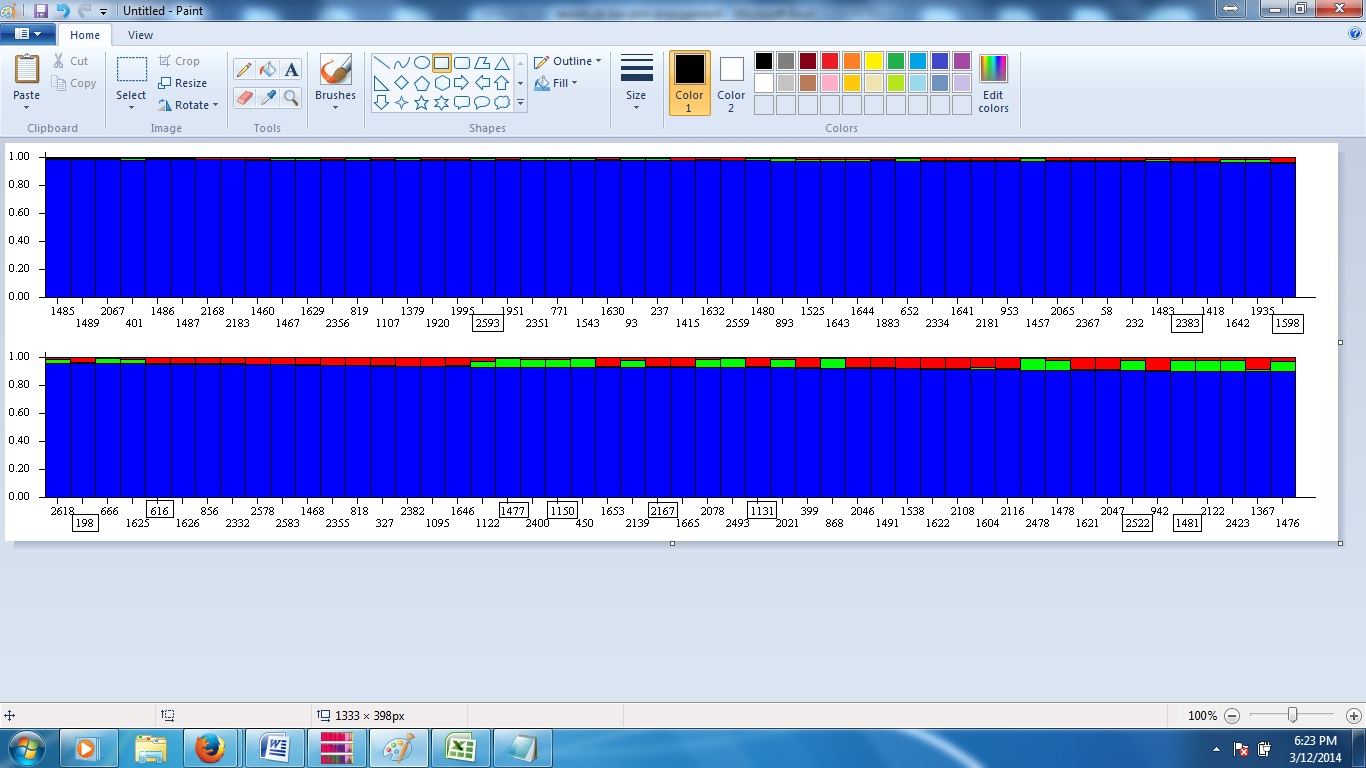


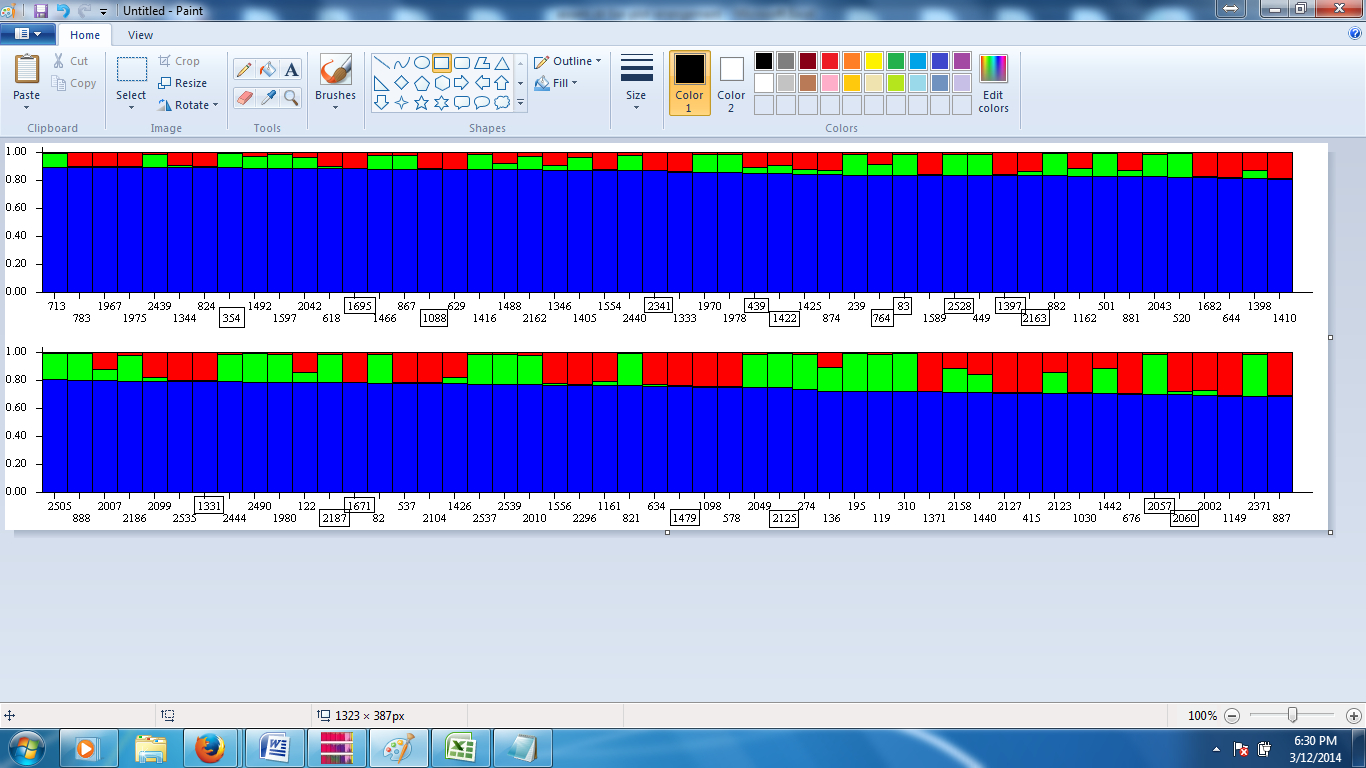


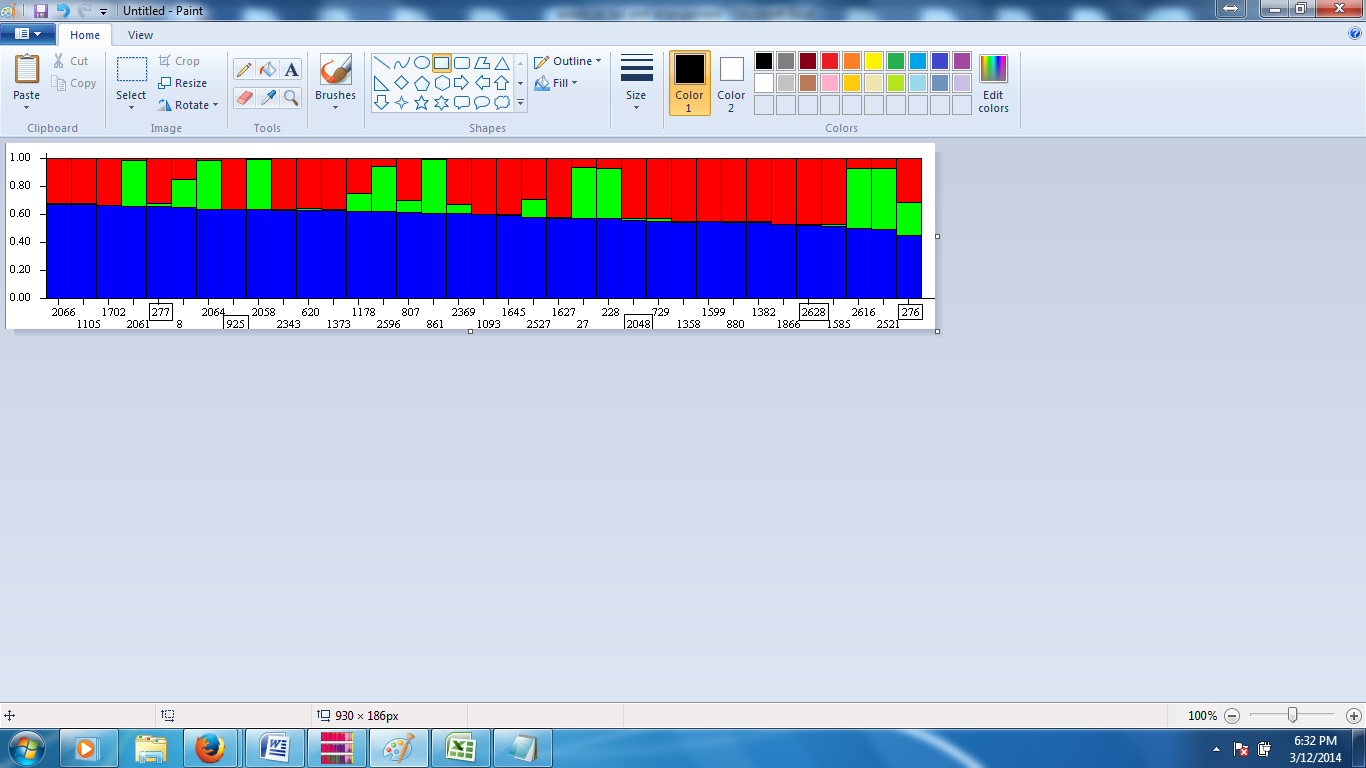


**Fig S3c Model based clustering of Manipur**


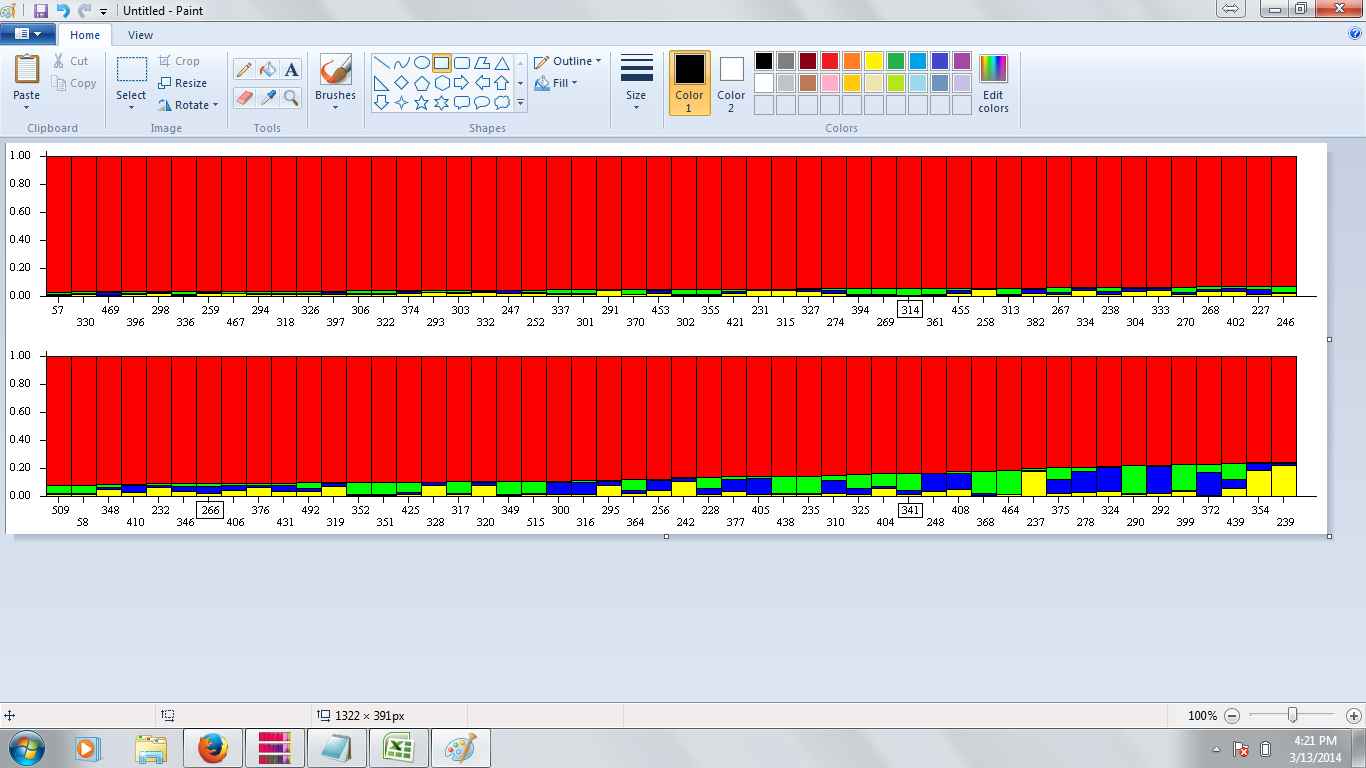


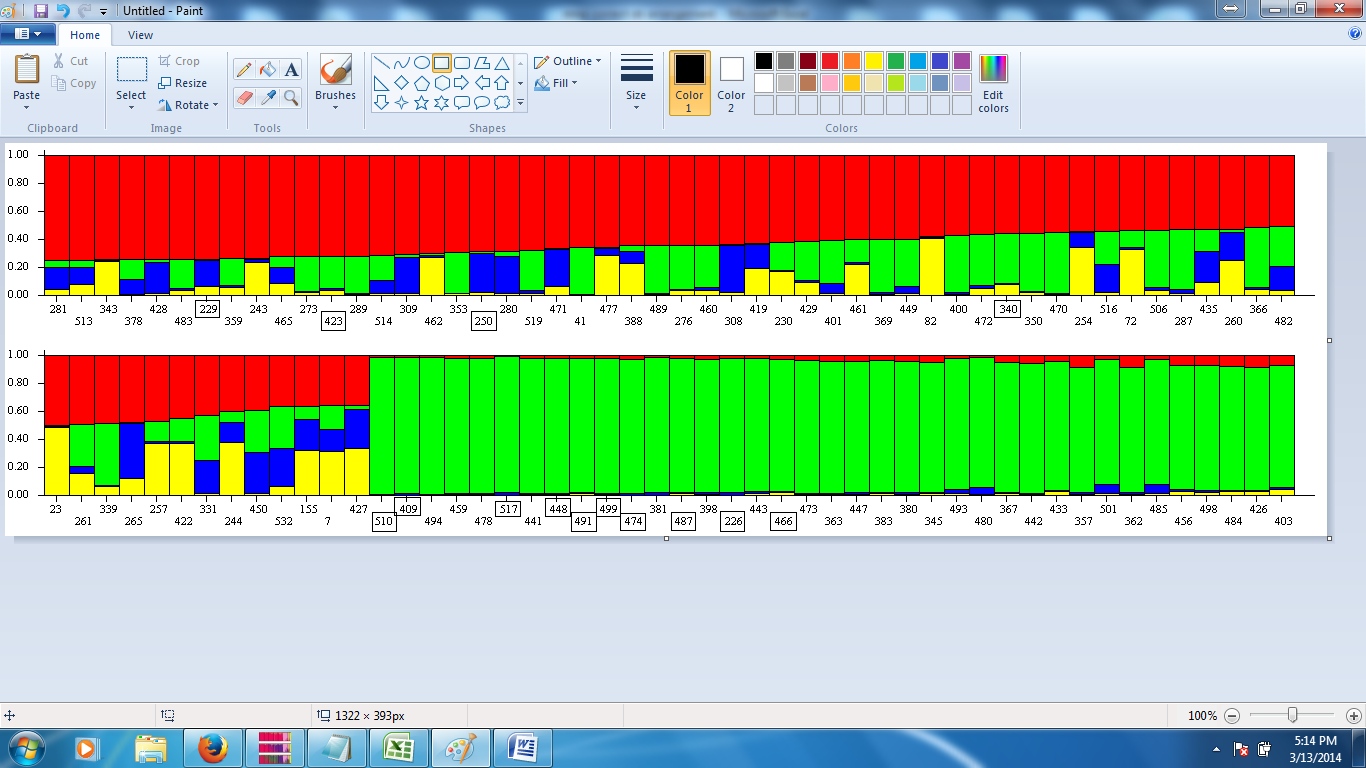


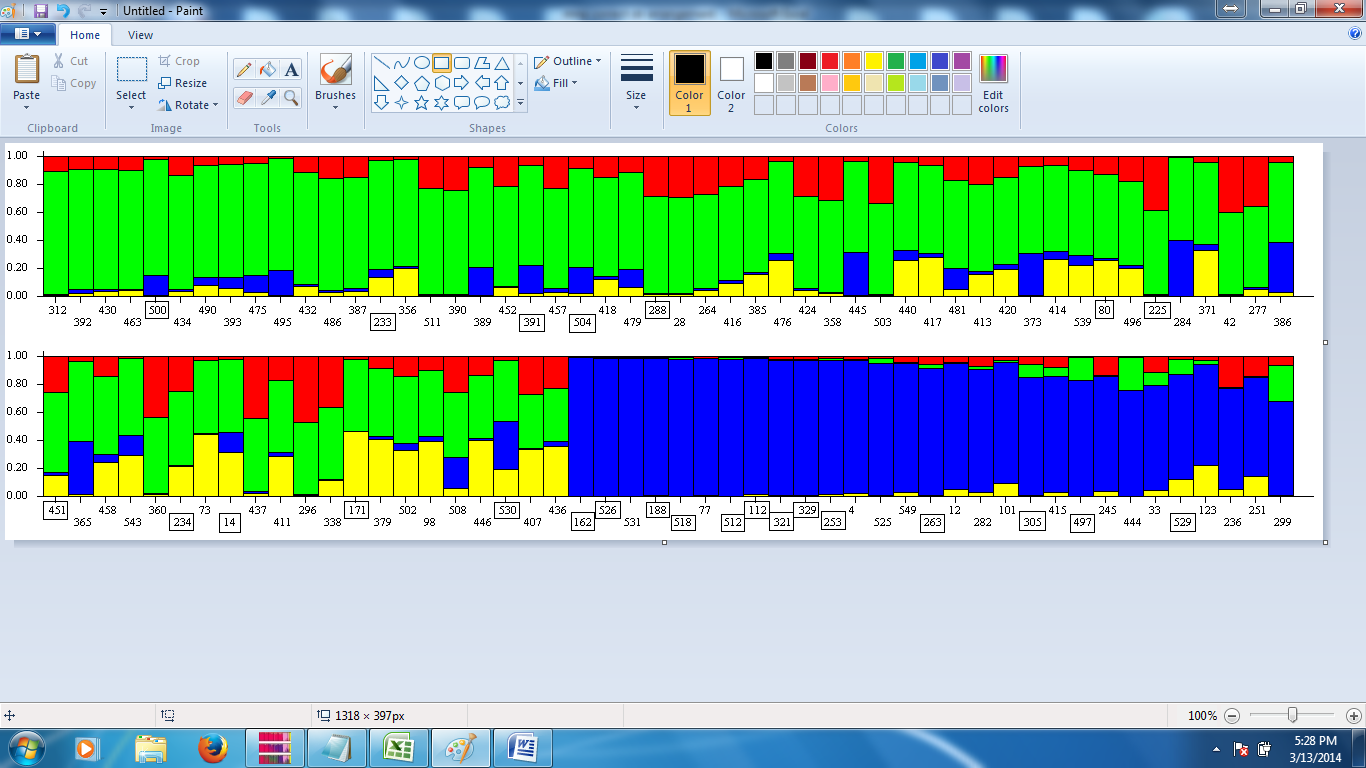


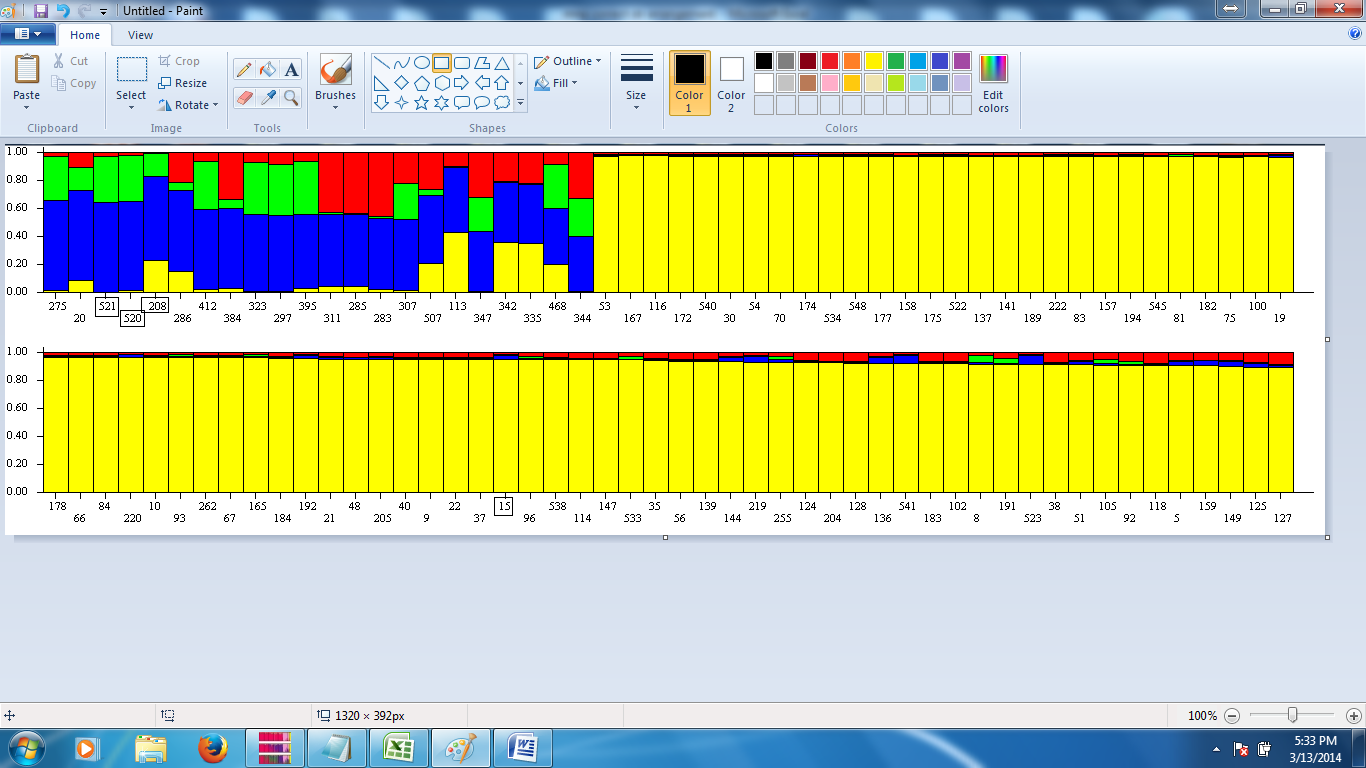


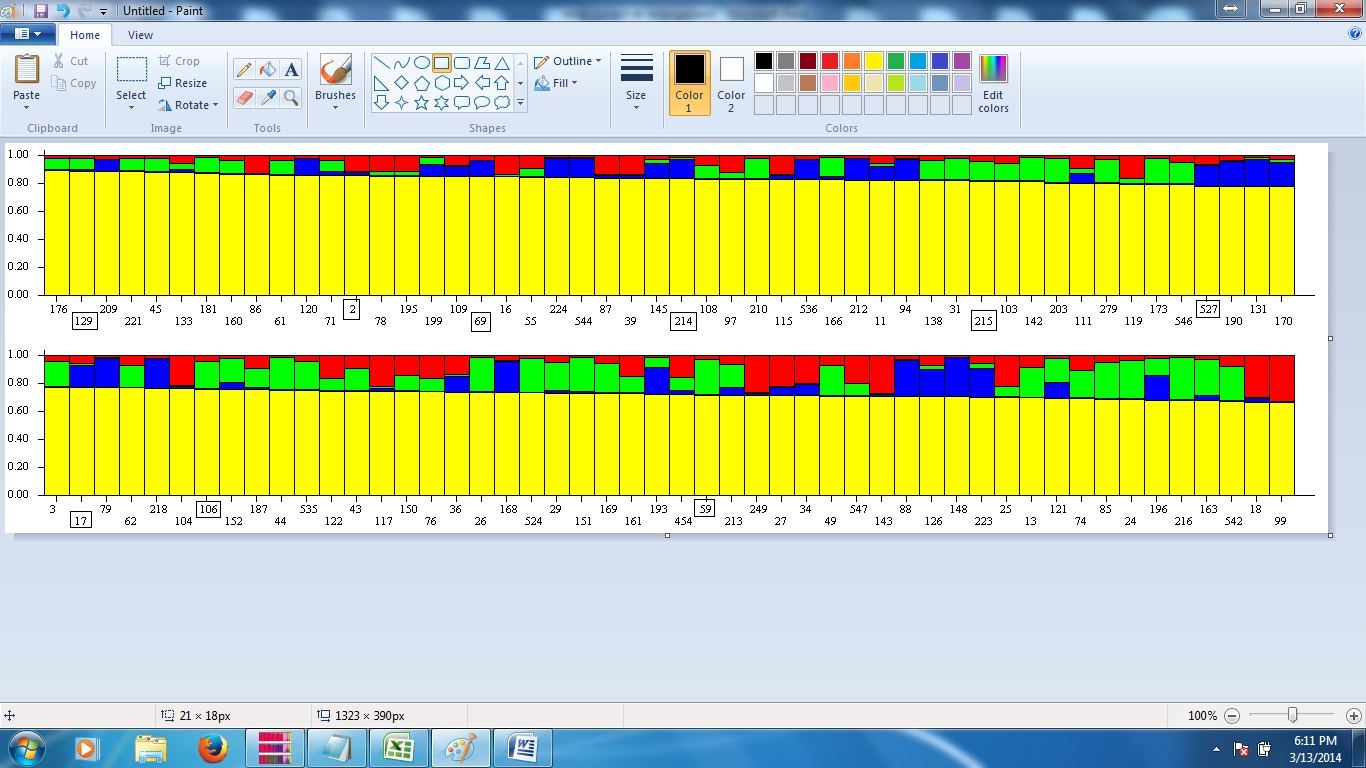


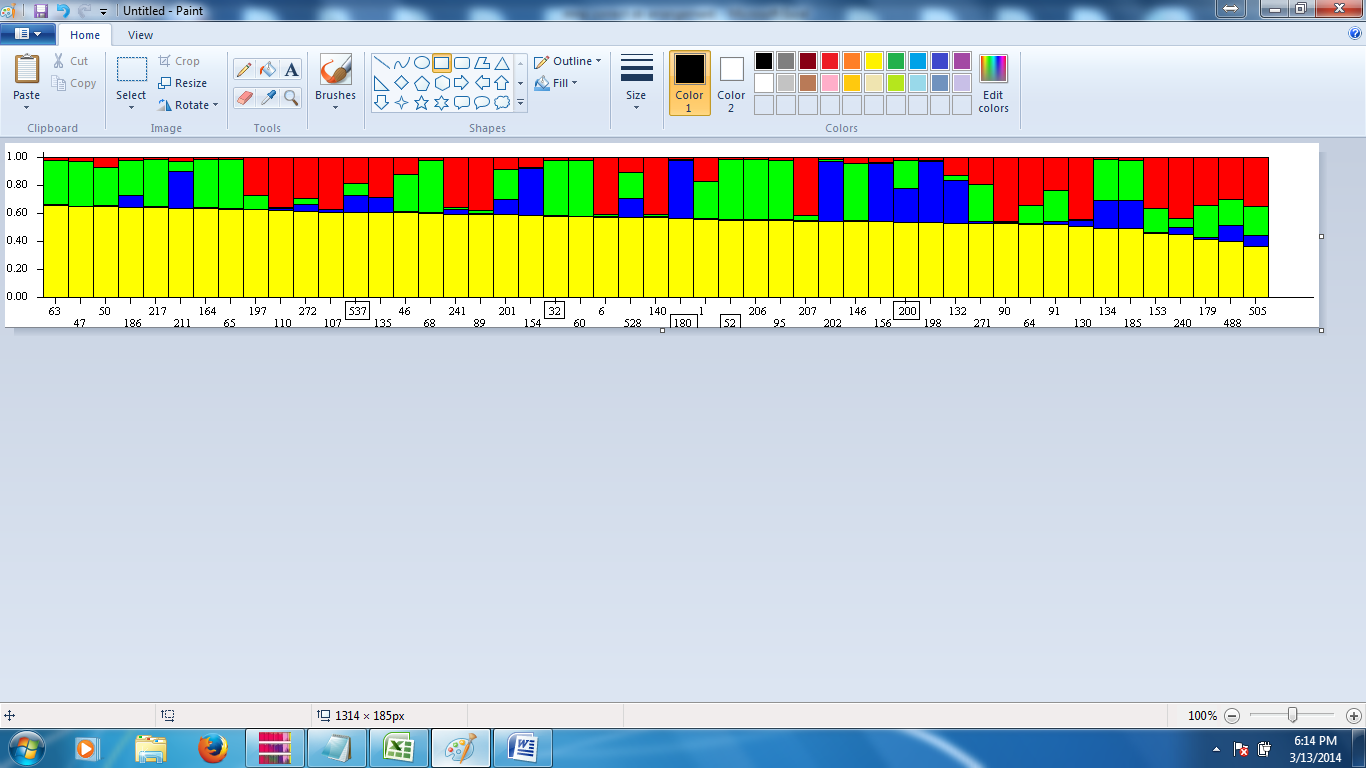


**Fig S3d Model based clustering of Meghalaya samples**


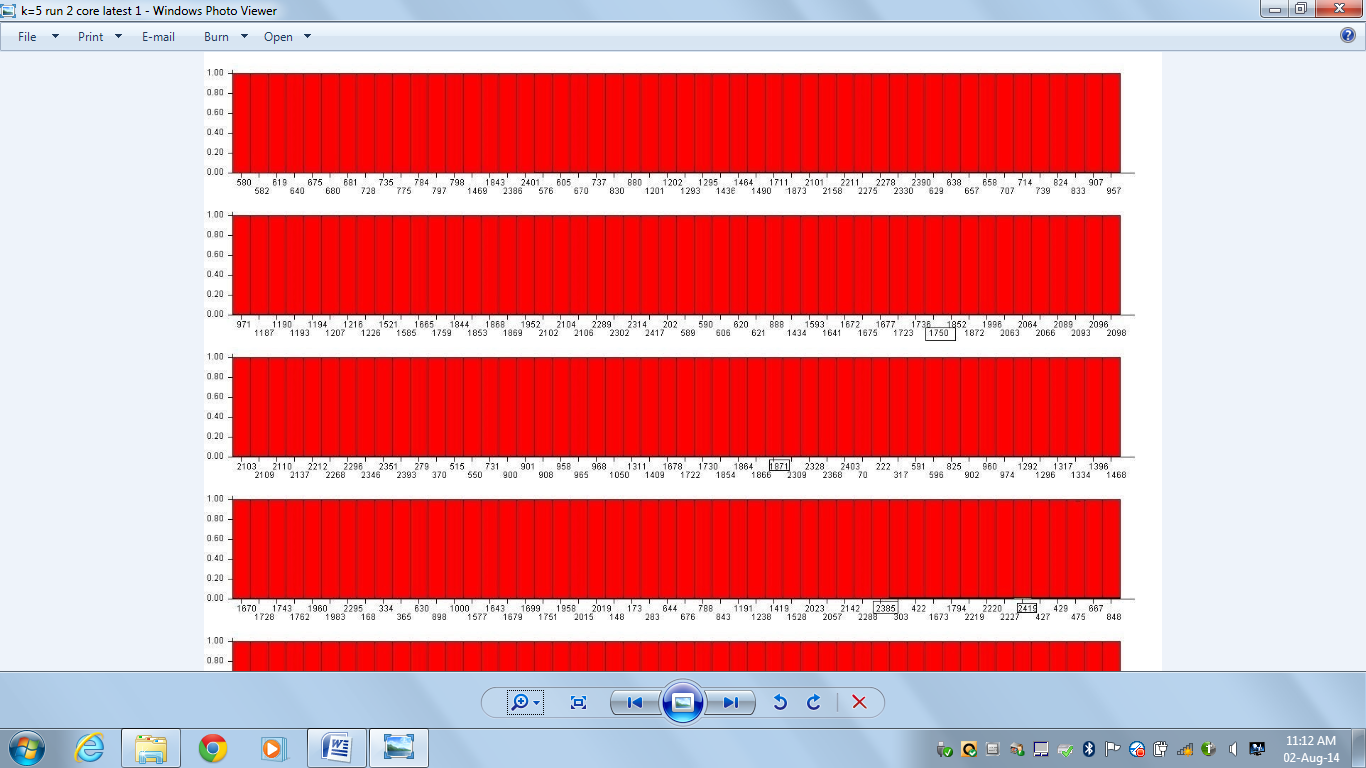


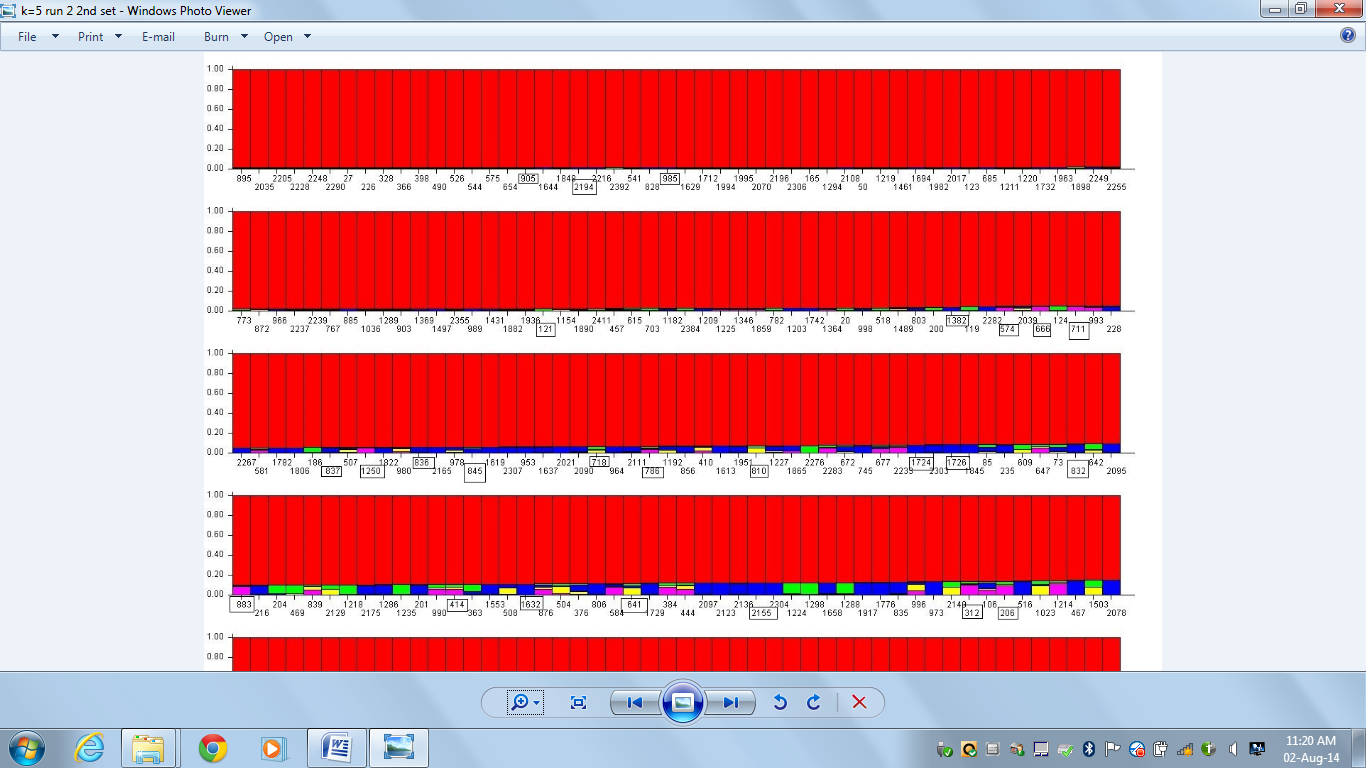


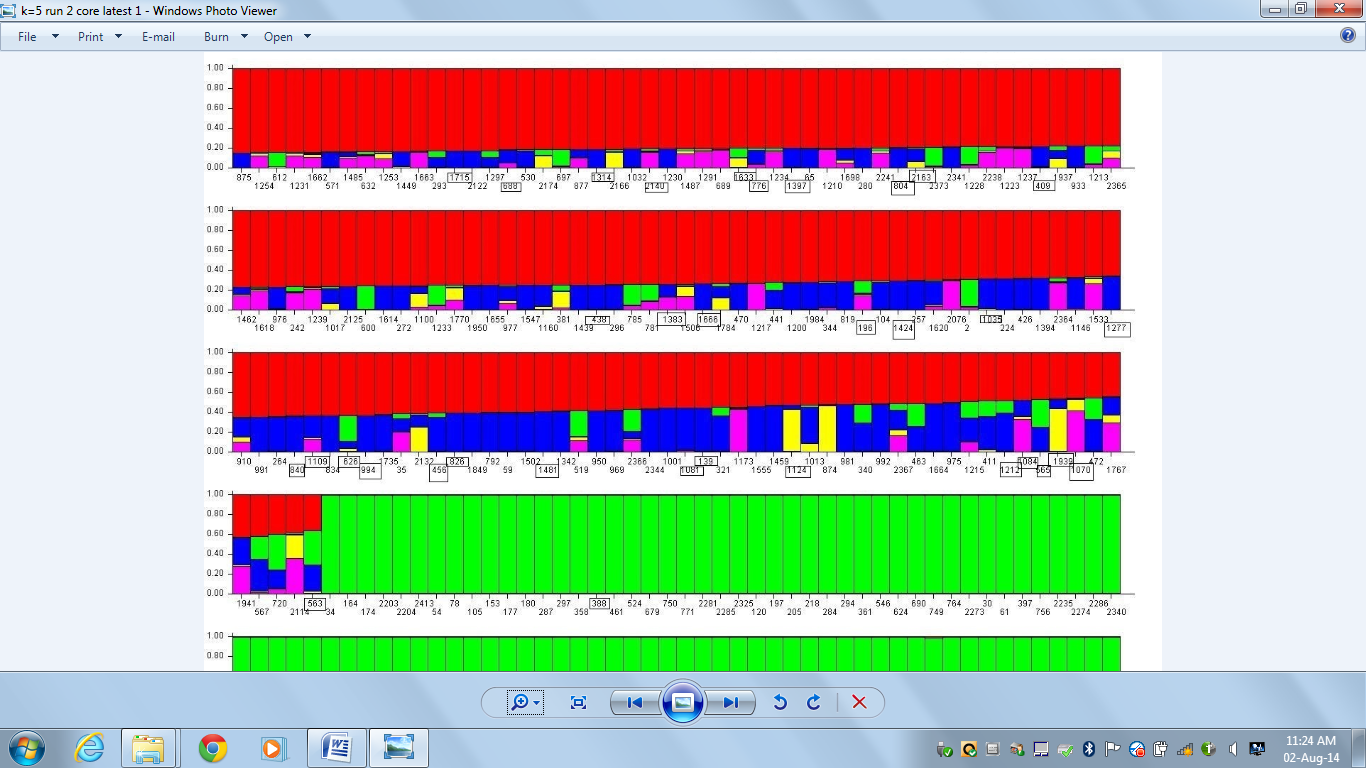


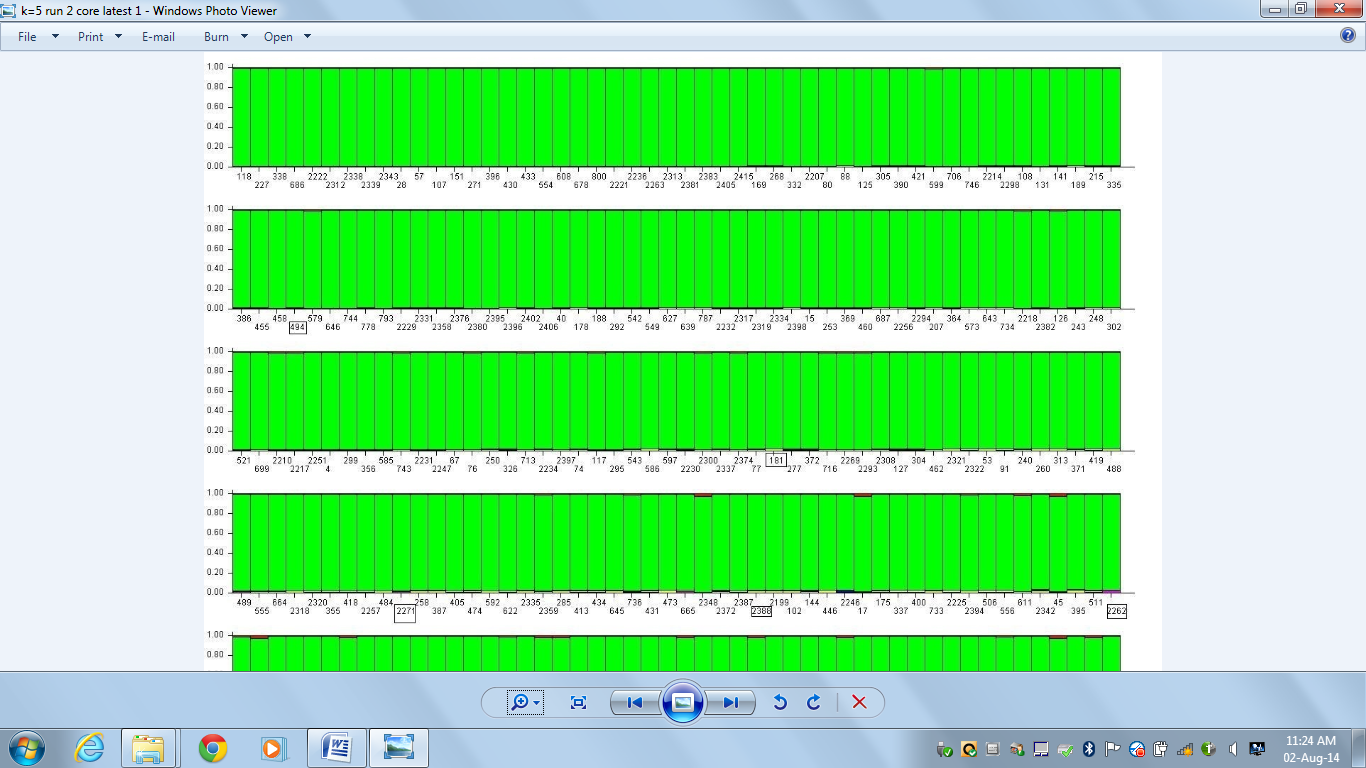


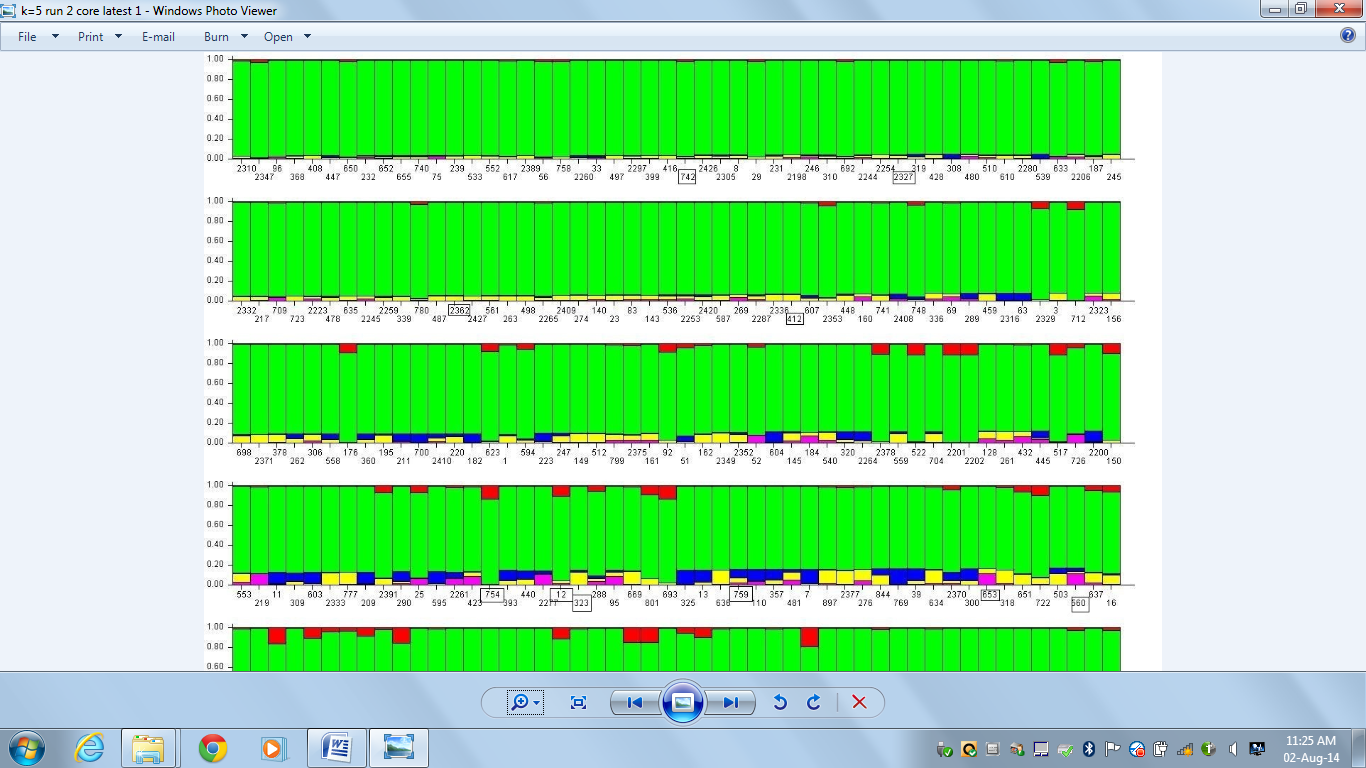


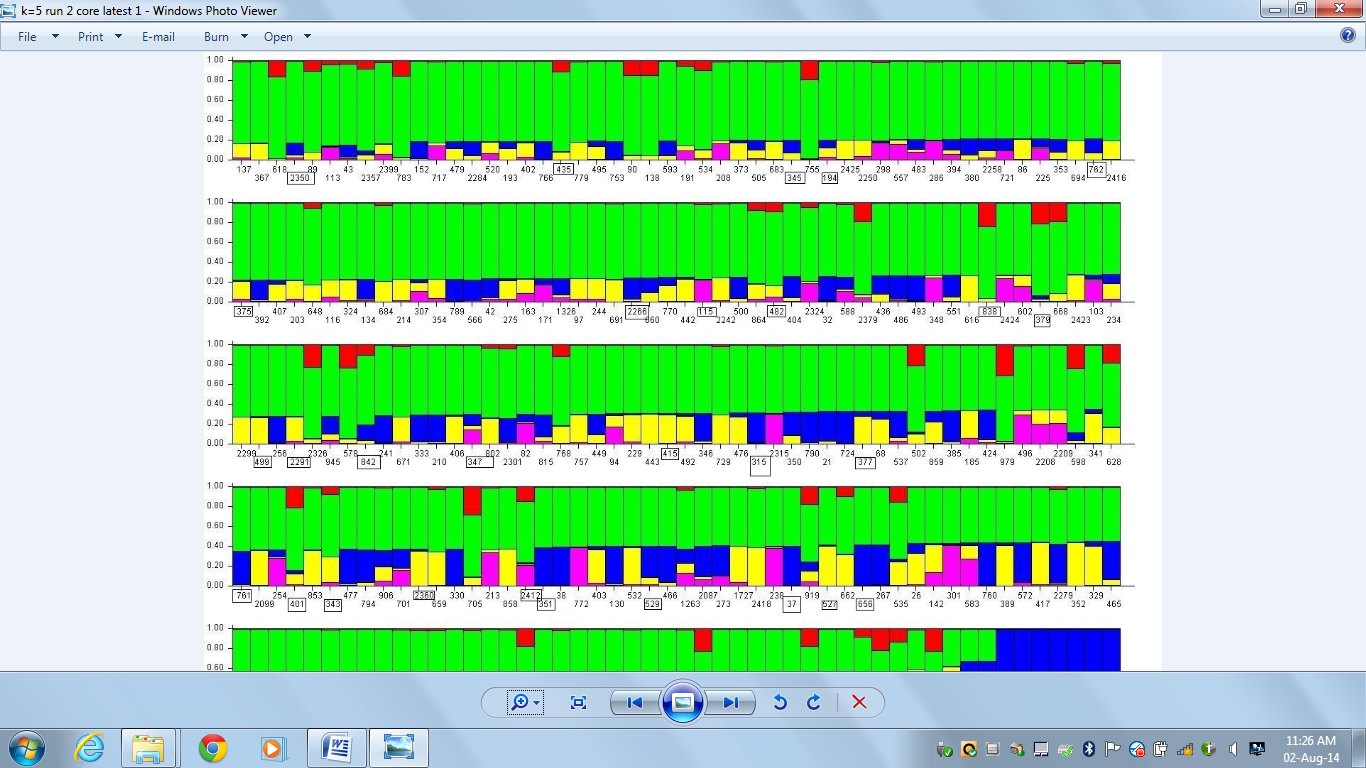


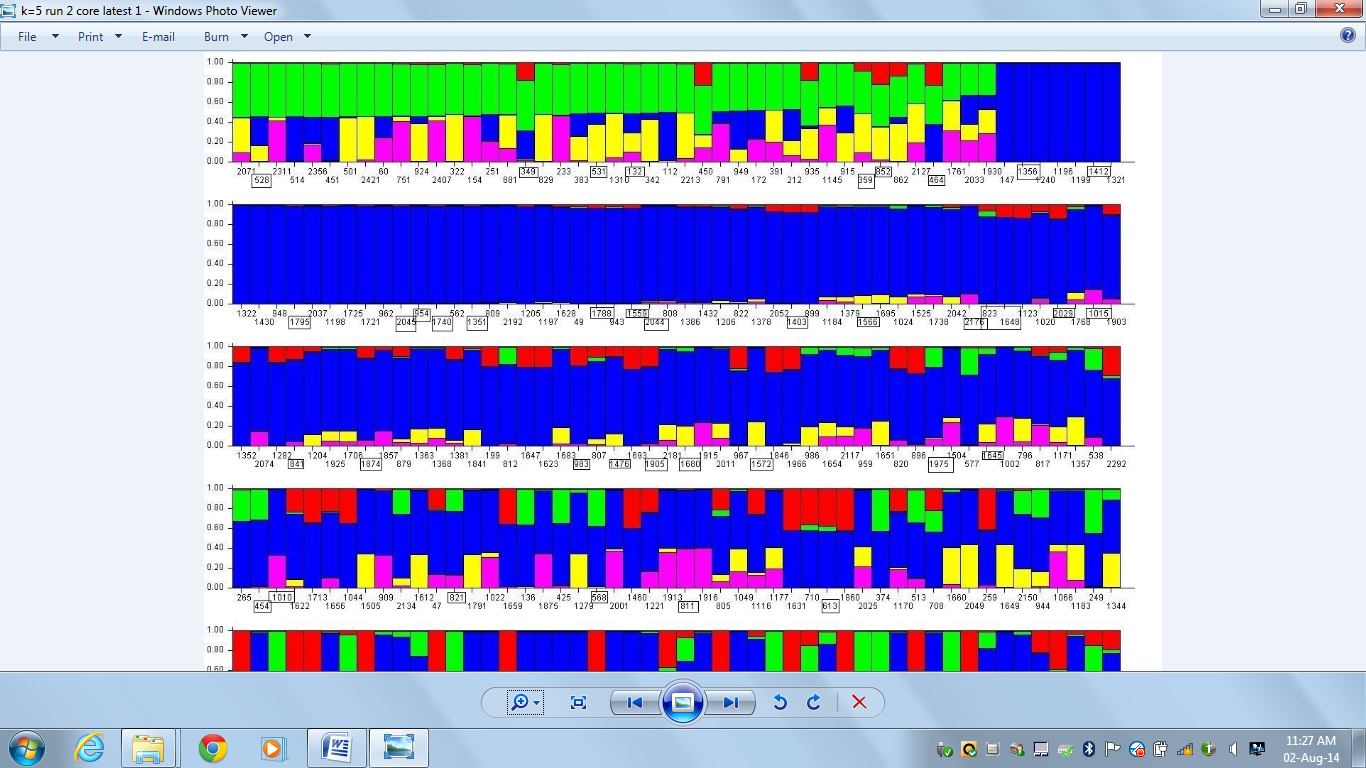


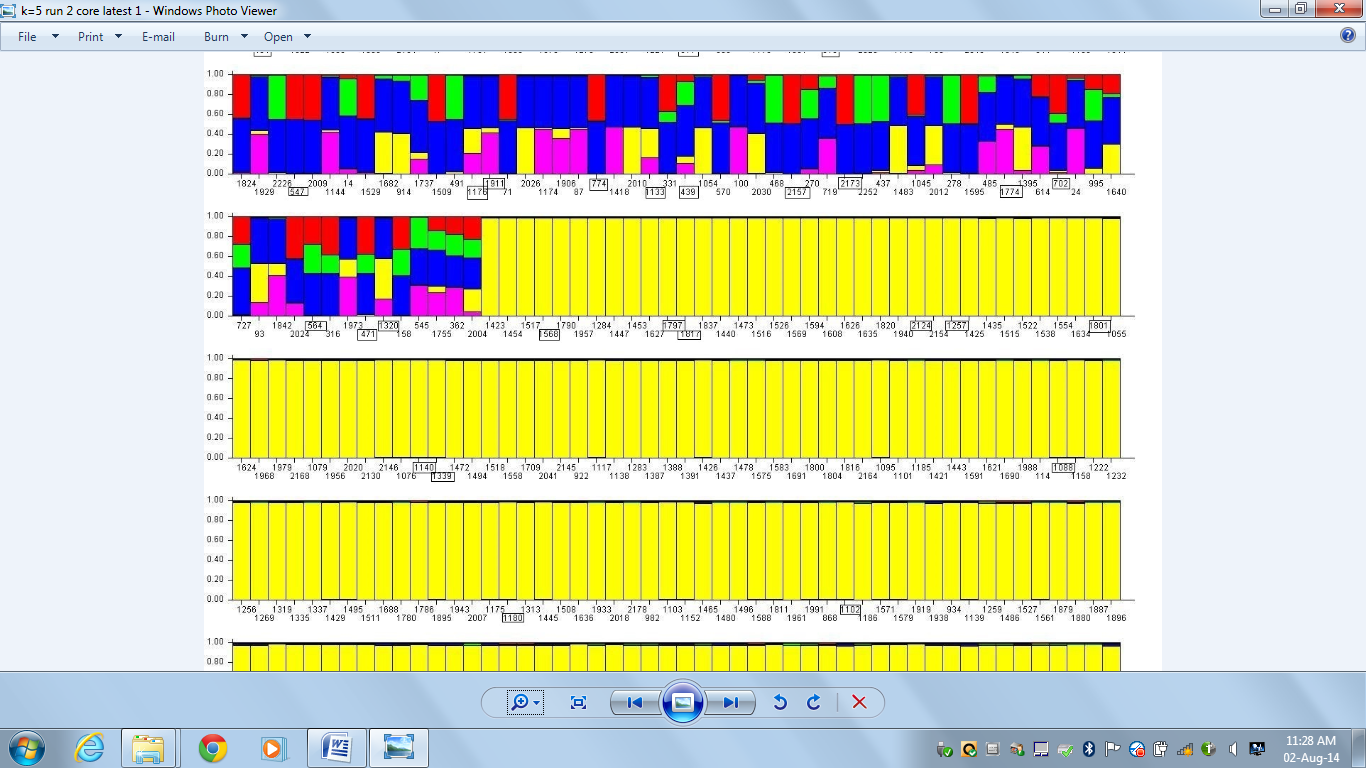


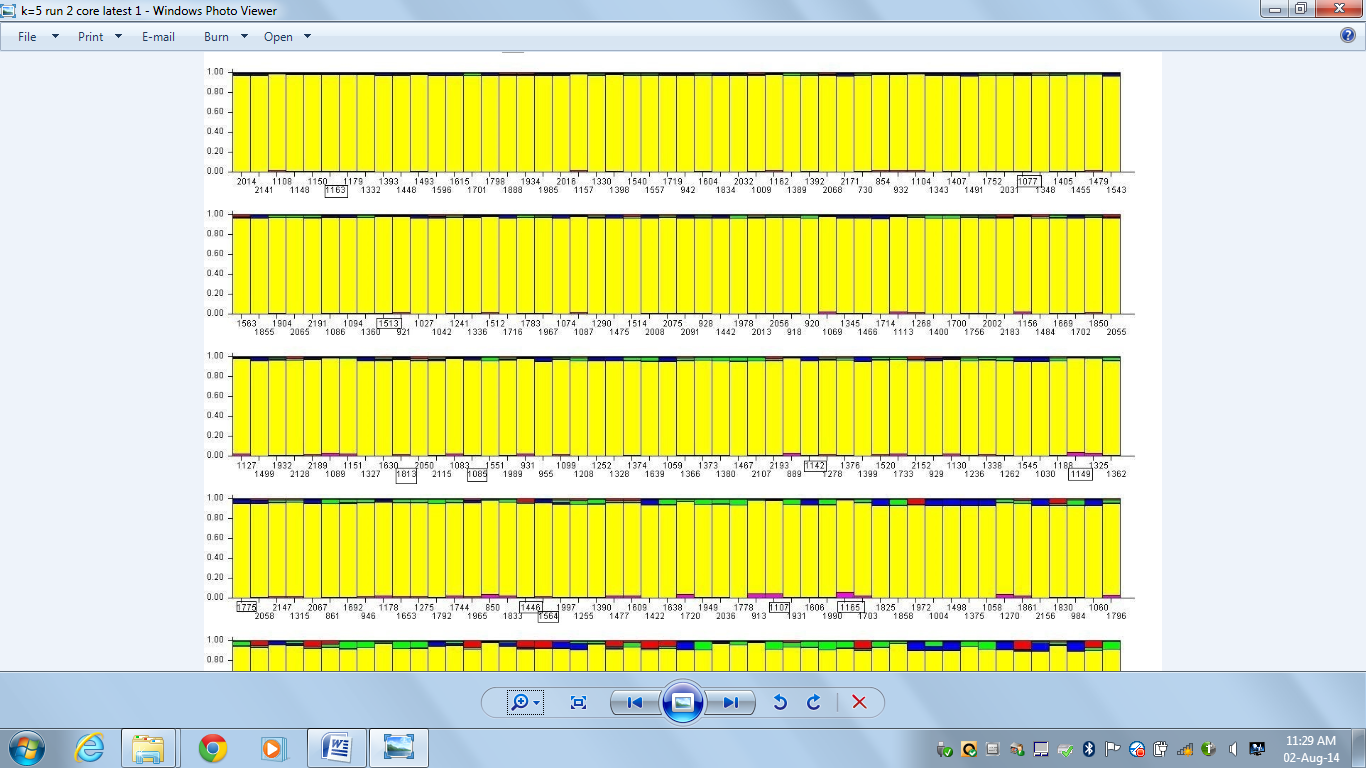


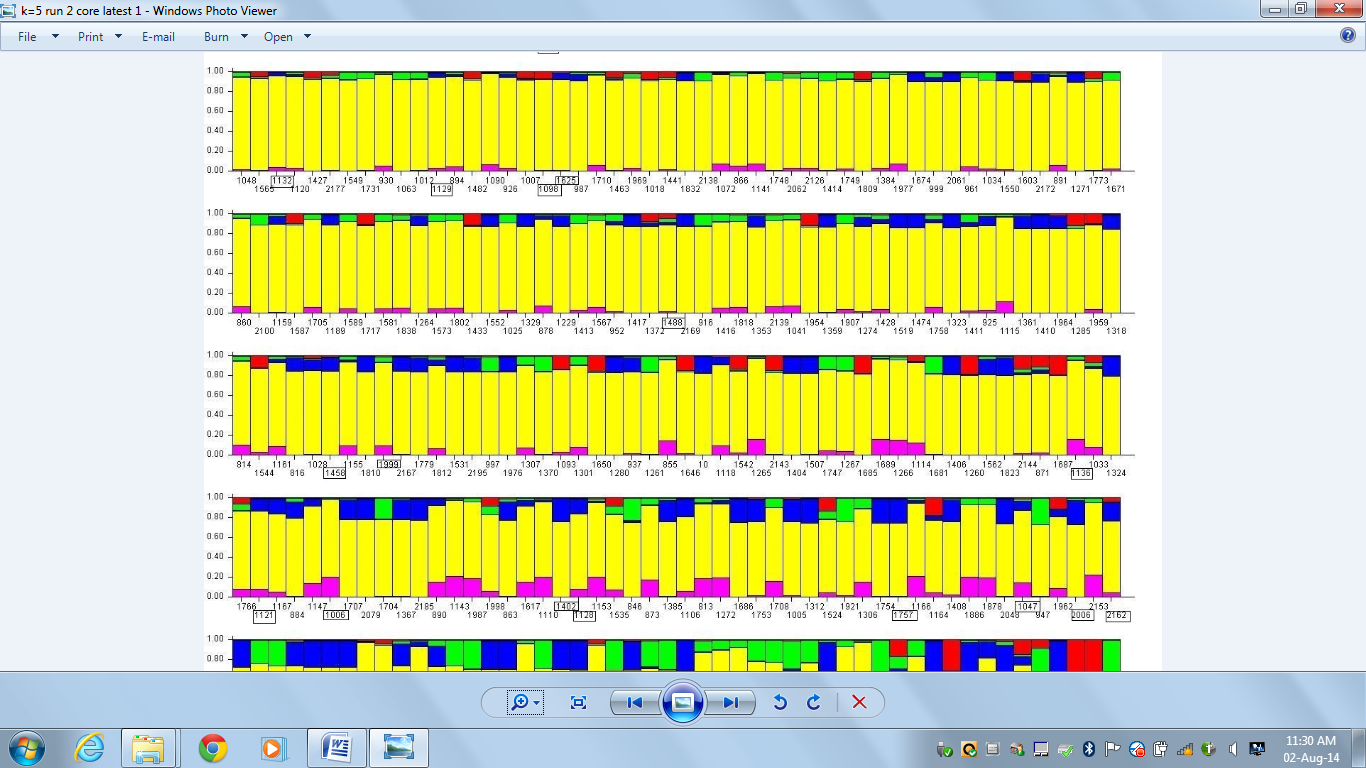


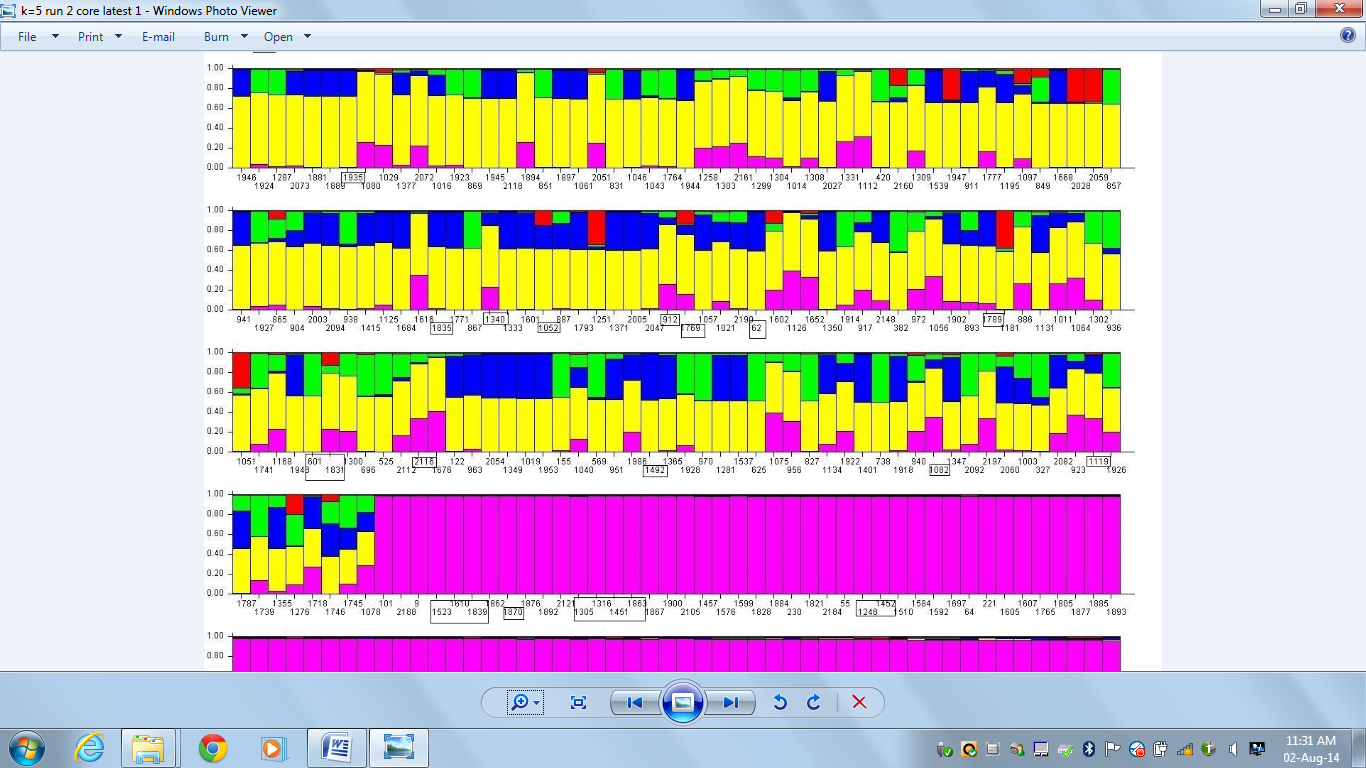


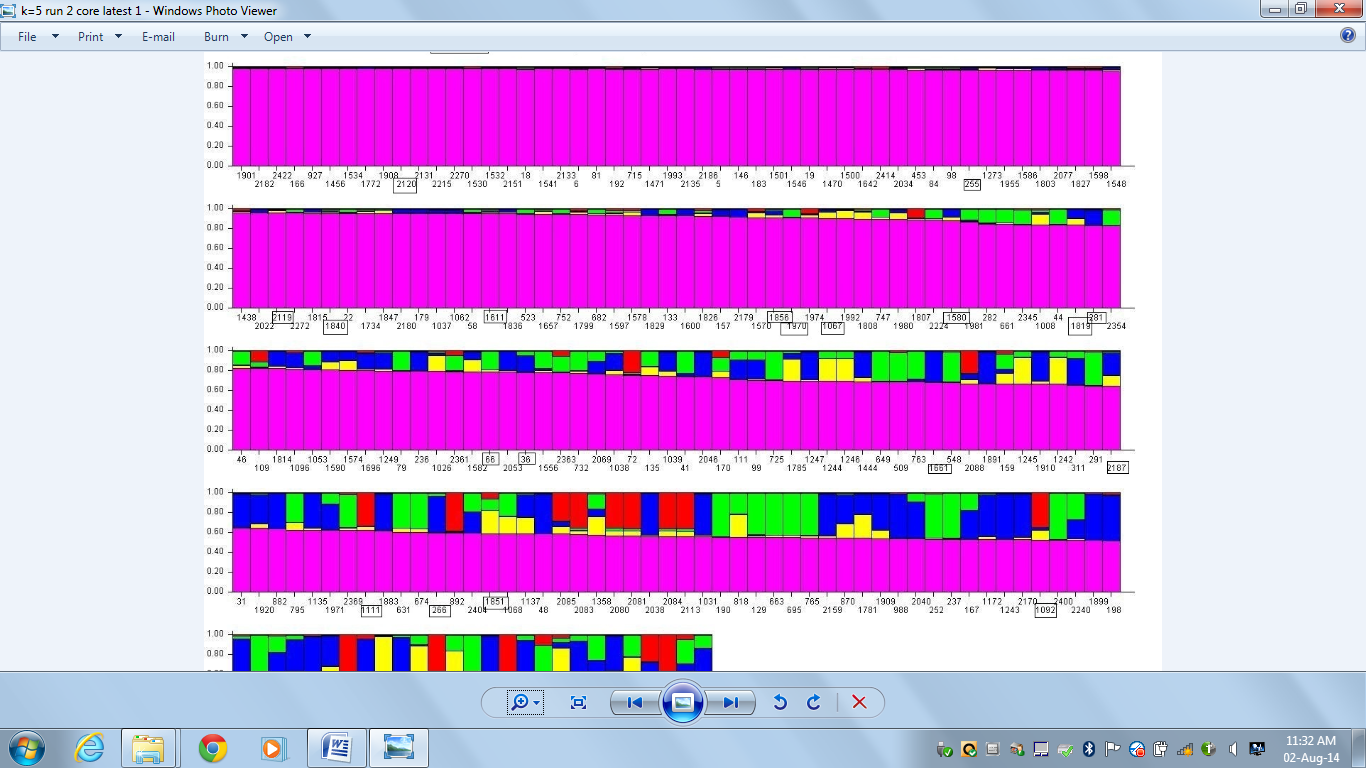


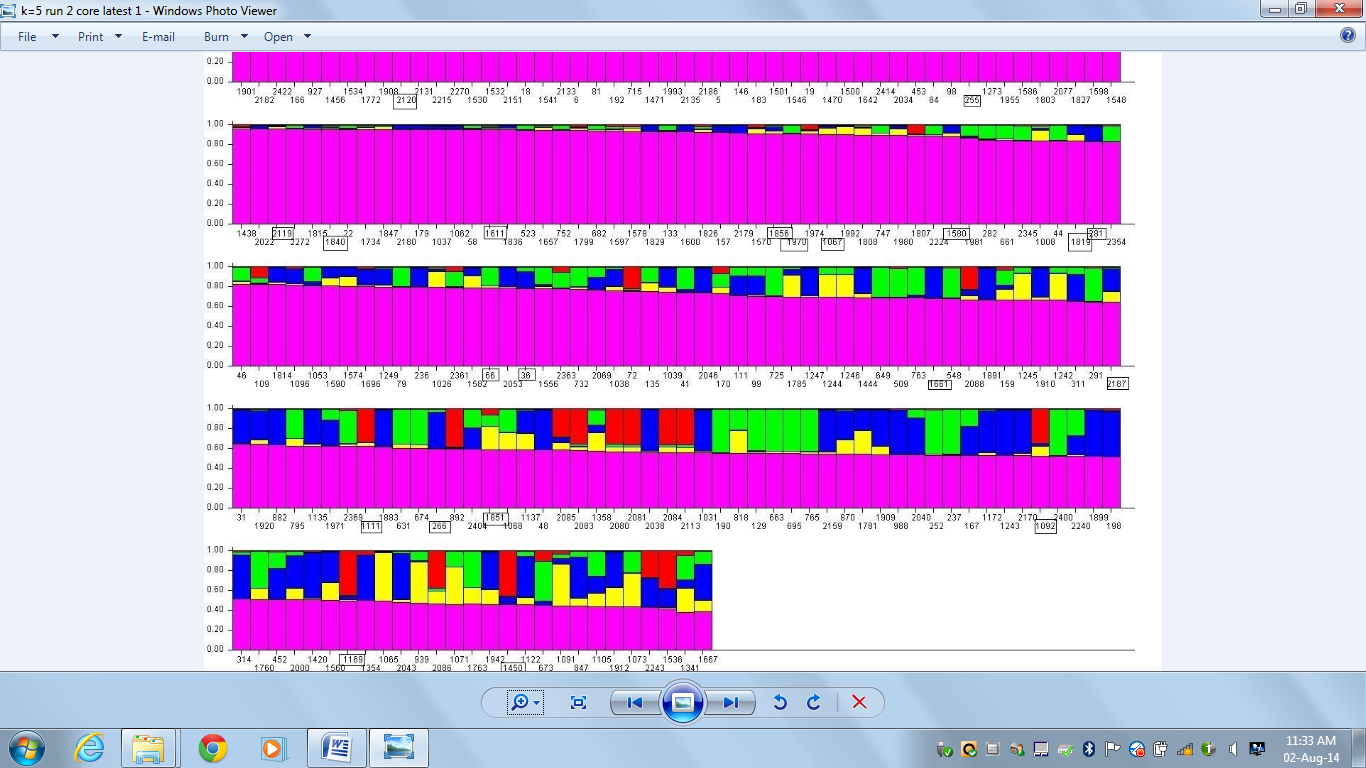


**Fig S3e Model based clustering of Mizoram**


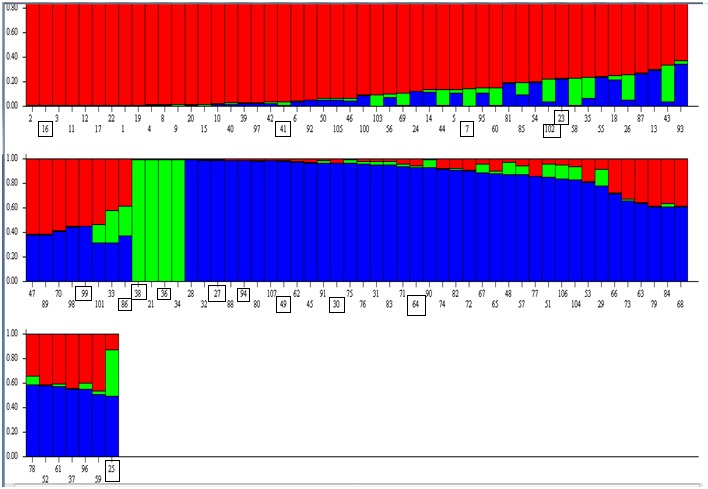


**Fig S3f Model based clustering of Nagaland**


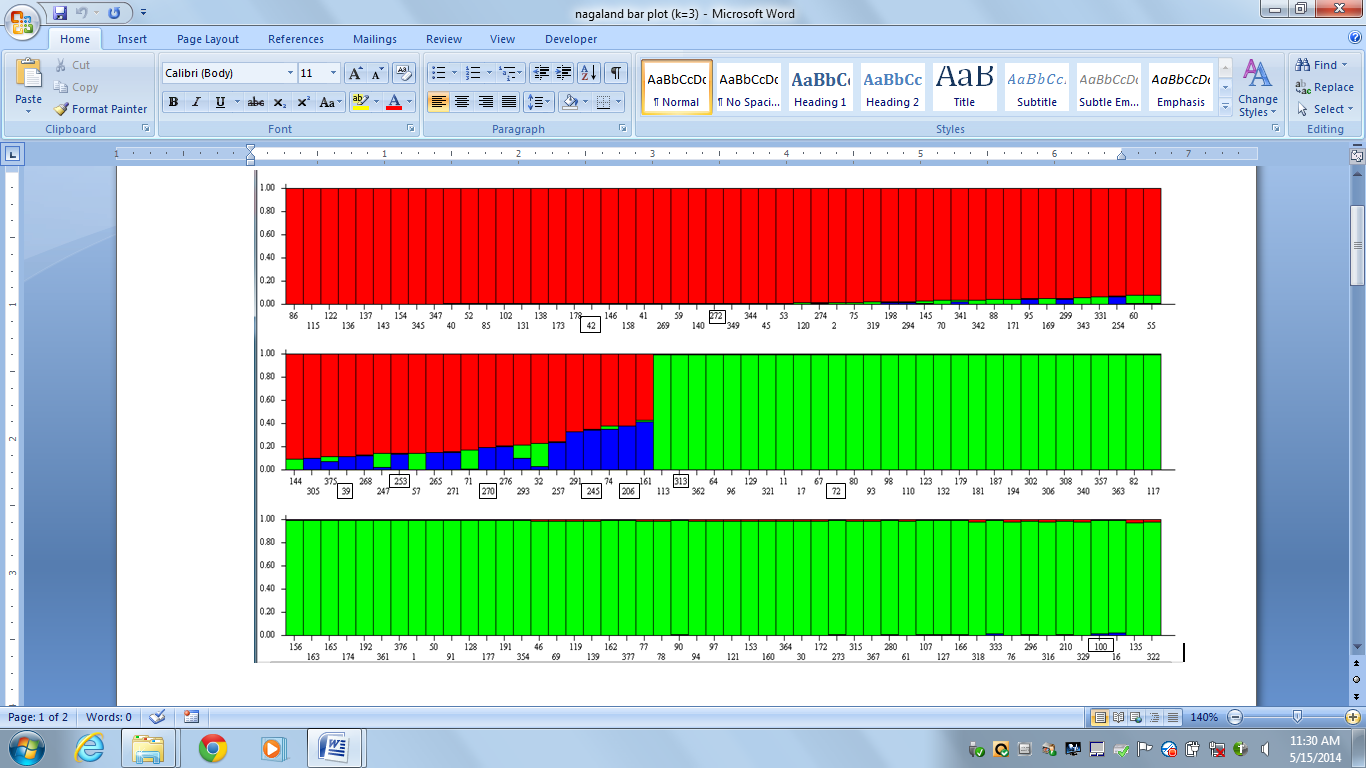


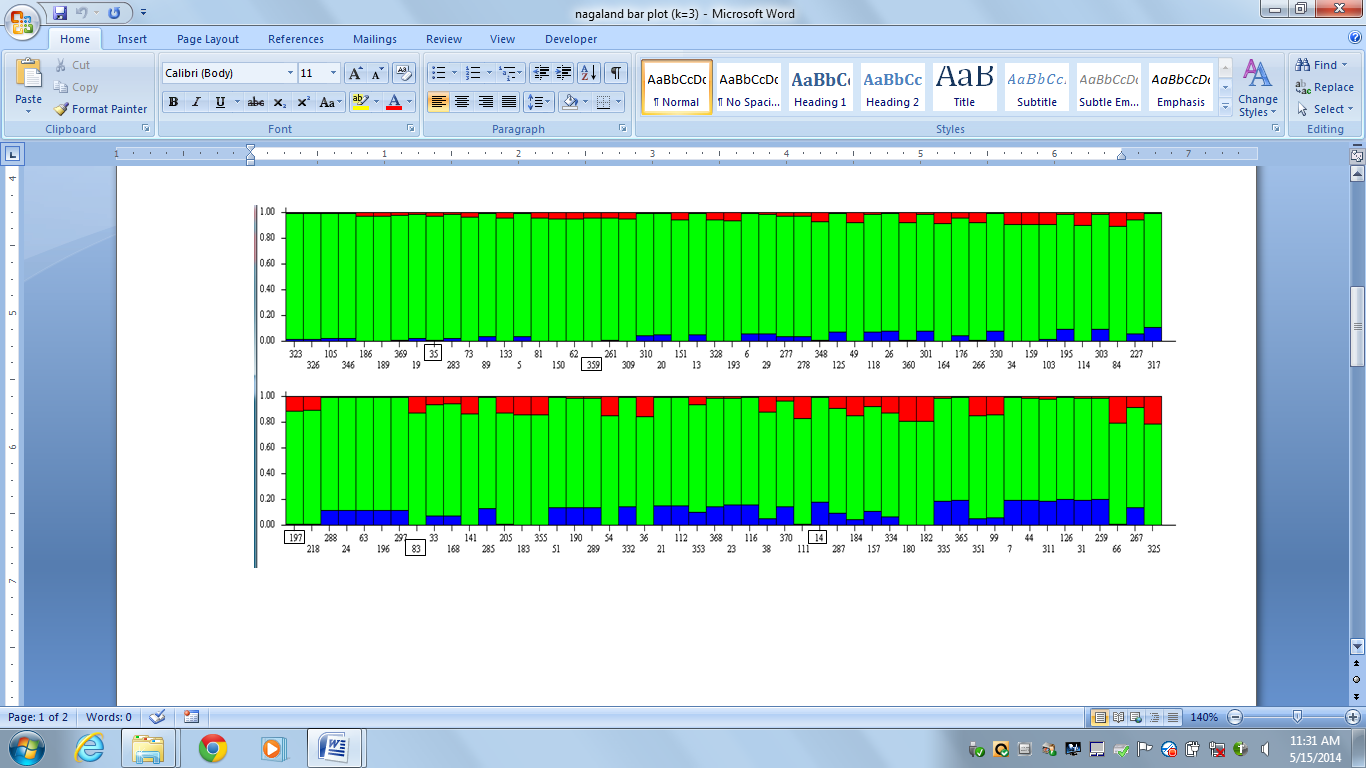


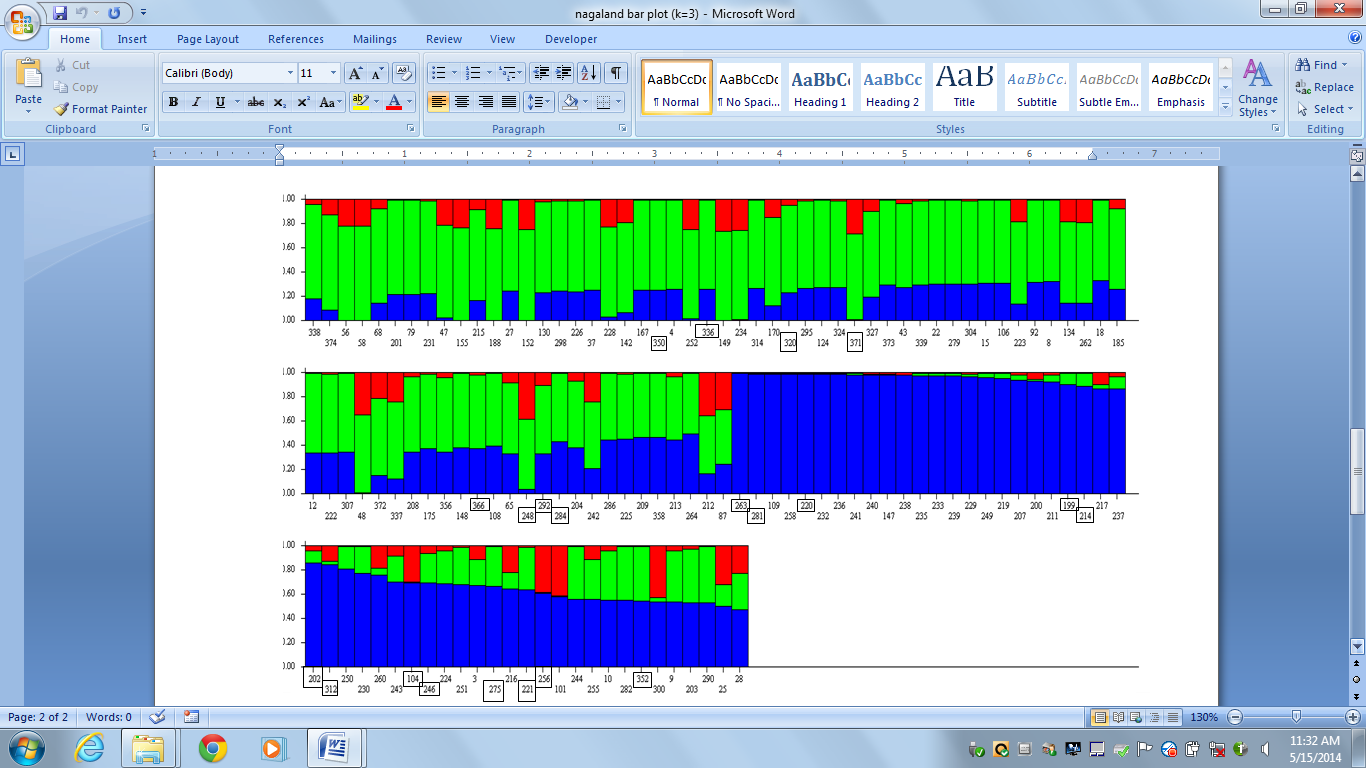


**Fig S3g Model based clustering of Tripura**


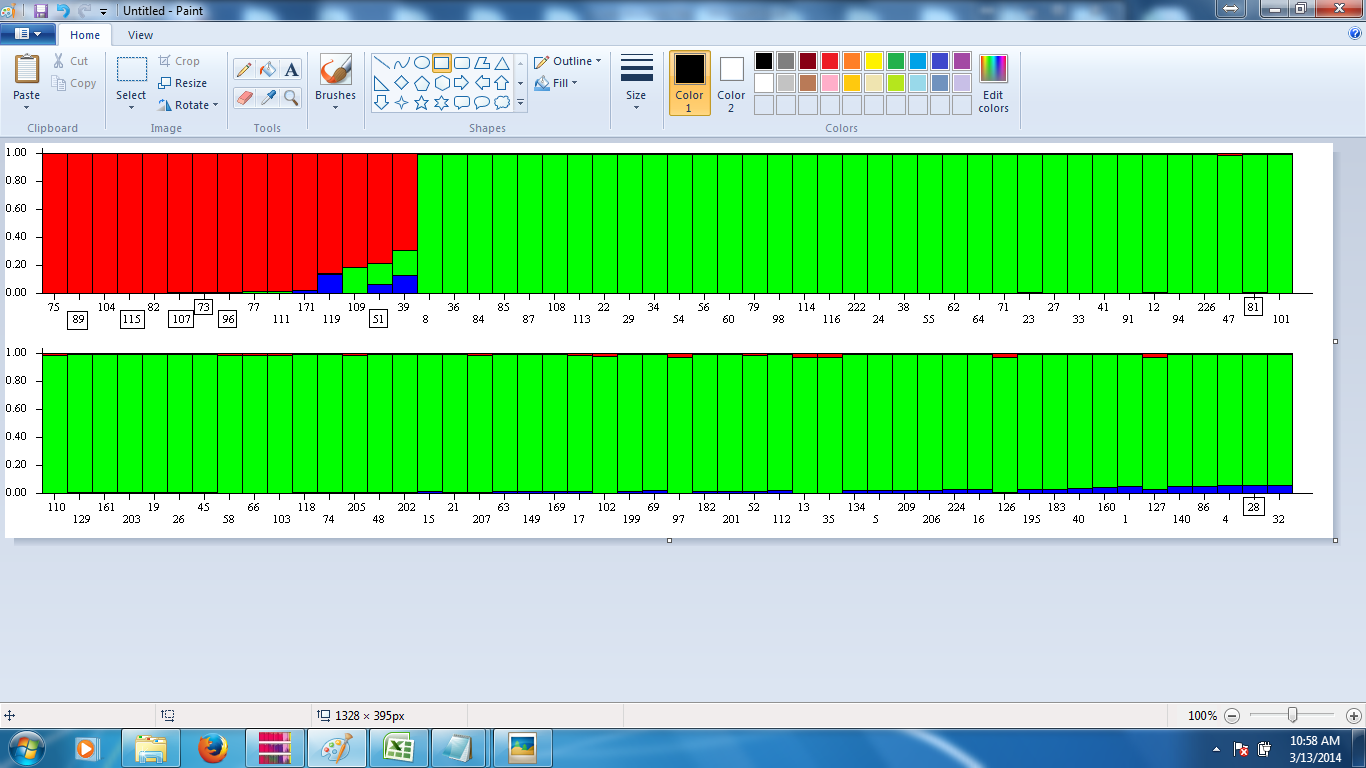


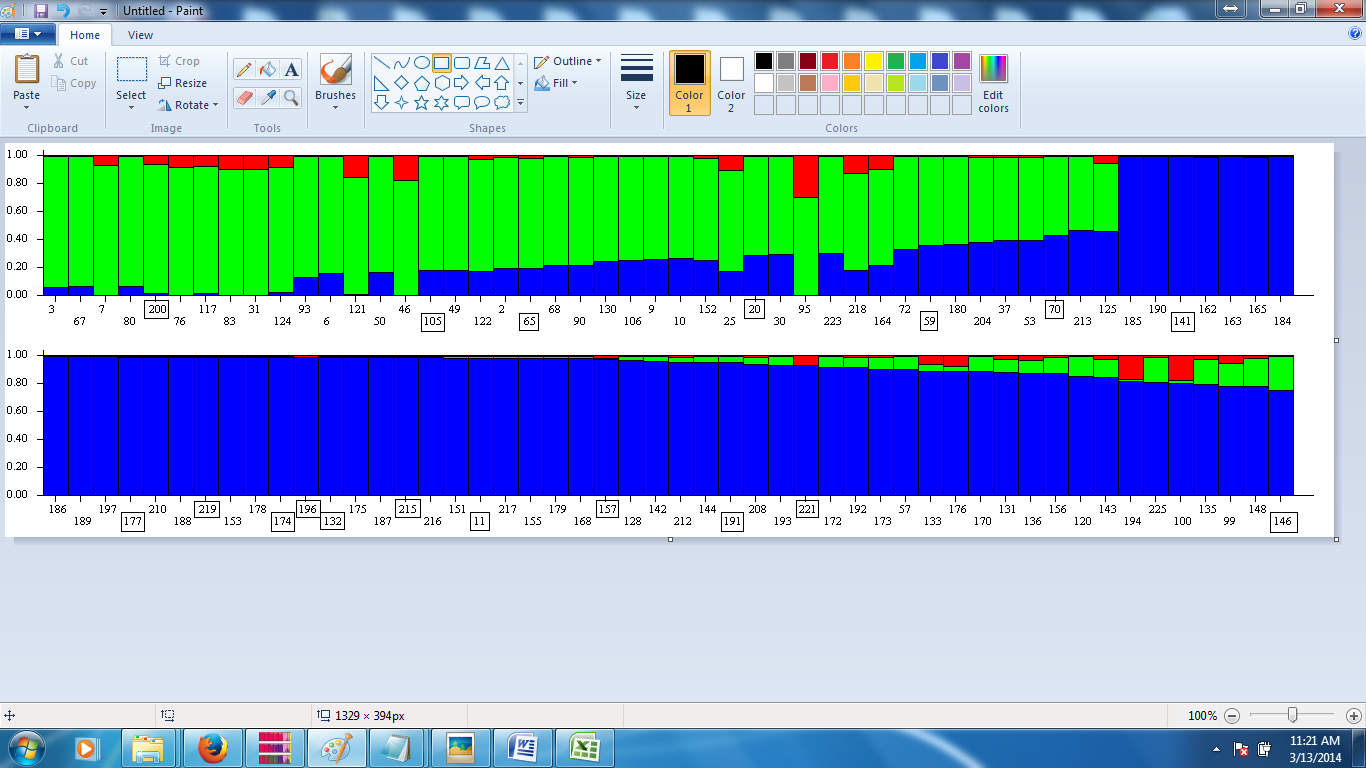


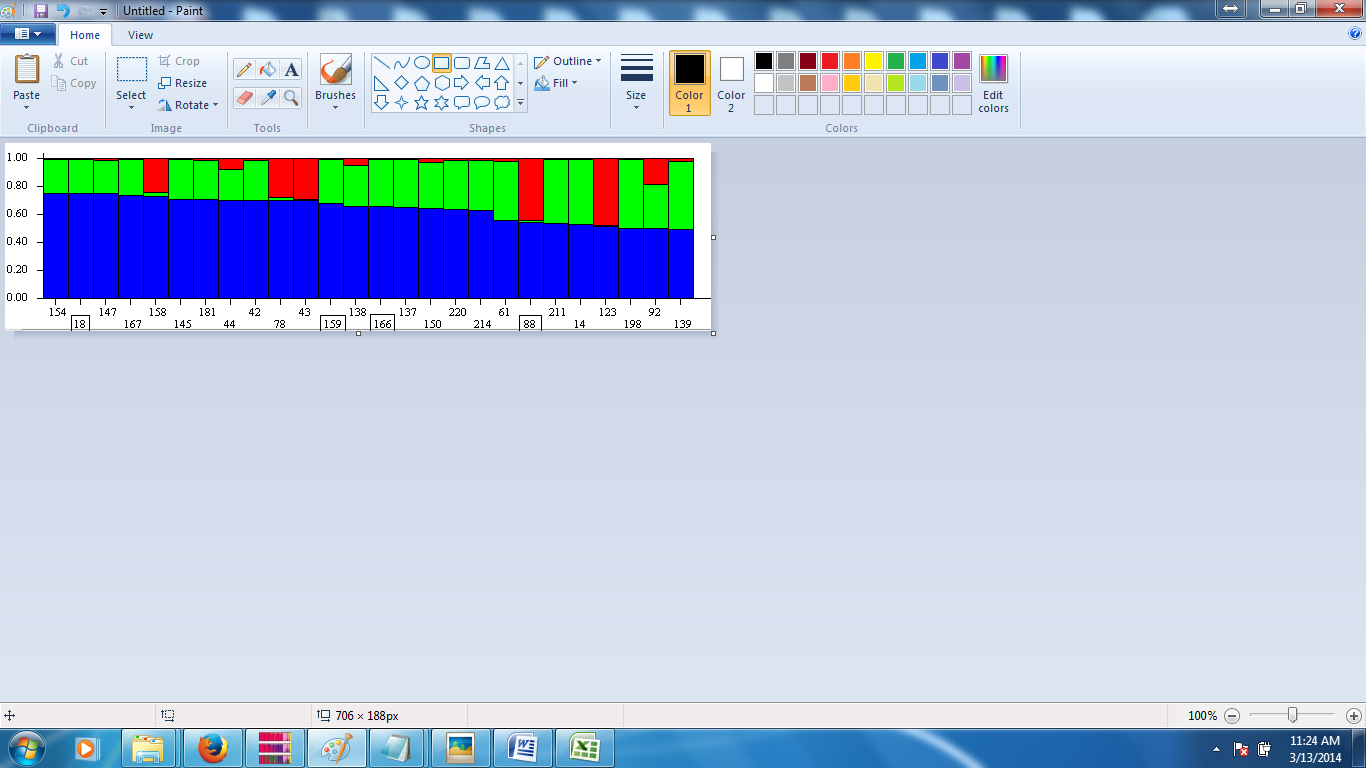

Supplement: Figure S3 — Population structure of NE rice collection based on SNP data (a) Arunachal Pradesh, (b) Assam, (c) Manipur, (d) Meghalaya, (e) Mizoram, (f) Nagaland and (g) Tripura. (DOCX) [file pone.0113094.s003.docx]
