# Supplementary material for: Analysis of Genetic Diversity and Population Structure of Rice Germplasm from North-Eastern Region of India and Development of a Core Germplasm Set
Source: PLoS One. 2014 Nov 20;9(11):e113094. doi: 10.1371/journal.pone.0113094 (PMC4239046; doi:10.1371/journal.pone.0113094)
Supplement: Figure S4 — Analysis of Molecular Variance in NE rice collection based on SNP data (a) Arunachal Pradesh, (b) Assam, (c) Manipur, (d) Meghalaya, (e) Mizoram, (f) Nagaland and (g) Tripura. (DOCX) [file pone.0113094.s004.docx]

1. **(b)**

Meghalaya

**(c) (d)**

**(e) (f)**

**(g)**

**Fig S4**
