# Supplementary material for: Analysis of Genetic Diversity and Population Structure of Rice Germplasm from North-Eastern Region of India and Development of a Core Germplasm Set
Source: PLoS One. 2014 Nov 20;9(11):e113094. doi: 10.1371/journal.pone.0113094 (PMC4239046; doi:10.1371/journal.pone.0113094)
Supplement: Figure S5 — Principal Coordinate Analysis in NE rice collection based on SNP data (a) Arunachal Pradesh, (b) Assam, (c) Manipur, (d) Meghalaya, (e) Mizoram, (f) Nagaland and (g) Tripura. (DOCX) [file pone.0113094.s005.docx]

Arunachal Pradesh

1^st^ quadrant

2^nd^ quadrant

3^rd^ quadrant

4^th^ quadrant

**Fig S5a**

Assam

1^st^ quadrant

2^nd^ quadrant

3^rd^ quadrant

4^th^ quadrant

**Fig S5b**

Manipur

1^st^ quadrant

2^nd^ quadrant

3^rd^ quadrant

4^th^ quadrant

**Fig S5c**

Meghalaya

1^st^ quadrant

2^nd^ quadrant

3^rd^ quadrant

4^th^ quadrant

**Fig S5d**

Mizoram

1^st^ quadrant

2^nd^ quadrant

3^rd^ quadrant

4^th^ quadrant

**Fig S5e**

Nagaland

1^st^ quadrant

2^nd^ quadrant

3^rd^ quadrant

4^th^ quadrant

**Fig S5f**

Tripura

4^th^ quadrant

3^rd^ quadrant

2^nd^ quadrant

1^st^ quadrant

**Fig S5g**
