# Supplementary material for: Analysis of Genetic Diversity and Population Structure of Rice Germplasm from North-Eastern Region of India and Development of a Core Germplasm Set
Source: PLoS One. 2014 Nov 20;9(11):e113094. doi: 10.1371/journal.pone.0113094 (PMC4239046; doi:10.1371/journal.pone.0113094)
Supplement: Table S2 — List of SNP primers used for genotyping of NE rice collection (6984 rice accessions) alongwith gene diversity, PIC and Major allele frequency. (DOCX) [file pone.0113094.s007.docx]

Table S2. SNP primers used for genotyping of 6984 rice alongwith gene diversity, PIC and Major allele frequency primer wise.

| Chr. No. | Primer ID | Physical position | Amplification primer1 | Amplification primer2 | Gene Diversity | Heterozygosity | PIC | Major Allele freq. |
| --- | --- | --- | --- | --- | --- | --- | --- | --- |
|  |  |  |  |  |  |  |  |  |
| 1 | 01-3916-1_C_156 | 25381654 | ACGTTGGATGGGGTTTGCATGTTAATAGGG | ACGTTGGATGCCGAATCTCTATCAAGGAAG | 0.44 | 0.08 | 0.34 | 0.64 |
|  | 01-608-4_C_375 | 3421011 | ACGTTGGATGAGGACCATCTTCTTGCACTG | ACGTTGGATGCCATTTGCAAGGCCCATTTC | 0.49 | 0.20 | 0.37 | 0.54 |
|  | 01-6351-1_C_202 | 40914292 | ACGTTGGATGGTTGGAACACATGATTTCAC | ACGTTGGATGATCTCTTTGGACAGAGTCCC | 0.26 | 0.08 | 0.22 | 0.84 |
| 2 | 02-267 | 1570149 | ACGTTGGATGGTCAATCTTGCAGGAGTTGG | ACGTTGGATGTGGCTCCTCTTCTCCGGTCT | 0.44 | 0.18 | 0.34 | 0.64 |
|  | 02-3029-1_C_474 | 18821156 | ACGTTGGATGTGTCTGCAATAACTTGTGCC | ACGTTGGATGAAATCAGCTGCAGCATTACC | 0.48 | 0.18 | 0.37 | 0.58 |
|  | 02-4333-1_C_293 | 28688819 | ACGTTGGATGGGAATGTTTAGTTTTGAGG | ACGTTGGATGTGTAGGTGCTACTTGCTTCC | 0.33 | 0.07 | 0.27 | 0.78 |
| 3 | 03-1691-1_C_373 | 10849512 | ACGTTGGATGAACAACGCCAGGAACATCAC | ACGTTGGATGAAGCGGCTCAAGGTACAATC | 0.40 | 0.10 | 0.32 | 0.72 |
|  | 03-3478-1_C_206 | 22815422 | ACGTTGGATGCCTGCAGCAAACGCCAATTT | ACGTTGGATGTCAGGTAACCGATCGATTTG | 0.49 | 0.20 | 0.37 | 0.58 |
|  | 03-4660-1_C_355 | 31020366 | ACGTTGGATGCTCCCATCCTAGTATCCATC | ACGTTGGATGTGCCTTCTCTTACAGGTTCC | 0.37 | 0.09 | 0.30 | 0.74 |
| 4 | 04-1801-20_C_428 | 11859836 | ACGTTGGATGCCCTCAAAAAAAAGTTGTAAG | ACGTTGGATGCAGTAAATTTCCAGGGAGATA | 0.43 | 0.28 | 0.34 | 0.65 |
|  | 04-19-4_C_240 | 225838 | ACGTTGGATGTCTACACATTAGCTCGCTGG | ACGTTGGATGACAGTAACCACAATATGCCG | 0.02 | 0.01 | 0.02 | 0.99 |
|  | 04-3787-3_C_358 | 25211800 | ACGTTGGATGTTATCTCTGCTTGCTCGCTC | ACGTTGGATGAAGTATCTGCCCCAAGTGAC | 0.37 | 0.13 | 0.30 | 0.74 |
| 5 | 05-2692-1_C_109 | 18783426 | ACGTTGGATGGAACTTTACTCTCAGTACA | ACGTTGGATGTGGTTTGATGAGTCGTTTGC | 0.25 | 0.07 | 0.22 | 0.85 |
|  | 05-4192-1_C_280 | 28065769 | ACGTTGGATGAGTTTGTTGACAGCAGAACC | ACGTTGGATGTAGCTTACTAGTTCATGTG | 0.45 | 0.14 | 0.35 | 0.65 |
|  | 05-48-1_C_279 | 287362 | ACGTTGGATGCAGAGATGTCTGTTGTTAGC | ACGTTGGATGCAACCAGGGATACAATATGAC | 0.49 | 0.18 | 0.37 | 0.58 |
| 6 | 06-1256-1_C_147 | 7573979 | ACGTTGGATGCACGTGCCTATGATTAGCAG | ACGTTGGATGGATCGTTTACTTCTTTGCCC | 0.05 | 0.02 | 0.05 | 0.97 |
|  | 06-1776-1_C_501 | 11093772 | ACGTTGGATGGGGCCAATTTGCTTAGTGC | ACGTTGGATGAGCATAAGGTATTAAAGTC | 0.41 | 0.15 | 0.32 | 0.70 |
|  | 06-2509-1_C_497 | 15737387 | ACGTTGGATGCCTTCGCGCTTGCAATTTGG | ACGTTGGATGAAATCAGCACGCGTCAACAC | 0.22 | 0.08 | 0.19 | 0.87 |
| 7 | 07-2904-39_C_299 | 19160255 | ACGTTGGATGAATGGTGGTGTATCTTGAGC | ACGTTGGATGGGTGTGACTTCTCATGACAG | 0.16 | 0.05 | 0.14 | 0.91 |
|  | 07-293-12_C_368 | 1859603 | ACGTTGGATGCACTAATTCTTGGTATTATGG | ACGTTGGATGTCAATGTGTTCTCACAGACC | 0.18 | 0.03 | 0.16 | 0.90 |
|  | 07-4304 |  |  |  | 0.41 | 0.05 | 0.33 | 0.69 |
| 8 | 08-2765-2_C_360 | 18084851 | ACGTTGGATGTCCCTCCATGTTGTGAGTTC | ACGTTGGATGCTTGCAAGAGACATCCAAGA | 0.15 | 0.02 | 0.13 | 0.92 |
|  | 08-4218-5_C_129 | 27692470 | ACGTTGGATGGGTGGACAAAGATAAGGAAG | ACGTTGGATGGACTGGAAATATACTCCCTC | 0.48 | 0.15 | 0.37 | 0.57 |
|  | 08-847-6_C_113 | 5399913 | ACGTTGGATGCCCAACGTATTAATGGCAAC | ACGTTGGATGGCTGTGTAGTAATTTGCCTG | 0.45 | 0.16 | 0.35 | 0.62 |
| 9 | 09-209 | 1297966 | ACGTTGGATGGAGGCAAAAGGCAAACCGAC | ACGTTGGATGGACTTGAGCGAGTCGATGTC | 0.22 | 0.04 | 0.19 | 0.87 |
|  | 09-2107-5_C_145 | 13705487 | ACGTTGGATGTGACCACACCACACAAACAC | ACGTTGGATGGGGATTTGCGGTTTTTGGAC | 0.37 | 0.11 | 0.30 | 0.74 |
|  | 09-2716-4_C_457 | 19541336 | ACGTTGGATGTGAGCCACAGATTCCCTTTC | ACGTTGGATGCTCGAGTAATTCAAAACCAC | 0.09 | 0.02 | 0.08 | 0.95 |
| 10 | 10-1192-7_C_178 | 8122635 | ACGTTGGATGCTTTGCTACGGATAAAATG | ACGTTGGATGTCATGCAAATACAGACATGG | 0.45 | 0.19 | 0.35 | 0.65 |
|  | 10-188-1 | 1218215 | ACGTTGGATGGCGCCAGTGTATGGAAAAAG | ACGTTGGATGGTCCATAACATCATGGACTC | 0.32 | 0.16 | 0.27 | 0.79 |
|  | 10-2723 | 20696970 | ACGTTGGATGCCCACAATGAGATGCAGATG | ACGTTGGATGAGACAAAATGCAACACTCCG | 0.13 | 0.09 | 0.12 | 0.93 |
| 11 | 11-1849 | 11974790 | ACGTTGGATGCGCCACTCTTCCTGATTTAG | ACGTTGGATGACAGATACGGGAGGCATTTC | 0.28 | 0.14 | 0.24 | 0.82 |
|  | 11-3935 | 28434679 | ACGTTGGATGATCCCTGAGACTTTGGATGG | ACGTTGGATGCCAACTTGAATGTCCATTCC | 0.10 | 0.03 | 0.09 | 0.95 |
|  | 11-522-1_C_214 | 3033366 | ACGTTGGATGCTACATGGTATCAGATACCG | ACGTTGGATGAGAAGCGAACGCGGAAAAAG | 0.19 | 0.07 | 0.17 | 0.89 |
| 12 | 12-1794 | 11215946 | ACGTTGGATGGTGAGCCCCAAAAGTTGGTG | ACGTTGGATGTAAGGTCCAGTTTGCTTGGT | 0.10 | 0.03 | 0.10 | 0.95 |
|  | 12-3200-2_C_389 | 21396181 | ACGTTGGATGGCTCAAACCTAGCAATAACTG | ACGTTGGATGCCTCCTTCCTACAAGTTTAA | 0.05 | 0.02 | 0.05 | 0.97 |
|  | 12-400 | 2160546 | ACGTTGGATGCCAATAGAGTCCATCTCAGC | ACGTTGGATGGCACGAGGATTTAAGACAGC | 0.27 | 0.11 | 0.23 | 0.83 |
|  |  |  |  | **mean** | **0.30** | **0.10** | **0.24** | **0.78** |
