# Supplementary material for: Analysis of Genetic Diversity and Population Structure of Rice Germplasm from North-Eastern Region of India and Development of a Core Germplasm Set
Source: PLoS One. 2014 Nov 20;9(11):e113094. doi: 10.1371/journal.pone.0113094 (PMC4239046; doi:10.1371/journal.pone.0113094)
Supplement: Table S3 — Output Table generated from Structure Harvester software for Evanno method. Yellow highlight shows the largest value in the Delta K column. (DOCX) [file pone.0113094.s008.docx]

**Table S3.** Output Table generated from Structure Harvester software for Evanno method. Yellow highlight is performed dynamically on the website and shows the largest value in the Delta K column.


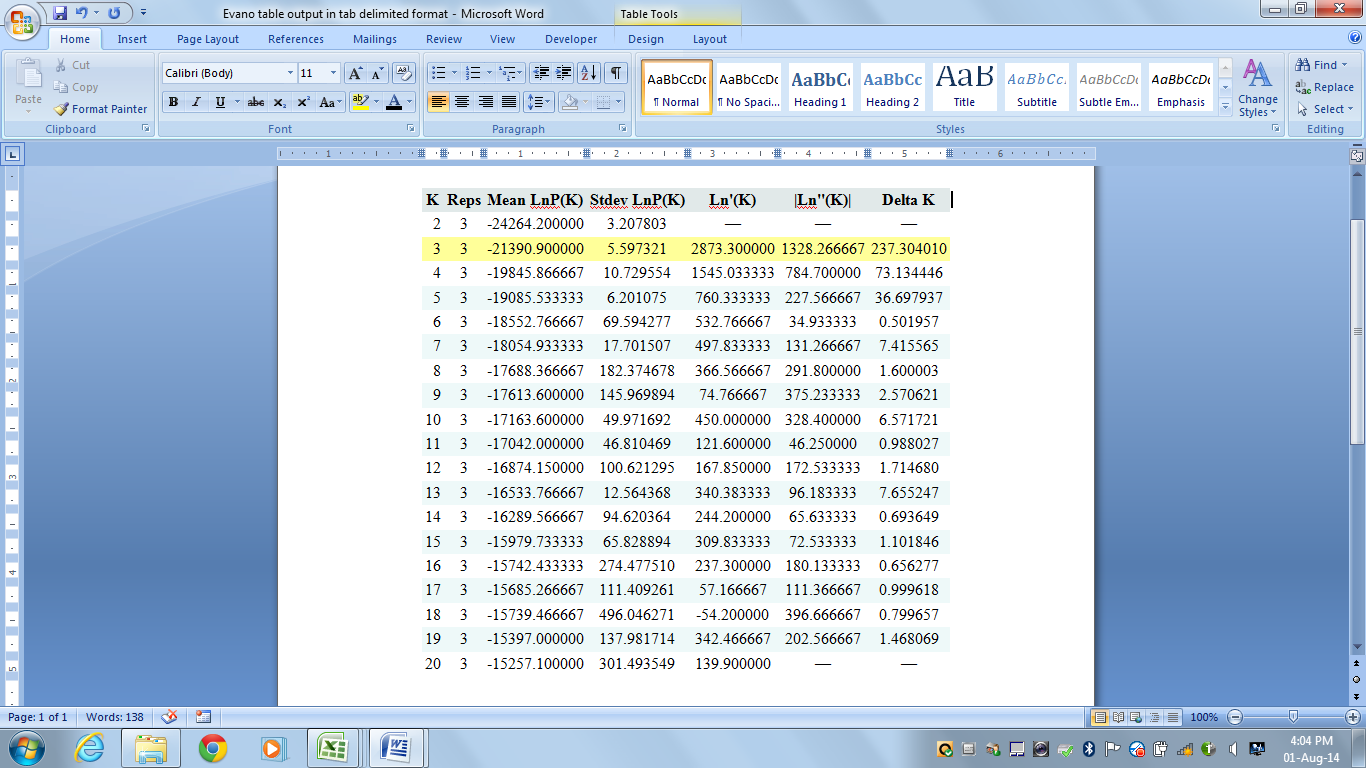
Arunachal Pradesh


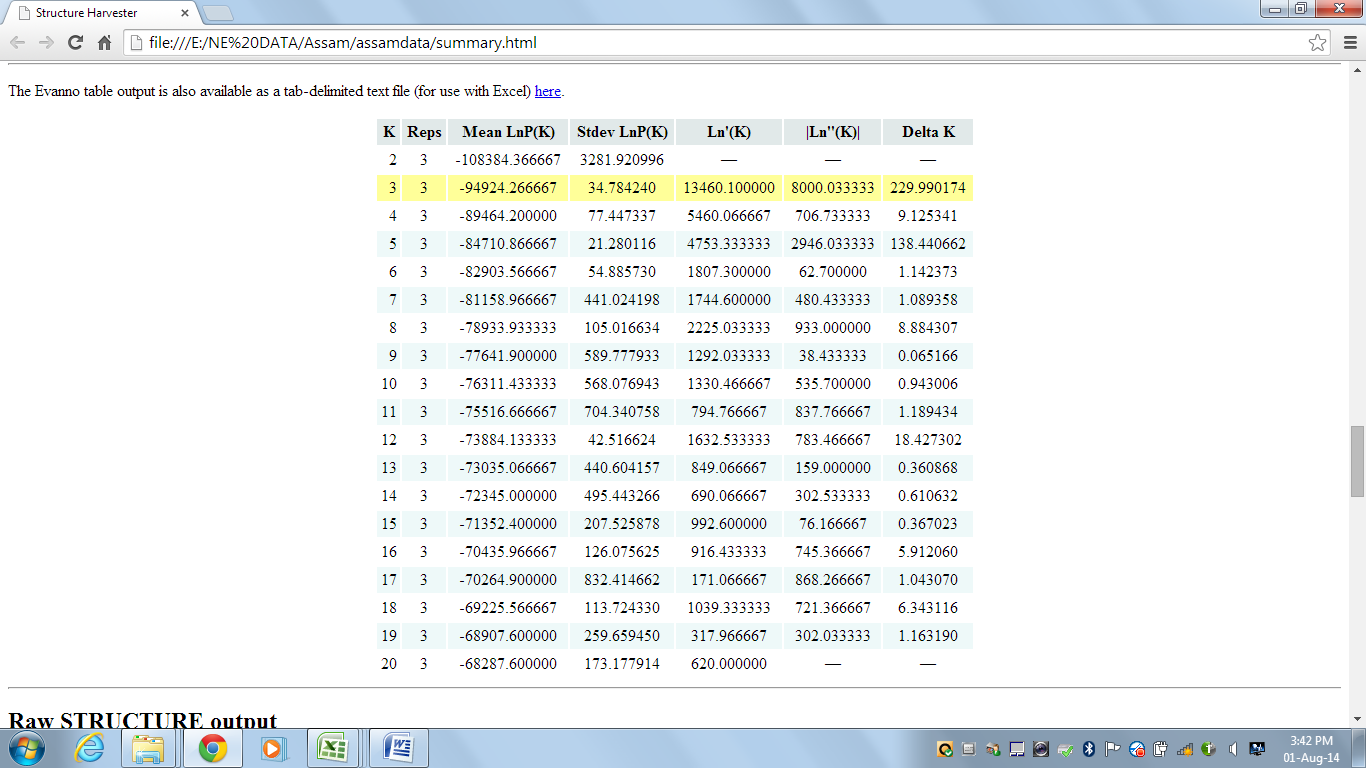
Assam


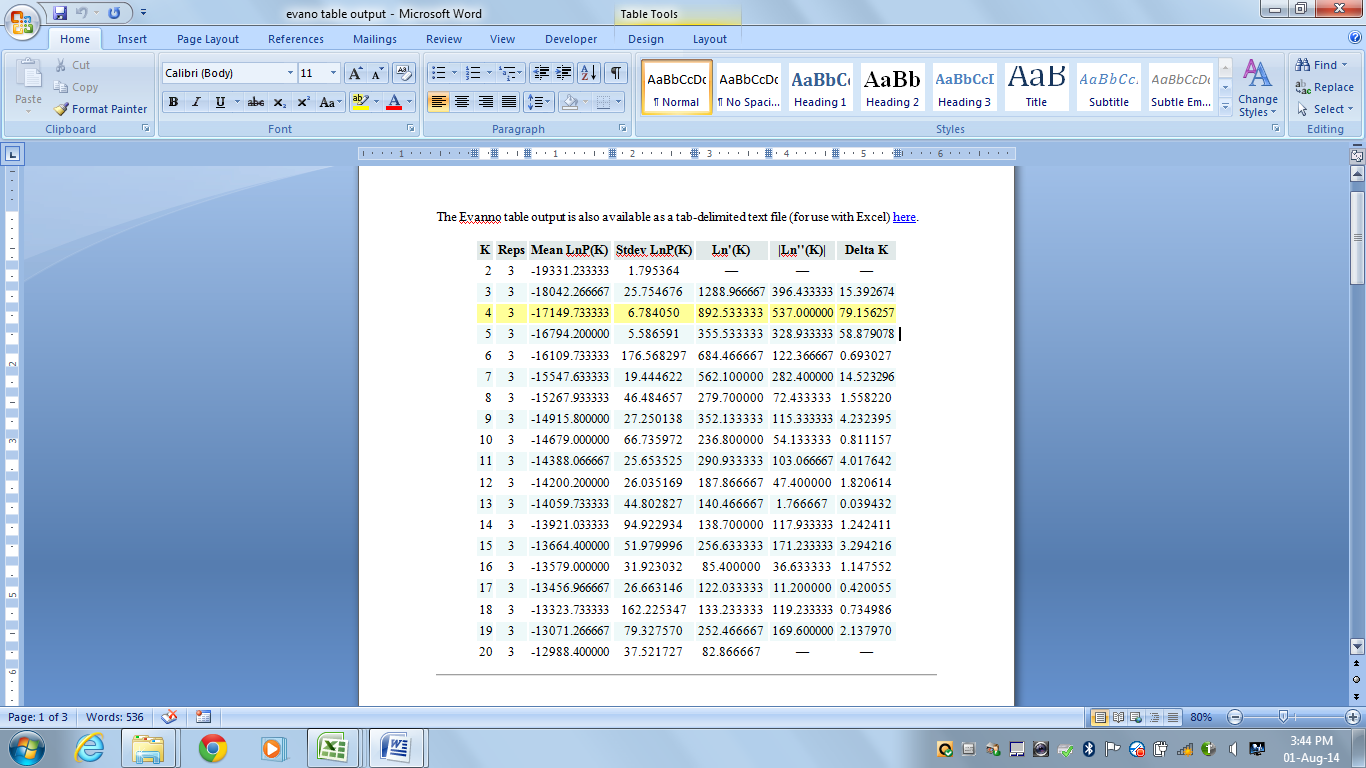
Manipur


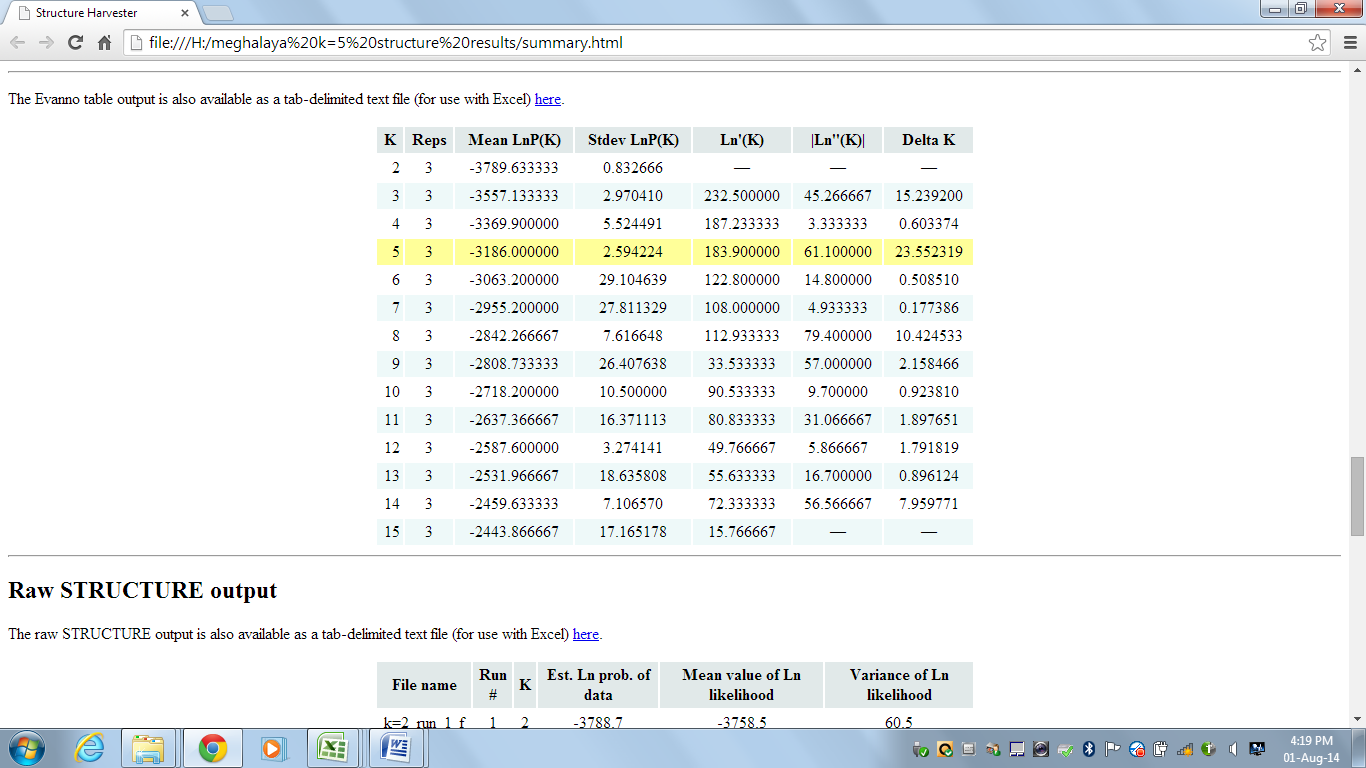
Meghalaya


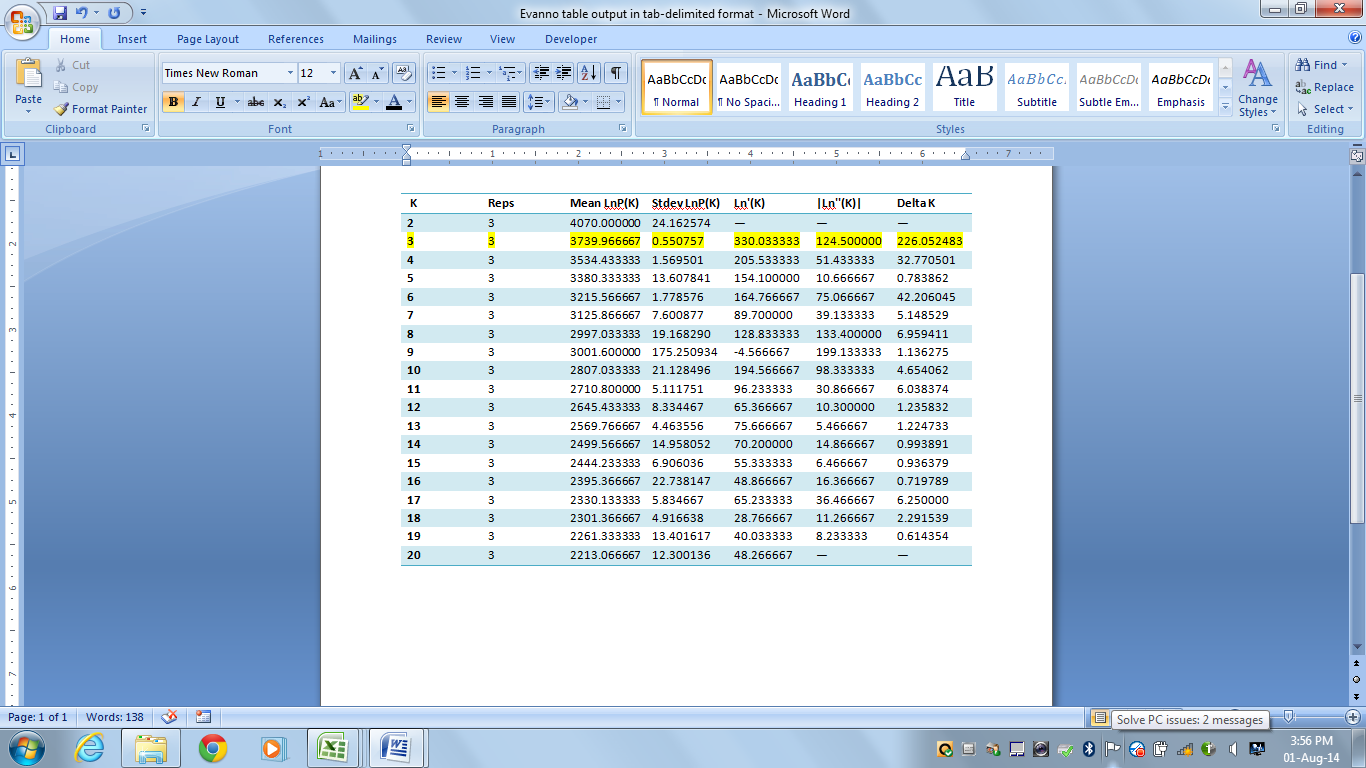


Mizoram


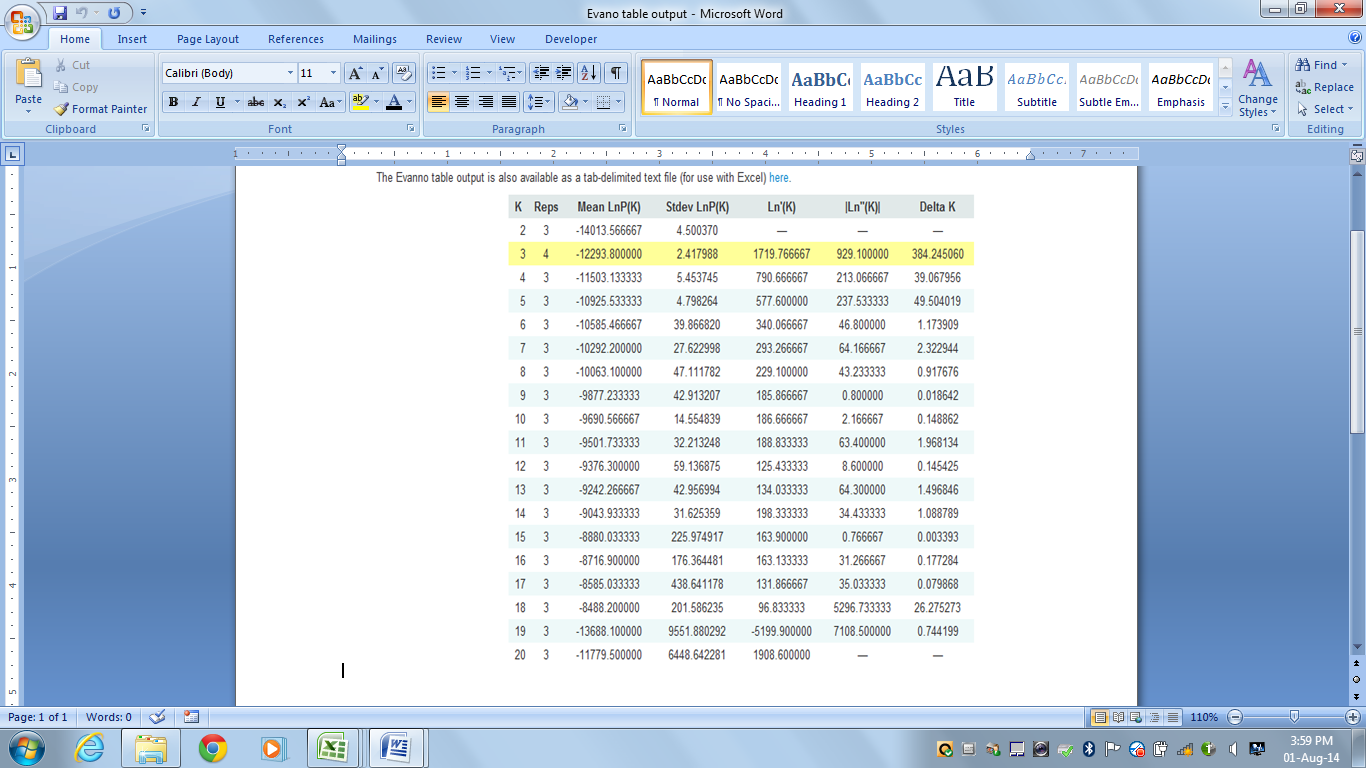
Nagaland


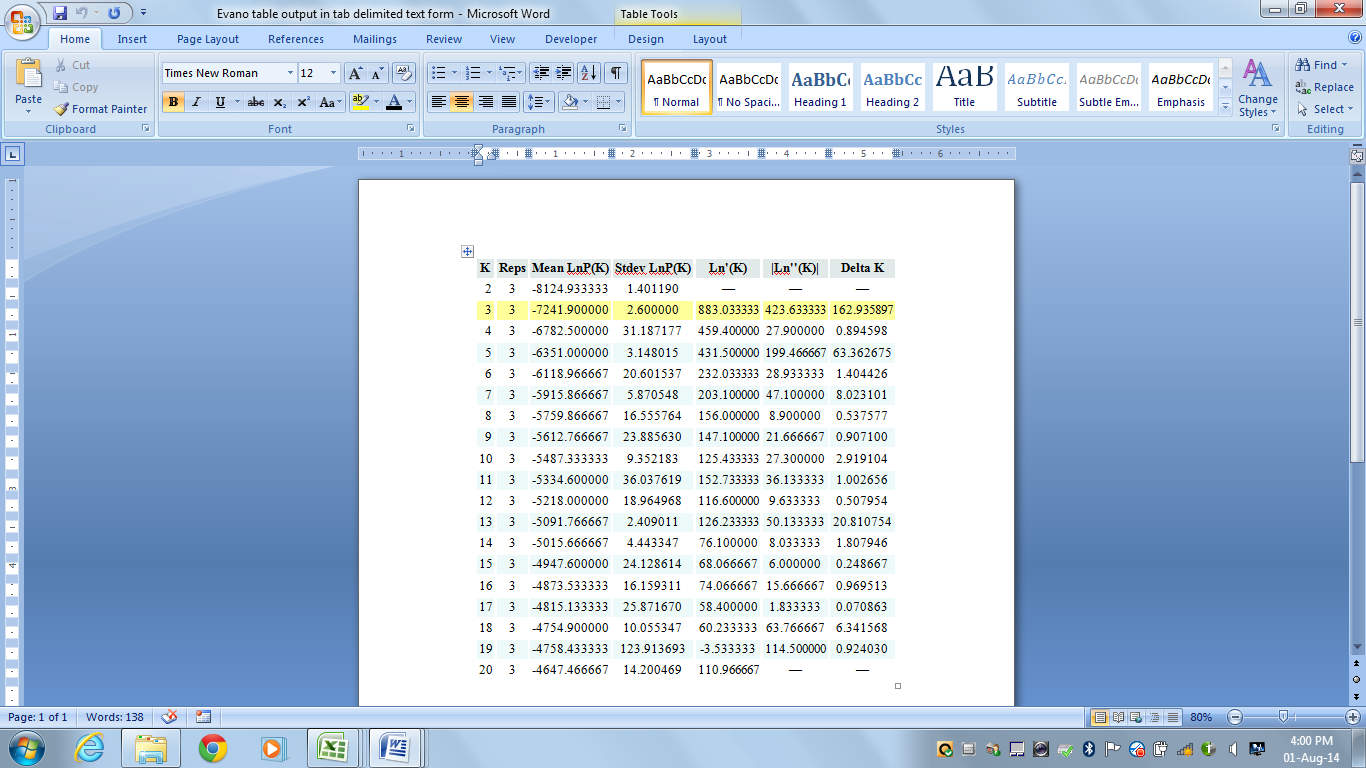
Tripura
